# DIAPH1-MFN2 interaction regulates mitochondria-SR/ER contact and modulates ischemic/hypoxic stress

**DOI:** 10.1038/s41467-023-42521-x

**Published:** 2023-10-30

**Authors:** Gautham Yepuri, Lisa M. Ramirez, Gregory G. Theophall, Sergei V. Reverdatto, Nosirudeen Quadri, Syed Nurul Hasan, Lei Bu, Devi Thiagarajan, Robin Wilson, Raquel López Díez, Paul F. Gugger, Kaamashri Mangar, Navneet Narula, Stuart D. Katz, Boyan Zhou, Huilin Li, Aleksandr B. Stotland, Roberta A. Gottlieb, Ann Marie Schmidt, Alexander Shekhtman, Ravichandran Ramasamy

**Affiliations:** 1 Diabetes Research Program, Division of Endocrinology, Diabetes and Metabolism, Department of Medicine, NYU Grossman School of Medicine, New York, New York 10016 USA; 2 Department of Chemistry, University of Albany, State University of New York, Albany, NY 12222 USA; 3 Department of Medicine, Leon H. Charney Division of Cardiology, NYU Grossman School of Medicine, New York, NY 10016 USA; 4 Department of Pathology, NYU Grossman School of Medicine, New York, NY 10016 USA; 5 Department of Population Health, NYU Grossman School of Medicine, New York, NY 10016 USA; 6 Department of Cardiology, Smidt Heart Institute, Cedars-Sinai Medical Center, Los Angeles, CA 90048 USA; 7 Department of Biomedical Sciences, Smidt Heart Institute, Cedars-Sinai Medical Center, Los Angeles, CA 90048 USA

**Keywords:** NMR spectroscopy, Cardiovascular biology, Endoplasmic reticulum, Mitochondria

## Abstract

Inter-organelle contact and communication between mitochondria and sarco/endoplasmic reticulum (SR/ER) maintain cellular homeostasis and are profoundly disturbed during tissue ischemia. We tested the hypothesis that the formin Diaphanous-1 (DIAPH1), which regulates actin dynamics, signal transduction and metabolic functions, contributes to these processes. We demonstrate that DIAPH1 interacts directly with Mitofusin-2 (MFN2) to shorten mitochondria-SR/ER distance, thereby enhancing mitochondria-ER contact in cells including cardiomyocytes, endothelial cells and macrophages. Solution structure studies affirm the interaction between the Diaphanous Inhibitory Domain and the cytosolic GTPase domain of MFN2. In male rodent and human cardiomyocytes, DIAPH1-MFN2 interaction regulates mitochondrial turnover, mitophagy, and oxidative stress. Introduction of synthetic linker construct, which shorten the mitochondria-SR/ER distance, mitigated the molecular and functional benefits of *DIAPH1* silencing in ischemia. This work establishes fundamental roles for DIAPH1-MFN2 interaction in the regulation of mitochondria-SR/ER contact networks. We propose that targeting pathways that regulate DIAPH1-MFN2 interactions may facilitate recovery from tissue ischemia.

## Abstract (web summary)

Proximity between mitochondria and endoplasmic reticulum regulates mitochondria fitness and is adversely affected by tissue ischemia. This work reveals that Diaphanous1-Mitofusin2 interaction regulates this proximity and impairs recovery in ischemia.

## Introduction

Mitochondria provide energy in the form of ATP for cellular activities and are involved in the modulation of reactive oxygen species (ROS), calcium homeostasis, apoptosis, and necrosis^1–3^. Mitochondria undergo continual cycles of fusion and fission to sustain mitochondria health^3–8^. Mitochondria quality is maintained by mitochondrial fusion, which allows the mixing of mitochondrial DNA, lipids, proteins, and metabolites to promote the retention of essential mitochondria components; and mitochondria fission, which facilitates the selective removal of damaged mitochondria by mitophagy^9^. The key proteins mediating mitochondria fusion are the mitofusins, MFN1 and MFN2^10^. Importantly, MFN2 displays a number of pleiotropic non-fusion effects, including tethering the mitochondria and the sarco/endoplasmic reticulum (SR/ER)^8,11–16^. Increasing the Mito-SR/ER distance reduces mitochondria Ca^2+^ overload, attenuates oxidative stress, and inhibits mitochondria permeability transition pore (MPT) opening; thereby protecting cells from superimposed stresses such as hypoxia and ischemia^1,17^. Despite their importance, the molecules and mechanisms that regulate Mito-SR/ER contact and its consequences are not fully defined.

We recently reported a key role for the formin, Diaphanous-1 (DIAPH1), in the pathogenesis of ischemia/reperfusion (I/R) injury^18^. Formins are nucleators of un-branched actin filaments implicated in actin cable formation^19–21^; assembly of actin filaments in the cytokinetic ring^22^; focal adhesions and adherens junctions^23^; cell migration and ruffling^24,25^; and filopodia formation^26^. DIAPH1 contains a functional diaphanous inhibitory domain (DID); formin homology domains FH1 and FH2^19,27^; and diaphanous autoinhibitory domain (DAD); and modulate actin-driven migration, phagocytosis, cellular trafficking and transcription. In the free form, the DID–DAD intramolecular interaction^20,21^ inhibits DIAPH1 actin polymerization activity. In mammalian and *Drosophila* cells, DIAPH1 has been linked to motility and trafficking of Mito^28^. Distinct experiments link DIAPH1 to metabolic dysfunctions, such as in the heart and cardiomyocytes (CMs)^18^.

Here, through biochemical, cellular, biophysical, and in vivo studies, we report that DIAPH1 interacts directly with MFN2 and that this interaction enhances Mito-SR/ER contact, thereby regulating mitochondria turnover, mitophagy, and oxidative stress. This work identifies a fundamental role for DIAPH1 in the Mito-SR/ER contact networks that control responses to stress and suggests that targeting pathways that regulate DIAPH1–MFN2 interaction may facilitate recovery from tissue ischemia.

## Results

### DIAPH1 interacts directly with MFN2 to regulate Mito-SR/ER distance

To test the hypothesis that the formin DIAPH1 regulates responses to ischemic stress through modulation of mitochondria properties, we employed human induced pluripotent stem cells (HiPSCs) differentiated into CMs (HiPSC-CMs)^29–32^. Bright-field microscopy confirmed typical HiPSC colony morphology with clean edges (Supplementary Fig. 1a). Immunofluorescence studies validated the reprogramming of HiPSCs with expression of pluripotency markers, OCT4, SOX2, and NANOG (Supplementary Fig. 1a). Silencing of *DIAPH1* was achieved in HiPSCs using lentiviral shRNA particles vs. control particles containing scrambled (Scr) sequences targeting non-mammalian genes. The HiPSCs were successfully differentiated into CMs, which showed synchronized beating (Supplementary Fig. 1b, Supplementary Movies 1 and 2). After 25 days of differentiation, HiPSC-CMs were >90% double positive for both TNT2 and TNNI3 (adult), which are markers of mature CMs^29–32^, as determined by flow cytometry (Supplementary Fig. 1c).

**Fig. 1:**
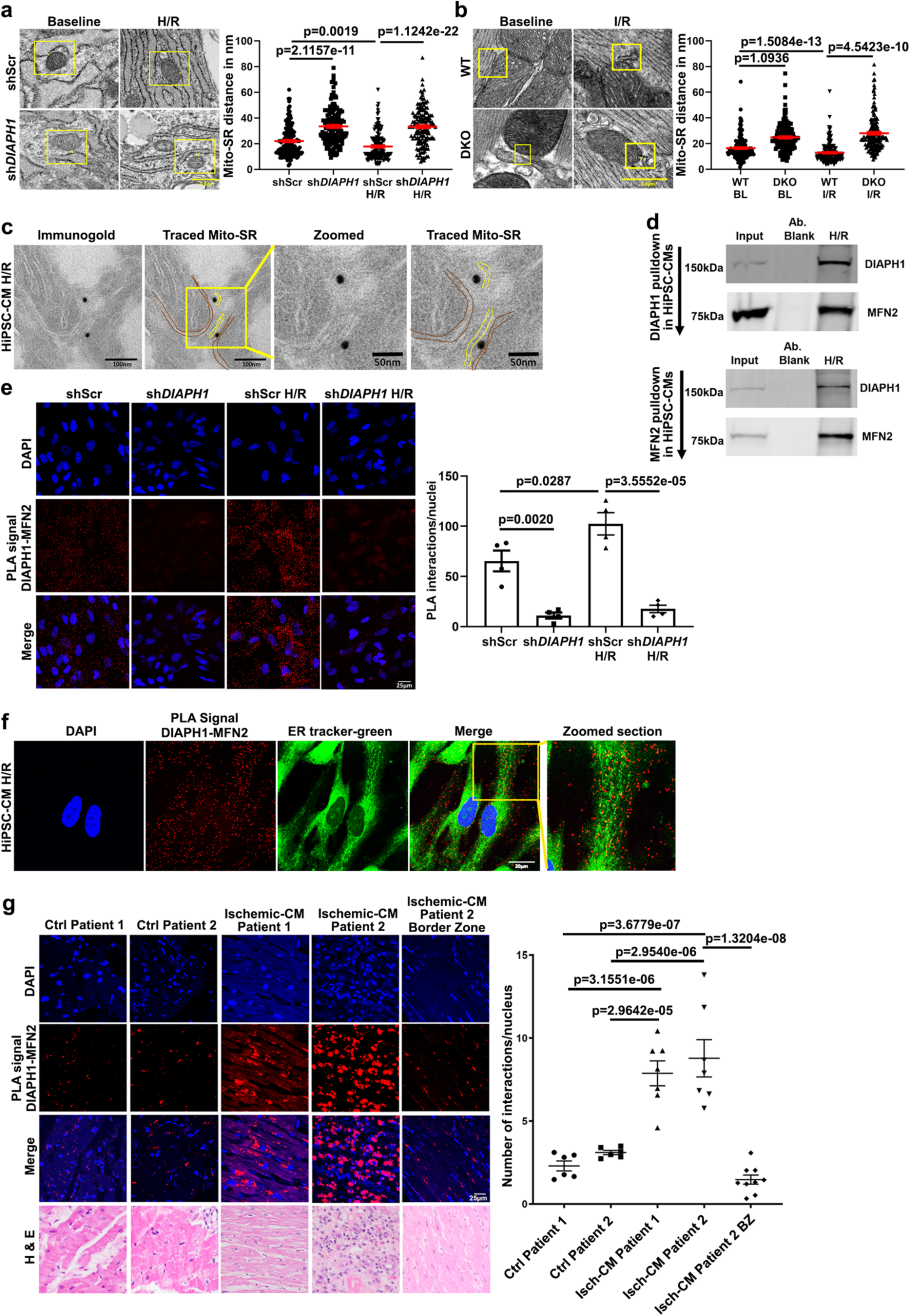
DIAPH1 regulates Mitochondria(Mito)-SR/ER distance through interaction with MFN2. TEM imaging was performed to measure Mito-SR/ER distance **a** shScr and sh*DIAPH1* hiPSC-CMs under normal and H/R conditions and **b** in perfused WT and DKO mice hearts under baseline or I/R conditions. The distance was measured using NIH ImageJ software. Scale bar 0.5 µm. (for **a** and **b**, Mito-SR/ER distance (in nm) from four biologically independent samples, Krushal–Wallis with Dunn’s pairwise comparison test was performed for *p* values) **c** Immuno-electron microscopy study showing DIAPH1 Immunogold particles located in close proximity with Mito and SR in HiPSC-CMs exposed to H/R. Scale bar 100 nm and 50 nm. **d** DIAPH1 and MFN2 pull-down assay in HiPSC-CMs exposed to H/R. Upper panel represents DIAPH1 pulldown followed by Western blot detection with MFN2 antibody and the lower panel represents MFN2 pulldown with DIAPH1 antibody detection. Input refers to the total lysate, Ab blank refers to IP sample without DIAPH1/MFN2 antibody **e** Leica SP8 Confocal microscopy images of fluorescent DUOLINK proximity ligation assay (PLA) signal of DIAPH1–MFN2. Interactions are represented as red dots and corresponding quantification was represented by number of positive interactions per nucleus in shScr and sh*DIAPH1* hiPSC-CMs under baseline and H/R. Scale bar 25 µm. **f** Leica SP8 confocal microscopy images of HiPSC-CMs under H/R stained with live staining ER-tracker green followed by co-staining with DUOLINK PLA red to detect SR–DIAPH1–MFN2 localization. Scale bar 20 µm. **g** DIAPH1-MFN2 DUOLINK PLA interactions in human heart biopsy sections (ischemic and non-ischemic) Scale bar 25 µm. The control samples were post-transplant endomyocardial biopsy and all other samples were sections of left ventricle from heart explants of patients with ischemic cardiomyopathy. Corresponding closest matched sections to DUOLINK for H&E staining was used to detect muscle morphology. Magnification for H&E sections were at 40×, 40×, 20×, 40×, and 20× respectively, in the order mentioned in the figure. For human biopsy samples, *n* represents random images taken from each tissue biopsy(for *p* values *t*-test with pooled SD was performed to compare between different patient biopsies. Unpaired *t*-test was performed between groups Isch-CM patient 2 vs. Isch-CM patient 2). Data are presented as the mean ± SEM. Statistics file and source data are provided as a Source Data file.

We subjected these cells to transmission electron microscopy (TEM) to determine the distance between mitochondria and SR/ER in *DIAPH1*-silenced vs. Scr HiPSC-CMs. In the baseline condition, Mito-SR maintains a mean distance of ~22.04 nm in shScr cells, which was significantly higher, at 33.42 nm, in sh*DIAPH1* HiPSC-CMs; *p* < 0.001 (Fig. 1a). To mimic I/R injury in vitro, HiPSC-CMs were exposed to 30 min hypoxia (0.1% O_2_) followed by reoxygenation for 1 h (H/R). Under H/R, the Mito-SR distance in shScr cells was shortened to 17.77 nm compared to the baseline in shScr cells (22.04 nm); *p* < 0.001. In sh*DIAPH1* HiPSC-CMs in H/R, the Mito-SR distance was maintained at 33.22 nm vs. the shScr HiPSC-CMs; *p* < 0.001 and did not significantly differ when comparing baseline vs. H/R stress conditions (Fig. 1a). Thus, silencing *DIAPH1* in HiPSC-CMs reduces Mito-SR contact under baseline and H/R conditions.

These concepts were also tested in a rat myoblast cell line derived from embryonic BD1X rat heart tissue, H9C2 cells. At baseline, a mean distance of 20.11 nm was observed in shScr control cells, which was significantly higher in sh*DIAPH1* cells, 32.59 nm; *p* < 0.001 (Supplementary Fig. 1d). We analyzed the Mito-SR/ER distance in perfused WT and global *Diaph1*-deleted mice hearts (DKO). Under baseline conditions, the Mito-SR/ER distance was significantly lower in the WT hearts, at 16.49 nm, compared with the DKO hearts at the baseline, 24.84 nm; *p* < 0.001 (Fig. 1b). Under I/R conditions, the distance was further reduced to 12.85 nm in WT hearts compared with 28.05 nm in DKO hearts; *p* < 0.001 (Fig. 1b).

Next, to determine the localization of DIAPH1 in mitochondria and SR/ER, we performed immune-electron microscopy in HiPSC-CMs exposed to H/R, which localized DIAPH1 immunogold particles in between mitochondria and SR (Fig. 1c). We also employed HEK cells transfected with constructs expressing RFP-tagged DIAPH1, emerald-tagged ER, and CFP-tagged mitochondria and observed localization of DIAPH1 with both mitochondria and ER (Supplementary Fig. 1e).

To determine how DIAPH1 influences the Mitochondria–SR/ER distance, we focused on MFN2, the key tethering protein, which anchors both in mitochondria and in SR/ER and is known to regulate the distance between the two organelles^8,12–16^. Immunoprecipitation (IP) studies were performed in the shScr HiPSC-CMs exposed to H/R and subjected to IP with antibodies to either DIAPH1 or MFN2 followed by Western blotting with antibodies to MFN2 or DIAPH1; these studies revealed that DIAPH1 interacts with MFN2 (Fig. 1d upper panel and lower panel). IP with antibodies to DIAPH1 in HiPSC-CMs revealed that DIAPH1 does not interact with MFN1 (Supplementary Fig. 1f).

To further investigate the direct interaction between DIAPH1 and MFN2, DUOLINK® proximity ligation assay (PLA) was performed. PLA is an established technique to study endogenous protein–protein interactions in cells and fixed tissue^1,33,34^. In this PLA, DIAPH1–MFN2 interactions are detected as red signals/dots, and the number of red dots indicates the number of interactions. In shScr HiPSC-CMs, DIAPH1 interacts with MFN2, as shown by the red dots, and the quantified number of interactions was significantly higher under H/R conditions; *p* < 0.05 (Fig. 1e). To determine the specificity of this assay for the interaction of DIAPH1 with MFN2, we silenced *DIAPH1* in both baseline and H/R conditions and observed a significant reduction of DIAPH1-MFN2 interactions compared to shScr controls; *p* < 0.01 and *p* < 0.001, respectively (Fig. 1e).

These concepts were tested in other cell types as well, including rat H9C2 cells, human microvascular endothelial cells (HMVECs), and mouse bone marrow-derived macrophages (BMDMs). Silencing of *Diaph1*/*DIAPH1* was achieved in rat H9C2 cells, *p* < 0.001; HMVECs, *p* < 0.001; and mouse BMDMs, *p* < 0.05 (Supplementary, Fig. 1g). In each of these cell types, silencing of *Diaph1/DIAPH1* resulted in significantly reduced DIAPH1–MFN2 interactions by PLA in H9C2 cells, *p* < 0.05; HMVECs, *p* < 0.05; and BMDMs, *p* < 0.05 (Supplementary Fig. 1h–j, respectively).

As DIAPH1 contributes to the regulation of F-actin polymerization, we sought to determine if the interaction of DIAPH1 with MFN2 is linked to actin polymerization. First, we assessed F and G actin by phalloidin staining. These studies revealed a significant reduction in the F:G actin ratio in sh*DIAPH1* HiPSC-CMs compared to shScr controls under baseline and H/R conditions; *p* < 0.001 and *p* < 0.05, respectively (Supplementary Fig. 1k). Second, to establish potential roles for F-actin polymerization in regulation of DIAPH1–MFN2 interaction, we inhibited actin polymerization using Latrunculin B (LATB). Treatment of non-transduced HiPSC-CMs with LATB significantly reduced DIAPH1–MFN2 PLA interactions under baseline and H/R conditions compared to vehicle (VEH); *p* < 0.01 and *p* < 0.001, respectively (Supplementary Fig. 1l). These data suggested that actin polymerization contributes to DIAPH1–MFN2 interactions in HiPSC-CMs.

We next performed live cell imaging for SR localization and confirmed that DIAPH1–MFN2 interactions were predominantly localizing on or around SR staining (Fig. 1f). To determine if other isoforms, DIAPH2 or MFN1, were involved in these interactions, we performed DUOLINK PLA and found that there we no observed interactions between DIAPH1 with MFN1 or between MFN2 with DIAPH2 (Supplementary Fig. 2a, b, respectively).

Furthermore, since interactions involving VDAC1, GRP75, IP3R, and RhoA are known to affect the contact between Mito-SR, we performed PLA between DIAPH1 and these proteins. Our data revealed that DIAPH1 did not interact under baseline or H/R conditions with VDAC1, GRP75, and IP3R. However, we observed very few non-quantifiable interactions with RhoA only under H/R conditions (Supplementary Fig. 3a–d). Under H/R conditions, we did observe VDAC1–IP3R interactions, which were significantly reduced by silencing *DIAPH1*. These data further underscore the importance of DIAPH1 in regulating Mito-SR distance; *p* < 0.05 (Supplementary Fig. 3e).

We next probed human heart biopsy sections for DIAPH1–MFN2 interaction using PLA. Compared to hearts retrieved from control (Ctrl) patients 1 and 2, patients with ischemic heart disease demonstrated a significantly higher number of DIAPH1–MFN2 interactions; *p* < 0.001 (Fig. 1g). In patient 2, the heart sections from the ischemic injury zone demonstrated significantly more DIAPH1–MFN2 interactions compared to those in the border zone (bz) region from the same section but without injury; *p* < 0.001 (Fig. 1g). These data indicate that DIAPH1 interacts with MFN2 in multiple human, rat, and mouse cells as well as in human hearts. In in vitro and heart tissue samples, the interactions between DIAPH1 and MFN1 are higher under H/R conditions and in the ischemic vs. normal human heart.

### Structural basis of DIAPH1–MFN2 interaction

**Fig. 2:**
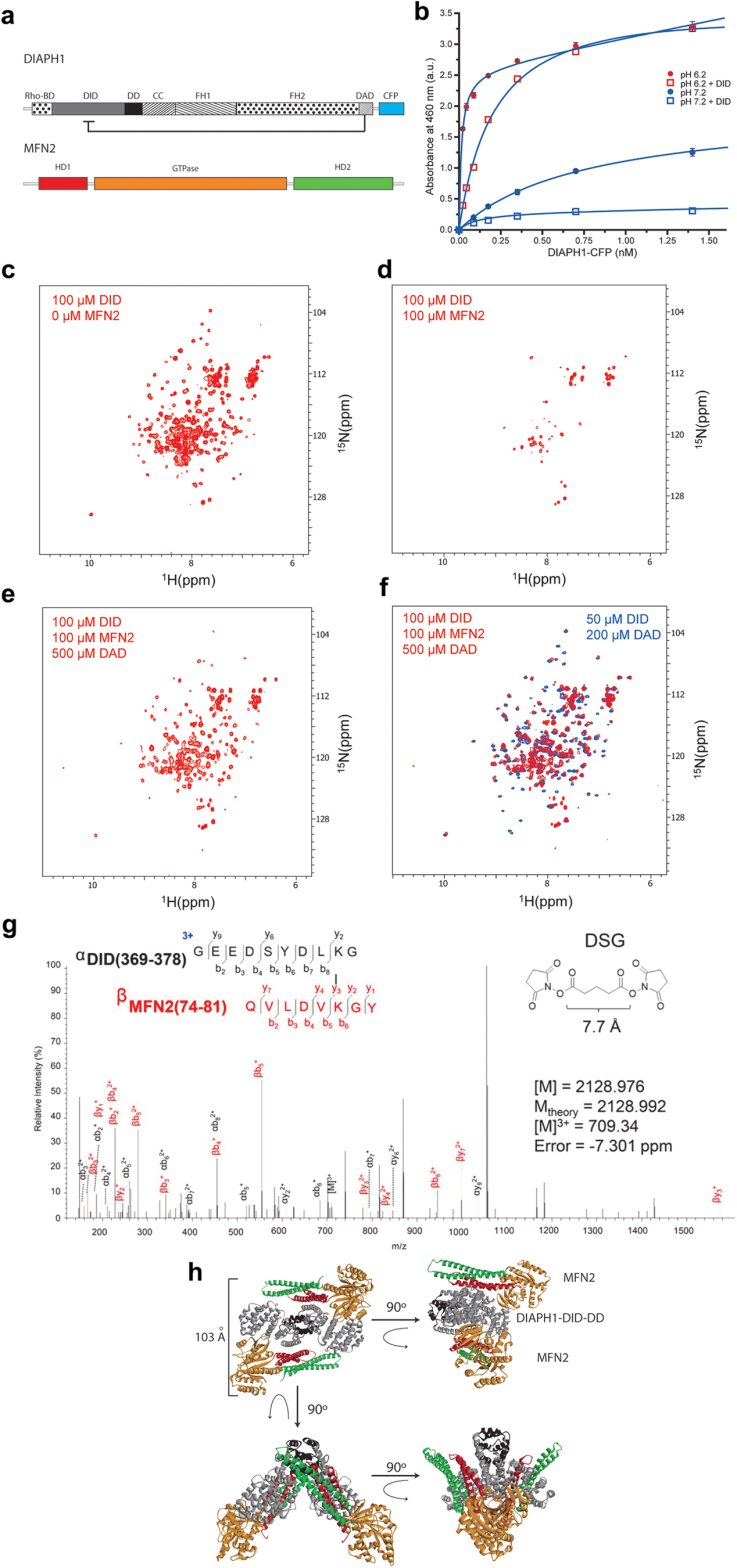
Structural characterization of DIAPH1–MFN2 interaction. **a** Domain structure of the studied proteins. DIAPH1–CFP domains are Rho-BD, Rho GTPase binding domain; DID; DD, dimerization domain; CC, coiled-coil; FH1/2; DAD; and CFP, cyan fluorescent protein, respectively. MFN2 domains are HD1/2, helical domains 1 and 2; and GTase domain. The autoinhibitory intramolecular DID–DAD interaction is indicated. **b** Binding of the cytosolic MFN2 with DIAPH1-cyan fluorescent protein, CFP, under neutral (blue circles), *K*_d_ = 0.7 ± 0.1 nM, and acidic (red circles), *K*_d_ = 0.01 ± 0.003 nM, conditions. The binding in the presence of the competitor, DID, is shown by the blue (no binding) and red (*K*_d_ = 0.2 ± 0.1 nM) squares under neutral and acidic conditions, correspondingly. Data are presented as mean values ± SEM. **c**–**f**. Structural basis of the DID–MFN2 interaction probed by NMR spectroscopy. ^1^H-^15^N-HSQC spectra of [*U*-^15^N]-DID upon titration with MFN2 and DAD peptide at 305 K. Amide proton–nitrogen cross-peaks from the protein backbone and side chains of 100 µM [*U*-^15^N]-DID are broadened upon addition of MFN2 (**c**, **d**). Adding DAD peptide (500 µM) restores the cross peaks of DID (**e**, **f**). In panel **f**, red peaks correspond to the NMR spectrum of 100 µM [*U*-^15^N]-DID with 100 µM MFN2 and 500 µM DAD, while blue peaks correspond to the NMR spectrum of 50 µM [*U*-^15^N]-DID with 200 µM DAD. **g** A representative high-energy collision MS spectrum was obtained for the DSG cross-linked product between MFN2 and DID at 709.34 m/z. The inlay shows the sequence and composition of cross-linked peptides and an experimental mass of 2128.976 Da, which is in good agreement with the theoretical mass of 2128.992 Da calculated using putative elemental composition. Standard nomenclature was used in labeling charge states and fragment ions. MS data were deposited to ProteomeXchange, https://www.proteomexchange.org/, and jPOST, https://repository.jpostdb.org/, sites with accession numbers PXD045744 and JPST002335, respectively. **h** Representative ribbon model of the docked MFN2:DIAPH1–DID–DD tetrameric complex. MFN2 and DIAPH1-DID-DD are colored according to the domain structure of panel (**a**). Source data are provided as a Source Data file.

To further demonstrate the interaction of DIAPH1 with MFN2, we utilized several additional strategies. We employed an enzyme-linked immunosorbent assay (ELISA) to study the DIAPH1–MFN2 interaction. The binding affinity, expressed as the dissociation constant, *K*_D,_ of DIAPH1 for the purified cytosolic fragment of MFN2, including GTPase domain^35^, was 0.7 ± 0.1 nM at pH 7.2 (Fig. 2a, b, Supplementary Table 1). Adding 1 µM of purified DID to the solution (Fig. 2a, b) attenuated the DIAPH1–MFN2 binding at pH 7.2, suggesting that MFN2 binding is largely through the DID of DIAPH1, although binding sites to other DIAPH1 domains cannot be excluded. Decreasing the solution pH from 7.2 to 6.2 increased the binding affinity to 0.01 ± 0.003 nM, suggesting that DIAPH1–MFN2 electrostatic interactions play an important role. Importantly, consistent with the observation at pH 7.2, the addition of 1 µM of DID to the solution at pH 6.2 reduced the DIAPH1–MFN2 binding affinity to 0.22 ± 0.01 nM (Fig. 2b).

To further characterize the DIAPH1–MFN2 interaction, NMR titrations, cross-linking mass spectrometry, and computational docking experiments were performed. Peaks in the ^1^H-^15^N-heteronuclear single quantum coherence (HSQC) spectra of uniformly ^15^N labeled [*U*-^15^N]-DID correspond to amide proton-nitrogen pairs in the backbone and side chains of the protein (Fig. 2c–f)^36^. As MFN2 was added to [*U*-^15^N]-DID using mole ratios from 1:0.25 to 1:1 (DID:MFN2), a global broadening of DID cross-peaks was observed (Fig. 2d). These data suggest an interaction between DID and MFN2 that falls in the intermediate exchange regime of NMR, usually described by binding affinity values less than 10 µM^36^. The addition of five molar equivalents of DAD to the DID–MFN2 mixture reversed this effect (Fig. 2e, f), indicating significant overlap of the DID–DAD and DID–MFN2 binding interfaces. No interaction was detected between DAD and MFN2 (Supplementary Fig. 4a).

Seven cross-linkers (N-κ-maleimidoundecanoyl-oxysulfosuccinimide ester (KMUS), succinimidyl 4-(p-maleimidophenyl)butyrate (SMPB), succinimidyl-4-(*N*-maleimidomethyl)cyclohexane-1-carboxylate (SMCC), bis(sulfosuccinimidyl)suberate (BS_3_), disuccinimidyl glutarate (DSG), bis-*N*-succinimidyl-(pentaethylene glycol) ester (BS(PEG)_5_), and bis-*N*-succinimidyl-(nonaethylene glycol) ester BS(PEG)_9_) with various spacer lengths were used to obtain structural information for the complex formed by DID with MFN2. SDS-PAGE analysis of cross-linking reactions employing the heterobifunctional (amine-to-sulfhydryl) cross-linkers KMUS, SMPB, and SMCC indicated the presence of a 1:1 DID–MFN2 complex represented by a band with an apparent mass of ~75 kDa only for the conditions that used SMCC (Supplementary Fig. 4b). This cross-linked 1:1 complex was also observed in the reactions involving homobifunctional (amine-to-amine) cross-linkers DSG and BS(PEG)_9_ but was seemingly absent for BS(PEG)_5_ and BS_3_ (Supplementary Fig. 4c–f). For all cross-linking reaction conditions tested, the area of the SDS-PAGE gel corresponding to ~70 kDa was excised and subjected to trypsin digestion and mass spectrometric analysis. The five positive intermolecular (DID–MFN2) cross-links identified by MS and tandem MS/MS (Fig. 2g, Supplementary Fig. 4g, Supplementary Table 2) placed residue pairs K377–K79 within 7.7 Å, K143–K192 within 35.8 Å, and K228–C132/C164–K37/C164–K158 within 8.3 Å. These distances correspond to cross-linking reactions involving DSG, BS(PEG)_9_, and SMCC, respectively, indicating that the results of MS and SDS-PAGE are consistent and that the identified peptide-linker conjugates are not false positive findings.

We combined our findings from NMR titrations and cross-linking MS to formulate distance constraints for the computational docking of DIAPH1, comprised of the DID–DD dimer, to MFN2 (Fig. 2h, Supplementary Fig. 4h, i, Supplementary Tables 2 and 3). Specifically, by NMR, we observed that the DID–DAD binding interface is putatively involved in DID–MFN2 binding, therefore we defined ambiguous interaction restraints (AIRs) between DID and MFN2 that involved residues of DID in close proximity of DAD (within 5 Å in a crystal structure model of the mouse homolog of DIAPH1, mDia1 DID–DAD complex, PDB entry 2F31^37^). On the other hand, we defined unambiguous interaction restraints (UIRs) based on the distances between residue pairs identified by cross-linking MS. We carried out two docking protocols, one using both AIRs and UIRs and another using only UIRs (Supplementary Fig. 4h, i, Supplementary Tables 2–7). Docking using both protocols resulted in structural models of the DIAPH1-MFN2 tetrameric complex (Fig. 2h, Supplementary Fig. 5a, b) in which the GTPase domain and HD1 domains of each MFN2 monomer are in contact with a monomer of DID. In a representative model of the tetrameric complex, the longest distance between an atom on the GTPase domain of MFN2 of the first MFN2 monomer and an atom on the helical domain (HD1/2) of the second MFN2 monomer is about 103 Å (Fig. 2h and Supplementary Fig. 5a–c).

In the representative docked complex (Supplementary Fig. 5c, d), it is evident that the positively charged binding surface of MFN2 is complementary to the negatively charged surface of DID, and the binding interface between the GTPase and HD1 domains of MFN2 and DID overlaps with the known binding interface between DID and DAD. The binding interface involves salt bridges (Supplementary Fig. 5d), hydrophobic contacts, and hydrogen bonds (Supplementary Table 5). The theoretical pIs of DID and MFN2 (5.1 and 6.6, respectively)^38^ imply that at pH 7.2, both DID and MFN2 are negatively charged; at pH 6.2, DID is negatively charged and MFN2 is positively charged. This increase in electrostatic attraction is consistent with the observed increase in the binding affinity of DIAPH1 for MFN2 at the increased acidity of the solution (Fig. 2b). In addition, the presence of specific residues at the interface with a pK_a_ between 6 and 7, such as His 142 of MFN2, that change charge states with this pH change, could modulate the binding affinity of the complex. Taken together, these data reveal a strong interaction between the DID of DIAPH1 and the cytosolic domain containing the GTPase domain of MFN2.

### Induction of hypoxia/reoxygenation in HiPSC-CMs and the effect of *DIAPH1*

**Fig. 3:**
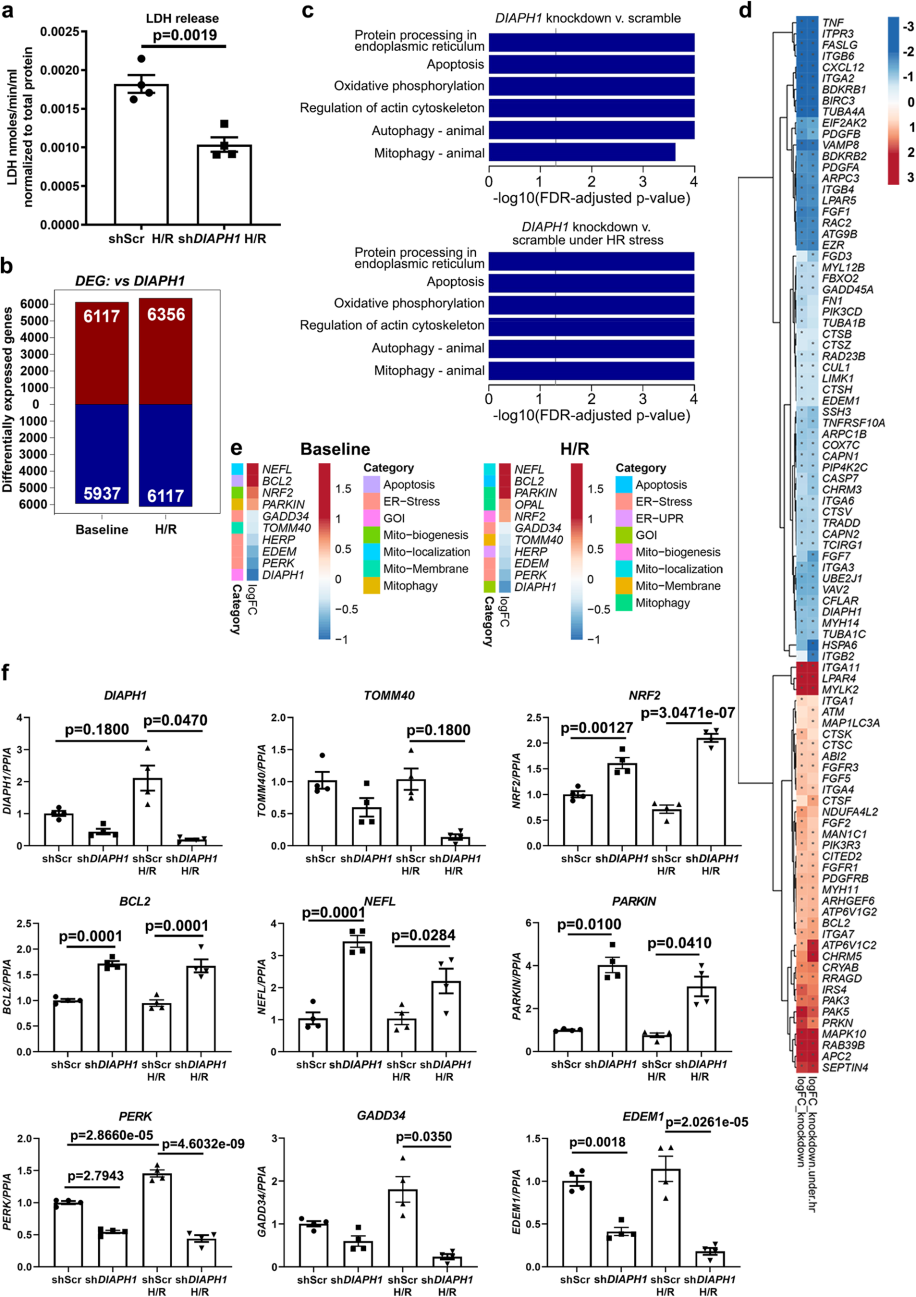
Genomics data establish a link between DIAPH1 and Mito-SR/ER function. **a** LDH release was measured in the supernatant of sh*DIAPH1* HiPSC-CMs compared to shScr HiPSC-CMs exposed to 30 min hypoxia and 60 min reoxygenation (H/R) to mimic ischemic injury (n represents four biologically independent samples, unpaired *t*-test was performed for *p* values). **b** Bulk RNAseq studies to show the total number of DEGs in shScr and sh*DIAPH1* HiPSC-CMs under baseline and H/R conditions. **c** KEGG pathway enrichment analysis was performed to show differentially regulated pathways, and **d** heat maps showing the top 50 up and downregulated genes related to mitochondria and SR/ER function (data obtained from four biologically independent samples). **e** Heat maps showing DEGs specific to mitochondria and SR/ER stress from more sensitive and targeted nCounter technology by NanoString (data obtained from four biologically independent samples). **f** qPCR validation of SR/ER stress and mitochondria markers confirmed by Bulk RNAseq and NanoString technology (*n* represents four biologically independent samples. Shapiro–Wilk test normality test was performed for all groups, following tests were performed for each gene respectively; Welch’s ANOVA with Games–Howell pairwise comparison Test for *DIAPH1*, *GADD34*, and *PARKIN*, Krushal–Wallis with Dunn’s pairwise comparison test for *TOMM40* and ANOVA with TukeyHSD pairwise comparison *for NRF2*, *BCL2*, *NEFL*, *PERK*, and *EDEM1*). Data are presented as the mean ± SEM. Source data are provided as a Source Data file.

Having established that DIAPH1 binds to MFN2, we next sought to determine the impact on cellular properties, particularly in ischemia. Lactate dehydrogenase (LDH) release was determined from HiPSC-CMs exposed to H/R as a measure of tissue injury. Significantly lower LDH release was observed in sh*DIAPH1* cells vs. the shScr controls in H/R; *p* < 0.01 (Fig. 3a).

To elucidate the DIAPH1-dependent mechanisms governing the CM response to H/R, we examined gene expression in sh*DIAPH1* CMs and shScr CMs under baseline (normoxia) and H/R conditions. Bulk RNA-sequencing (RNA-seq) studies revealed robust differential expression of genes upon silencing of *DIAPH1* with *p* ≤ 0.05 and false discovery rate (FDR) ≤ 0.05. Principal component analysis (PCA) demonstrated discrete patterns of gene expression under normoxia vs. H/R in sh*DIAPH1* vs. shScr CMs (Supplementary Fig. 6). In sh*DIAPH1* CMs vs. shScr CMs, in baseline, 6117 genes were upregulated and 5937 genes were downregulated and under H/R, 6356 genes were upregulated and 6117 genes were downregulated (Fig. 3b). Geneset analyses using Roast (rotation gene sets tests) and Camera (competitive gene set tests) revealed that multiple pathways related to mitophagy, mitochondria function/mobilization, actin cytoskeleton, ER stress, autophagy, oxidative stress, apoptosis, and calcium regulation were differentially expressed in sh*DIAPH1* CMs vs. shScr CMs (Fig. 3c, d). Using a sensitive and customized NanoString nCounter mRNA expression panel for mitochondria and ER markers, validation of the bulk RNA-seq data was performed. NanoString analyses demonstrated down-regulation of SR stress markers and favorable regulation of markers related to mitochondrial function in sh*DIAPH1* CMs vs. shScr CMs (Fig. 3e). Real-time quantitative PCR experiments confirmed the efficiency of knockdown of *DIAPH1* in the HiPSC-CMs and a significant increase in DIAPH1 expression under H/R conditions compared to baseline (Fig. 3f, 1st upper left panel). In baseline and H/R conditions, a significantly lower expression of ER stress and unfolded protein markers such as *PERK* and *EDEM1*, along with significantly higher expression of the mitophagy marker *PARKIN1*; the mitochondrial biogenesis marker, *NRF2*; mitochondrial anti-apoptosis marke*r, BCL2*; and mitochondria localization marker, *NEFL* was observed in sh*DIAPH1* CMs vs. shScr CMs; *p* < 0.05 (Fig. 3f). In contrast, there were no significant differences in the mRNA expression of *GADD34* or TOMM40 in the baseline condition, but the mRNA expression of *GADD34* and *TOMM40* was significantly lower in the sh*DIAPH1* CMs vs. shScr CMs subjected to H/R; *p* < 0.05 (Fig. 3f).

At the protein expression level, we observed significantly lower expression of DIAPH1 in the *DIAPH1*-silenced group vs. shScr controls and in shScr cells, a significant increase in DIAPH1 protein expression in HiPSC-CMs exposed to H/R stress was observed compared to baseline shScr controls (Supplementary Fig. 7a). We observed significantly higher protein expression of the mitochondrial biogenesis marker NRF2, mitophagy canonical markers PINK1 and PARKIN under baseline and H/R conditions in the *DIAPH1*-silenced vs. shScr cells, and significantly lower expression of the ER stress marker GADD34 under H/R only in the *DIAPH1*-silenced cells (Supplementary Fig. 7a). Expression of other proteins, TOMM40, BCL2, NEFL, PERK, EDEM1, and mitochondrial fission–fusion proteins DRP1 and MFN2 did not significantly differ among these four experimental conditions (Supplement Fig. 7b, c). Collectively, these data suggest key roles for DIAPH1 in the regulation of the protein expression of NRF2, PARKIN, PINK1, and GADD34 at baseline and particularly during H/R conditions.

### Silencing *DIAPH1* in HiPSC-CMs improves mitochondria and SR/ER properties

**Fig. 4:**
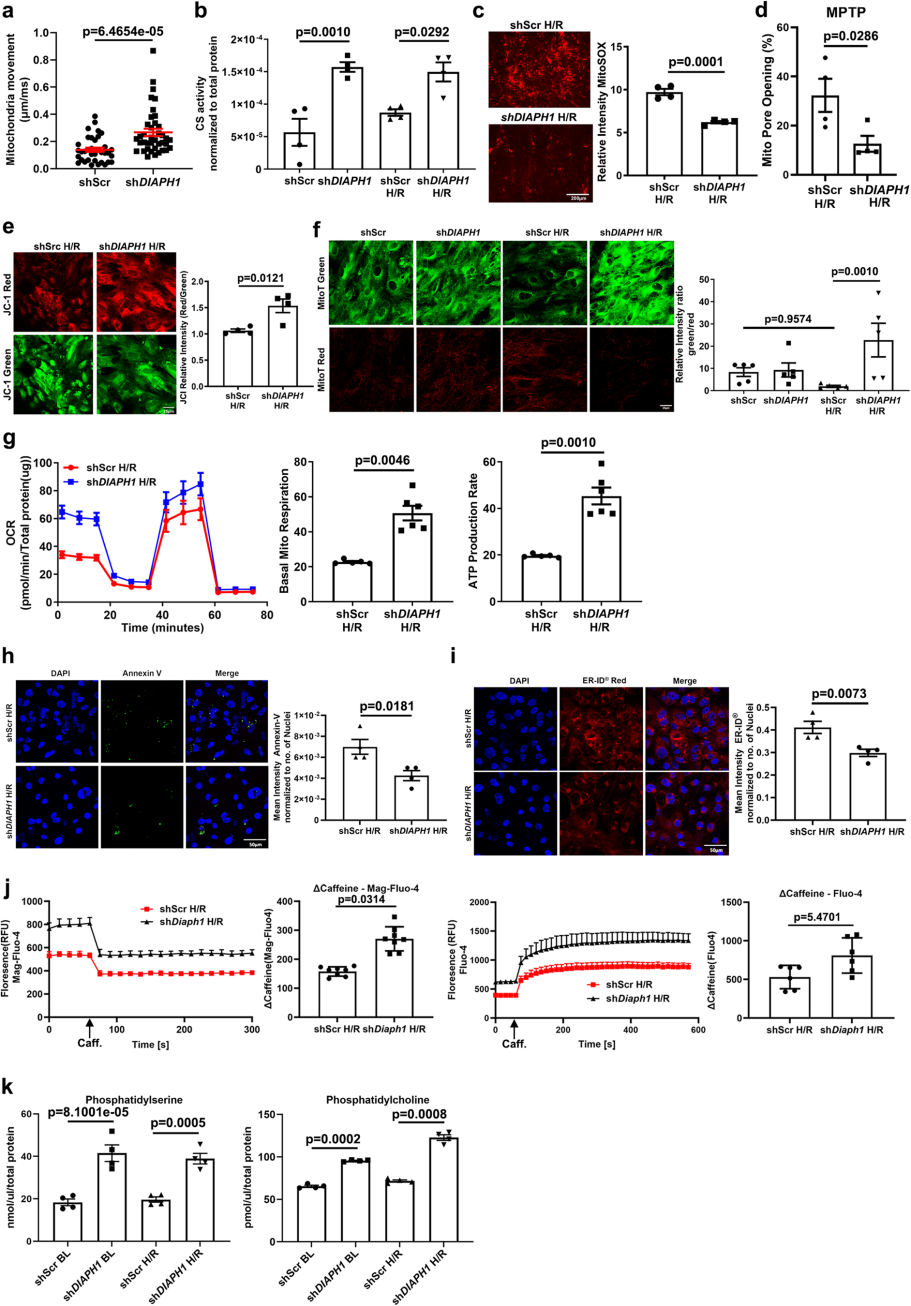
Silencing *DIAPH1* improves mitochondrial and ER function. **a** Represents quantification of mitochondrial velocity (µm/ms) in shScr and sh*DIAPH1* hiPSC-CMs under baseline conditions. Nikon Eclipse Ti Epifluorescence Microscope (Inverted) at 40× magnification was used to obtain images of cells stained with MitoTracker™ Red CMXRos live cell imaging dye (each value represents velocity for individual mitochondria tracked, *n* = 5 biologically independent samples, *p* value obtained from Wilcoxon rank-sum test). **b** Colorimetric assessment of citrate synthase activity in HiPSC-CMs under Baseline and H/R (*n* = 4 biologically independent samples, *p* value obtained from ANOVA with TukeyHSD pairwise comparison test). **c** Mitochondria superoxide measurements as measured by 5 µM MitoSOX dye in shScr and sh*DIAPH1* hiPSC-CMs upon H/R. Scale bar 200 µm (*n* = 4 biologically independent samples, unpaired *t* test was performed for *p* value). **d** Mitochondria pore opening measured by flow-cytometry using mitochondrial Permeability Transition Pore Assay Kit in HiPSC-CMs under H/R conditions (*n* = 4 biologically independent samples, one-tailed Wilcoxon rank-sum test was performed for *p* value). **e** Leica SP8 Confocal microscopy at 63× magnification and respective quantification of the relative intensity of JC-1 (red/green) dye to measure mitochondria membrane potential in shScr and sh*DIAPH1* hiPSC-CMs under H/R (*n* = 4 biologically independent samples, unpaired *t* test was performed for *p* value). **f** Leica SP8 Confocal microscopy images of fluorescent MitoTimer signal of young (green) and old (red fluorescence) mitochondria in HiPSC-CMs. Quantification represents the ratio of the relative intensity of green to red indicating the turnover of Mito (*n* = 4 biologically independent samples, Kruskal–Wallis with Dunn’s pairwise comparison test was performed for *p* value). **g** Represents oxygen consumption rate (OCR) measurements obtained from Seahorse. The panel also represents basal mitochondrial respiration and ATP production rate extrapolated from OCR measurement (*n* = 5 for shScr and 6 for *shDIAPH1* biologically independent samples, Welch’s unpaired *t*-test was performed for *p* value). **h** Leica SP8 confocal images and respective quantification of Annexin V-FITC staining to measure apoptosis in shScr and sh*DIAPH1* HiPSC-CMs exposed to H/R. Scale bar 50 µM. (*n* = 4 biologically independent samples, unpaired *t*-test was performed for *p* value). **i** Leica SP8 confocal microscopy images and respective quantification of ER-ID® Red staining to measure ER stress in shScr and sh*DIAPH1* HiPSC-CMs exposed to H/R conditions. Scale bar 50 µM. (*n* = 4 biologically independent samples, ANOVA with TukeyHSD test was performed for *p* value). **j** Live cell ER lumen and cytosolic calcium measurements in shScr and sh*Diaph1* H9C2 cells exposed to H/R using 20 µM Mag-Fluo-4 AM and 5 µM Fluo-4 AM dye, respectively. ER, calcium content was estimated as the delta between basal and caffeine-stimulated fluorescence measurements (*n* = 6 biologically independent samples for fluo4 and *n* = 8 for Mag-fluo4, unpaired *t*-test was performed for *p* value). **k** Fluorometric assay to measure phosphatidylserine and phosphatidylcholine levels using TECAN infinity pro 200 plate reader (*n* = 4 biologically independent samples, Welch’s ANOVA with Games–Howell pairwise comparison test for phosphatidylcholine and ANOVA with TukeyHSD pairwise comparison for phosphatidylserine was performed for *p* value). Data are presented as the mean ± SEM. A normality test was performed for all groups. All statistics and source data are provided as a Source Data file.

Findings from the RNA-Seq and subsequent validation studies in HiPSC-CMs suggested the potential influence of DIAPH1 in regulating pathways influencing mitochondrial biogenesis, mobility, membrane potential, permeability transition pore (MPT) opening, and generation of ROS. Hence, we probed if silencing *DIAPH1* impacts mitochondria mobility in CMs. In HiPSC-CMs, significantly higher mitochondria movement was observed by confocal microscopy upon silencing of *DIAPH1* vs. Scr controls; *p* < 0.001 (Fig. 4a). However, we did not observe any specific directionality in the mitochondria movement, it was random, that is, in all directions. Citrate synthase (CS) activity, a marker for the presence and content of intact mitochondria and mitochondrial biogenesis, was significantly higher in sh*DIAPH1* vs. shScr CMs under baseline and H/R conditions; *p* < 0.001 and *p* < 0.05, respectively (Fig. 4b). In sh*DIAPH1* HiPSC-CMs vs. shScr HiPSC-CMs, significantly lower mitochondrial superoxide was observed; *p* < 0.001 (Fig. 4c), along with attenuated MPT pore opening; *p* < 0.05 (Fig. 4d) and improved membrane potential (Fig. 4e); *p* < 0.05.

In order to monitor mitochondrial turnover in CMs, we generated *DIAPH1*-silenced vs. shScr HiPSC-CMs expressing the MitoTimer construct integrating MitoTimer and TET-ON inducible rtTA3 constructs using pCW vector for higher transfection efficiency (Supplementary Fig. 8). MitoTimer is a mutant of DsRed fluorescent protein characterized by transition from green fluorescence to red conformation upon maturation and aging of mitochondria under physiological conditions^39^. After successful transfection and activation of the MitoTimer construct with doxycycline for 48 h, HiPSC-CMs were subjected to baseline or H/R. Compared with baseline, induction of H/R results in significantly lower fluorescence green to red ratio in the shScr HiPSC-CMs, indicative of more aged mitochondria and less turnover in H/R; *p* < 0.05 (Fig. 4f). Whereas there were no significant differences in the baseline state between sh*DIAPH1* vs. shScr HiPSC-CMs, (Fig. 4f), higher fluorescence green to red ratio was noted in sh*DIAPH1* vs. shScr group under H/R, indicative of lower content of aged mitochondria with greater turnover in the *DIAPH1-*silenced HiPSC-CMs; *p* < 0.05 (Fig. 4f).

In order to test these concepts in vivo, we intercrossed mice expressing the MitoTimer protein under the control of the cardiac α-MHC promoter^39–41^ with mice globally devoid of *Diaph*1. In the isolated perfused hearts from *Diaph1* null α-MHC MitoTimer mice, a significantly higher MitoTimer fluorescence was demonstrated, as revealed by increased Green:Red ratio when compared to control mice expressing *Diaph1*, both at the baseline and after 30 min of global ischemia followed by 60 min of reperfusion; *p* < 0.001 and *p* < 0.01, respectively (Supplementary Fig. 9). These data indicate that deletion of *Diaph1* in mice led to greater mitochondria turnover in homeostatic baseline conditions as well as in I/R.

Furthermore, with respect to mitochondrial metabolism, in H/R, silencing of sh*DIAPH1* vs. shScr HiPSC-CMs resulted in significantly higher basal mitochondrial respiration and ATP production rate; *p* < 0.01 in both cases (Fig. 4g). We next performed Annexin-V staining to detect apoptosis in HiPSC-CMs exposed to H/R and observed significantly lower Annexin-V staining in cells silenced with *DIAPH1* compared to Scr controls; *p* < 0.05 (Fig. 4h). To assess SR function, we stained HiPSC-CMs exposed to HR with ER-ID® Red stain, a known assay to detect ER stress^42^. We observed significantly lower mean intensity of ER-ID® Red stain in sh*DIAPH1* cells vs. Scr controls, suggestive of less SR stress in H/R; *p* < 0.05 (Fig. 4i).

We examined cytosolic and ER calcium in H9C2 cells exposed to H/R using ER lumen-specific Mag-Fluo-4 AM^43^ and cytosolic Fluo-4 AM live cell calcium dyes. We observed higher basal calcium levels in both ER lumen and cytosolic calcium upon silencing *Diaph1* (Fig. 4j). Upon addition of 20 mM caffeine to elicit ER calcium release, we observed a significantly lower Mag-Fluo-4 relative fluorescence intensity and a significantly higher Fluo-4 fluorescence in sh*Diaph1* cells; *p* < 0.001 and *p* < 0.05, respectively, indicative of higher ER calcium levels compared to shScr controls in H/R (Fig. 4j).

Next, we examined lipid biosynthesis, as Mito-SR/ER serves as a platform for lipid biosynthesis, such as phosphatidylserine (PS) and phosphatidylcholine (PC)^44–46^. It has been postulated that an increase or decrease in the area of the Mito-ER contact may be accompanied by a change in the amount of lipids synthesized at the contact area^47^. We found a significantly higher synthesis of PS from serine and consequent conversion to PC in sh*DIAPH1* HiPSC-CMs under baseline and H/R conditions compared to shScr controls; *p* < 0.01 (Fig. 4k). Taken together, these findings demonstrate that silencing *DIAPH1* favorably influences multiple mitochondria and SR/ER properties, mitochondria turnover in I/R hearts and H/R-treated HiPSC-CMs.

### Deletion of *Diaph1* in CMs protects hearts from ischemic injury

**Fig. 5:**
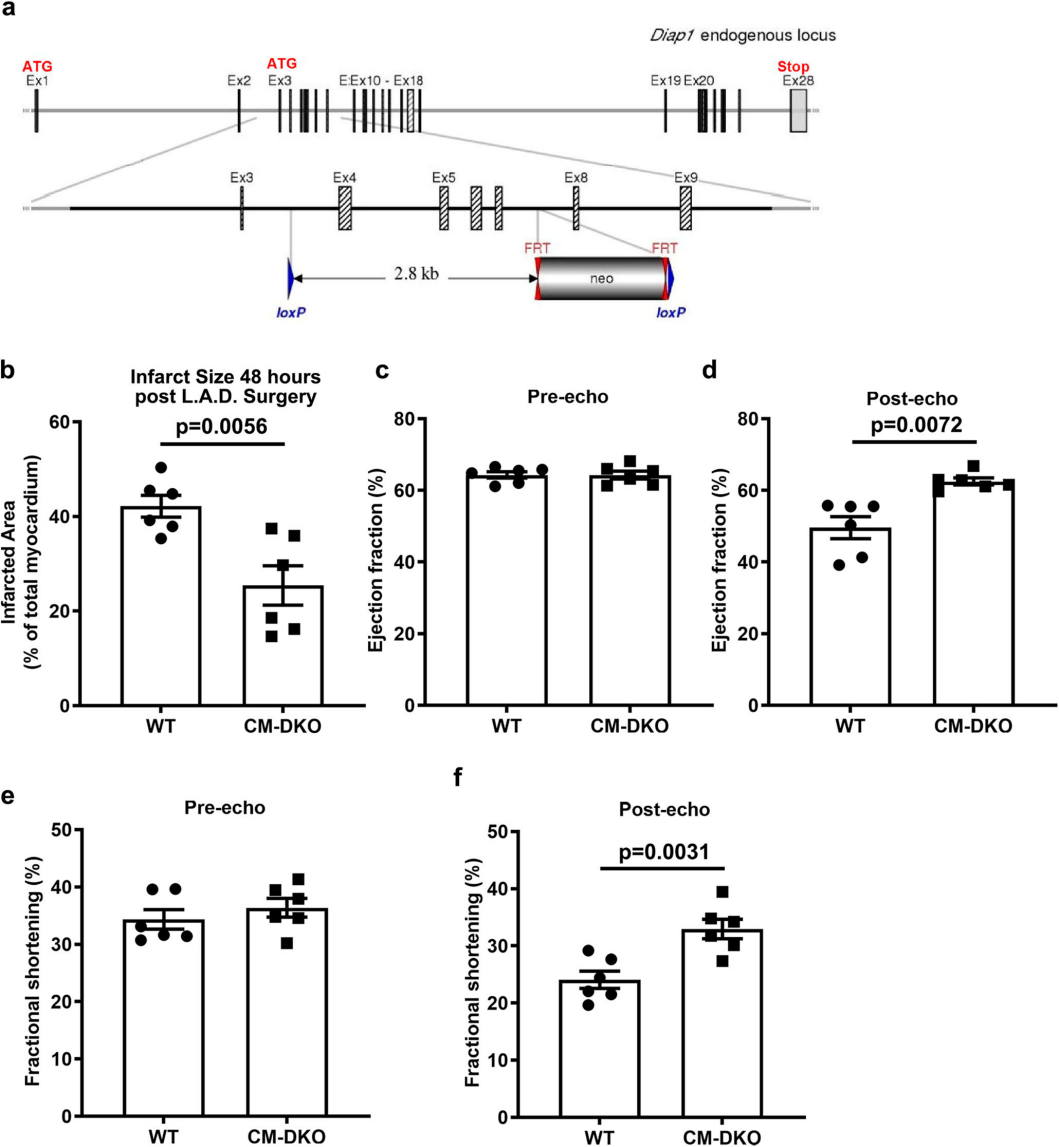
Deletion of CM-specific *Diaph1* is protective in ischemia/reperfusion injury. **a** Generation of *Diaph1* floxed mice. Schematic representation of *Diaph1* targeting strategy, resulting in deletion of exons 4–7. The diagram is not depicted to scale. Hatched rectangles represent *Diaph1* coding sequences, gray rectangles indicate non-coding exon portions, and solid line represents chromosome sequence. The initiation codon (ATG) and stop codon (Stop) are indicated. FRT sites are represented by double red triangles, and loxP sites by blue triangles. *Diaph1*^*flox/flox*^ *Cre* (+) (“CM-DKO”) and *Diaph1*^*flox/flox*^ *Cre* (−) (“WT”) mice hearts which underwent LAD ligation for 30 min creating ischemic/no flow condition followed by reperfusion to mimic I/R injury. **b** Quantification of infarct size in CM-DKO and WT hearts (*n* = 6 biologically independent samples, unpaired *t*-test was performed for *p* value). 2D-echocardiography measurements of (**c**, **d**), ejection fraction (*n* = 6 biologically independent samples, unpaired *t*-test was performed for *p* value) and **e**, **f** fractional shortening 48 h post LAD surgery in CM-DKO and WT mice (*n* = 6 biologically independent samples, Wilcoxon rank-sum test was performed for pre-echo and unpaired *t*-test was performed for post-echo *p* value). Data are presented as the mean ± SEM. All statistics and source data are provided as a Source Data file.

These findings suggest that DIAPH1 plays a key role in the CM response to I/R. To directly address this point, we generated *Diaph1* floxed mice (Fig. 5a, Supplementary Fig. 10a). *Diaph1*^*flox/flox*^ mice were bred with Myosin Heavy Chain (*MHC*) *Cre*-recombinase mice to specifically delete *Diaph1* in CMs; this strategy yielded *Diaph1*^*flox/flox*^ *MHC Cre* (+) (CM-specific *Diaph1-*deleted mice) and littermate *Diaph1*^*flox/flox*^ *MHC Cre* (−) (*Diaph1* wild-type (WT)) mice). This resulted in the deletion of *Diaph1* from CMs; *p* < 0.001, but not from the kidney (Supplementary Fig. 10b). Hereafter, these animals will be referred to as *Diaph1*^*flox/flox*^ *Cre* (+) and *Diaph1*^*flox/flox*^ *Cre* (−) mice. To determine potential roles for DIAPH1 in the heart after I/R, we subjected *Diaph1*^*flox/flox*^ *Cre* (+) (CM-DKO in the figures) and *Diaph1*^*flox/flox*^ *Cre* (−) (WT in the figures) mice to ligation and reperfusion of the left anterior descending coronary artery (LAD). Infarct size was significantly lower in *Diaph1*^*flox/flox*^ *Cre* (+) mice vs*. Diaph1*^*flox/flox*^ *Cre* (−) at 48 h; *p* < 0.01 (Fig. 5b and Supplementary Fig. 10c). Although there were no genotype-dependent differences in pre-I/R ejection fraction or % fractional shortening by echocardiography **(**Fig. 5c, e), at 48 h post I/R, CM-specific *Diaph1*-deleted mice displayed a significantly higher ejection fraction and % fractional shortening; *p* < 0.01 (Fig. 5d, f), relative to *Diaph1*^*flox/flox*^ *Cre* (−) mice. These findings indicated that the expression of DIAPH1 in CMs is a key mediator of I/R injury in murine hearts.

### Binding of DIAPH1–RAGE interactions in regulation of DIAPH1–MFN2 interactions and mitochondria–SR/ER distance

**Fig. 6:**
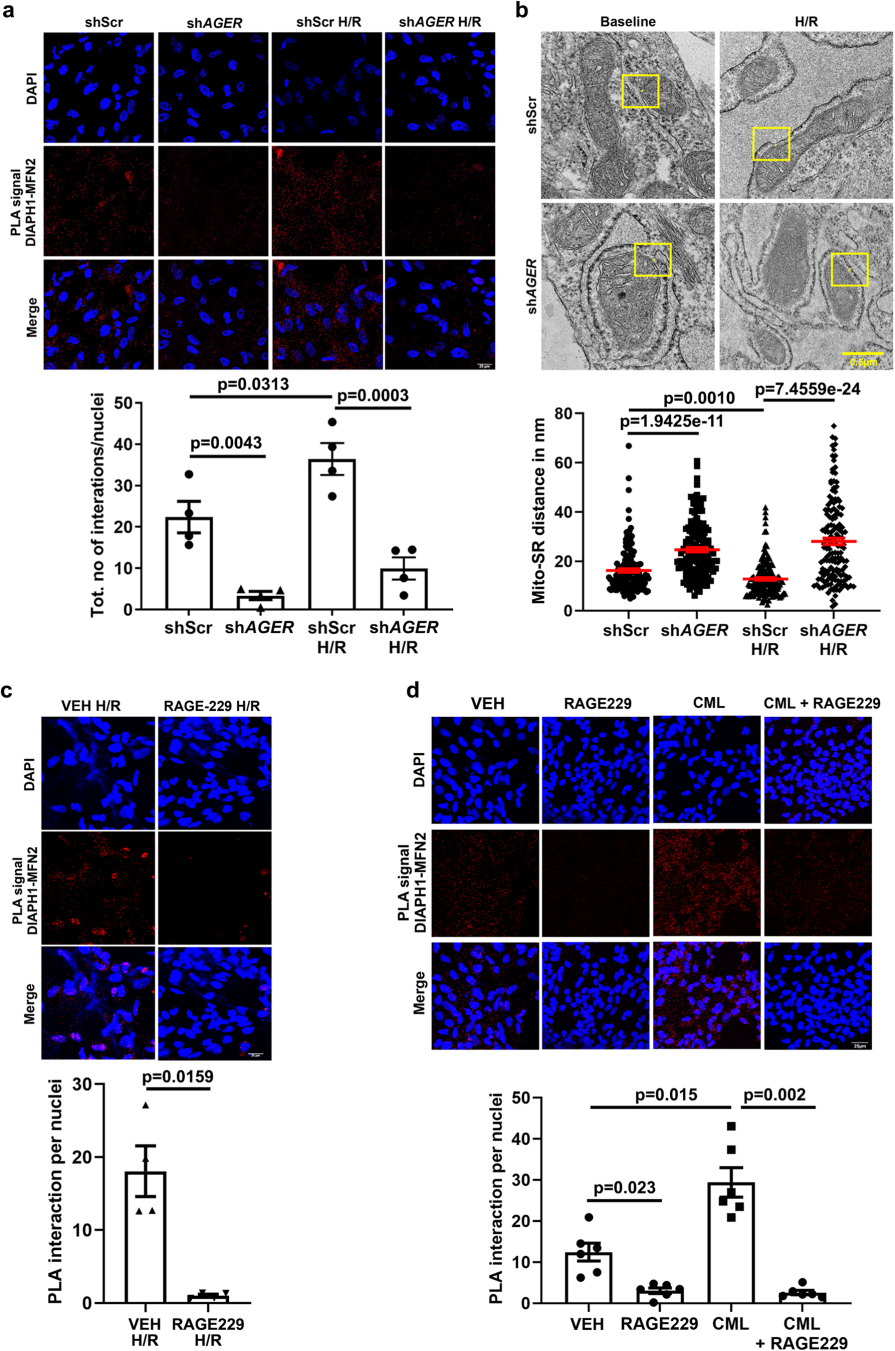
RAGE–DIAPH1 regulates Mito-SR/ER distance and DIAPH1–MFN2 interactions in HiPSC-CMs. **a**. Leica SP8 confocal microscopy images at 63X magnification of DIAPH1–MFN2 DUOLINK PLA signal and corresponding quantification of signal in sh*AGER* and shScr HiPSC-CMs under baseline and H/R conditions. Scale bar 25 µm. (*n* = 4 biologically independent samples, ANOVA with TukeyHSD pairwise comparison test was performed for *p* value). **b** Represents TEM images to measure Mito-SR/ER distance and quantification using NIH-ImageJ software in sh*AGER* and shScr HiPSC-CMs under baseline and H/R conditions. Scale bar 0.5 µm. (Each value represents Mito-SR/ER distance in nm originating from four biologically independent samples, Kruskal–Wallis with Dunn’s pairwise comparison test was performed for *p* values) **c** Leica SP8 confocal microscopy images at 63× magnification of DIAPH1–MFN2 DUOLINK PLA signal and corresponding quantification in HiPSC-CMs treated with RAGE229, 10 µM, for 1 h under baseline and H/R conditions. Scale bar 25 µm. (*n* = 4 biologically independent samples, unpaired *t*-test was performed for *p* value). **d** Treatment with CML-AGE, 500 µg/ml, for 1 h and RAGE229, 10 µM, for 1 h. For combined treatment, RAGE229 was added 10 min prior to the addition of CML-AGE treatment for 1 h. Scale bar 25 µm. (*n* = 6 biologically independent samples, Welch’s ANOVA with Games–Howell pairwise comparison test was performed for *p* value). Data are presented as the mean ± SEM. All statistics and source data are provided as a Source Data file.

We previously demonstrated that DIAPH1 interacts with the cytoplasmic tail (ct) of the receptor for advanced glycation end products (RAGE; gene symbol *Ager*)^48^; RAGE is expressed in HiPSC-CMs and DIAPH1 is important for RAGE signaling. In H/R, we observed a significant increase in DIAPH1–MFN2 PLA interaction in shScr HiPSC-CMs; *p* < 0.05, which was significantly reduced by silencing *AGER* vs. shScr in baseline and H/R conditions; *p* < 0.01 and *p* < 0.001, respectively (Fig. 6a). Hence, these findings led us to probe if RAGE and its ligands influence Mito-SR/ER distance and DIAPH1–MFN2 interactions. TEM images revealed that silencing *AGER* increased the Mito-SR/ER distance in HiPSC-CMs in the baseline condition to 24.72 nm and in H/R to 28.12 nm compared to shScr, in which distances of 16.27 nm and 12.85 nm respectively, were observed; *p* < 0.001 (Fig. 6b).

Treatment of HiPSC-CMs with the small molecule antagonist of RAGE–DIAPH1 interaction, RAGE229^49^, reduced DIAPH1–MFN2 interactions by PLA in Hi-PSC-CMs exposed to H/R vs. vehicle (VEH); *p* < 0.05 (Fig. 6c). Furthermore, treatment with RAGE ligand, carboxymethyllysine (CML)-advanced glycation end product (AGE), resulted in a significant increase in DIAPH1–MFN2 interaction by PLA vs. VEH; *p* < 0.001 (Fig. 6d). Treatment with RAGE229 alone or in combination with CML-AGE significantly attenuated DIAPH1–MFN2 interaction by PLA vs. VEH; *p* < 0.01 and *p* < 0.001, respectively (Fig. 6d). These studies revealed that RAGE and its ligands promote DIAPH1–MFN2 interaction and shortening of the Mito-SR/ER distance. Importantly, small molecule antagonism of RAGE–DIAPH1 interaction, using RAGE229, significantly reduced the DIAPH1–MFN2 interactions and increased the Mito-SR/ER distance.

### Mito-SR distance mediates H/R injury in HiPSC-CMs

**Fig. 7:**
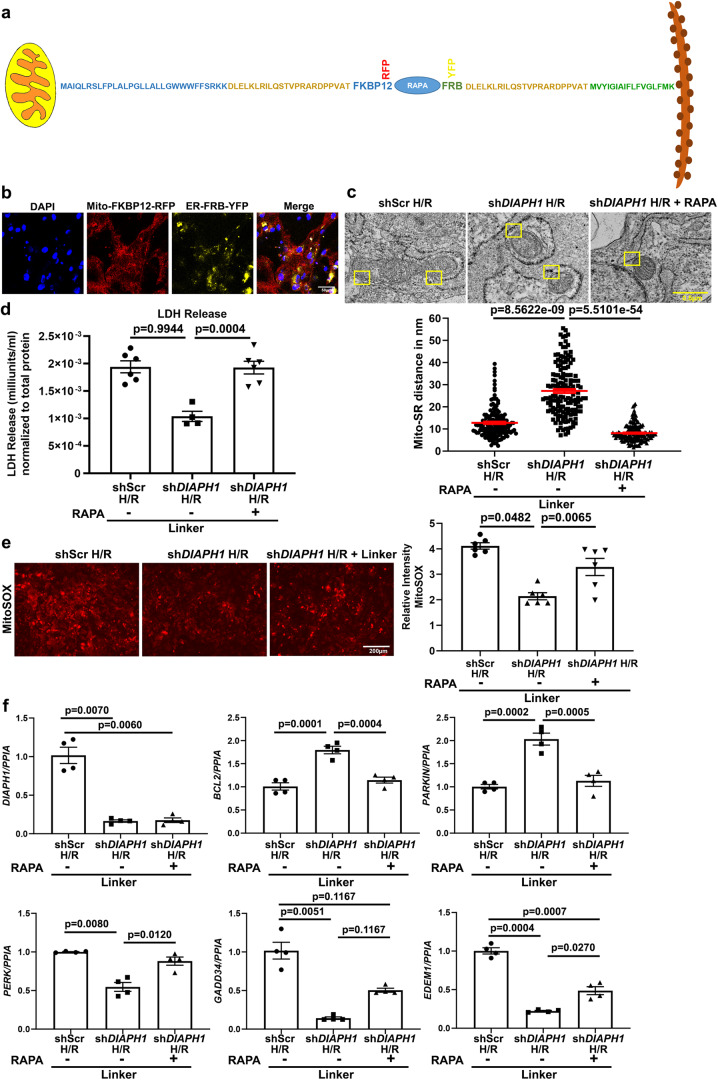
Reduction of Mito-SR/ER distance with physical linkers abrogates the benefits of *DIAPH1* silencing in HiPSC-CMs in H/R. **a** Scheme representing the construction of FRB–FKBP12–RAPAMYCIN–Linker complex fluorescence YFP and RFP tags. **b** Leica SP8 confocal microscopy images at 63× magnification representing 48 h post Lipofectamine LTX transfection followed by treatment with 100 nM rapamycin for 10 min. Scale bar 25 µm. (Data reproduced from two independent experiments) **c** TEM images and quantification after successful transfection with mitochondria and SR/ER localization sequences with or without 100 nM rapamycin treatment to reduce Mitochondria and SR/ER to approximately 5 nm. Scale bar 0.5 µm. (Each value represents Mito-SR/ER distance in nm originating from four biologically independent samples, Kruskal–Wallis with Dunn’s pairwise comparison test was performed for *p* values) **d** Colorimetric assay to detect LDH levels in conditioned medium obtained from linker groups (*n* = 4–6 biologically independent samples, ANOVA with TukeyHSD pairwise comparison test was performed for *p* value). **e** MitoSOX staining to detect mitochondrial superoxide and respective quantification using NIH-ImageJ. Images were taken at 10× magnification using an EVOS epifluorescence microscope with an RFP filter. Scale bar 200 µm. (*n* = 6 biologically independent samples, ANOVA–TukeyHSD pairwise comparison test was performed for *p* value). **f** qPCR under linker conditions for SR/ER and mitochondrial markers. *N* represents biological replicates originating from at least two consecutive batches. (*n* = 4 biologically independent samples. Welch’s ANOVA with Games–Howell pairwise comparison test for *DIAPH1*, *PERK*, and *EDEM1*, Krushal–Wallis with Dunn’s pairwise comparison test for *GADD34*, and ANOVA with TukeyHSD pairwise comparison *for BCL2 and PARKIN* for *p* values). Data are presented as the mean ± SEM. All statistics and source data are provided as a Source Data file.

Finally, it was critical to directly interrogate the relationship between Mito-SR distance and H/R injury and the expression of DIAPH1. To address this point, we employed the physical linker approach to reduce the distance between mitochondria and SR/ER, as previously demonstrated by Csordás and colleagues^50^, in sh*DIAPH1* and shScr HiPSC-CMs, which would be hypothesized to abrogate the protective effects of *DIAPH1* silencing on reduction of H/R injury in *shDIAPH1*-silenced cells. This is exactly what we observed. Figure 7a illustrates the FRB–FKBP12–Rapamycin complex connected by the linker DLELKLRILQSTVPRARDPPVAT employed in these studies. We constructed both SR/ER and mitochondria localization sequences with or without fluorescence tags; in this system, brief treatment with low dose rapamycin (100 nM) for 10 min promotes activation of the linker complex formation to reduce the Mito-SR/ER distance to approximately 5 nm. Confocal microscopy confirmed transfection of both Mito-FKBP12-RFP and SR-FRB-YFP constructs (Fig. 7b). Using TEM imaging, we confirmed reduction of the physical distance from 27.16 to 8.12 nm between mitochondria and SR/ER in linker-transfected and Rapamycin-treated sh*DIAPH1* group under H/R; *p* < 0.001 (Fig. 7c). Note that the linker was transfected into the HiPSC-CMs in each condition; however, only when rapamycin is added (noted as +Rapa in the figure) is the linker active and able to reduce the Mito-SR/ER distance.

Having characterized the linker and its ability to reduce the Mito-SR/ER distance in HiPSC-CMs, we next assessed cellular toxicity and mitochondrial superoxide generation under H/R. As observed above in Figs. 3 and 4, H/R induced significantly higher LDH release and mitochondrial superoxide in shSc*r* HiPSC-CMs compared to sh*DIAPH1* HiPSC-CMs. As illustrated in Fig. 7d, e, reduction of the physical distance between mitochondria and SR/ER prevented the reduction in LDH release and mitochondrial superoxide generation that was observed in sh*DIAPH1* HiPSC-CMs; *p* < 0.001 and *p* < 0.01, respectively, thereby effectively blocking the protective effects of *DIAPH1* silencing on the release of LDH and mitochondrial superoxide generation in H/R. Upon reduction of the physical distance between mitochondrial and SR/ER in HiPSC-CMs subjected to silencing of *DIAPH1* using this linker, we observed significantly lower mRNA expression of *BCL2* and *PARKIN* compared to sh*DIAPH1* HiPSC-CMs bearing the linker but without the addition of Rapamycin; *p* < 0.001 in both cases (Fig. 7f). Furthermore, upon reduction of the physical distance between mitochondria and SR/ER in HiPSC-CMs subjected to silencing of *DIAPH1* using this linker plus Rapamycin, higher mRNA expression of *PERK, GADD34*, and *EDEM1* was observed vs. sh*DIAPH1* HiPSCs-CMs in the presence of the linker but without the addition of Rapamycin; *p* < 0.001, *p* < 0.05, and *p* < 0.01, respectively. Taken together, these studies affirm that DIAPH1-dependent reduction of the mitochondria–SR/ER distance is a key driver of DIAPH1-mediated injury in H/R.

## Discussion

Cellular homeostasis requires effective crosstalk and functional coordination between multiple organelles^50–55^. Inter-organelle communication is accomplished largely through direct physical contact between organellar membranes^13,50–55^. One of the best characterized inter-organellar communication sites is the connection between the mitochondria and the SR/ER. Mitochondria–SR/ER contact sites require proteins on both the SR/ER and mitochondria to bridge the two organelles. While the nature of these protein tethers has not been fully characterized in mammalian cells, several Mito-SR/ER tethering complexes have been identified. Multiple tethers allow the establishment of Mitochondria-SR/ER contacts and include MFN1/MFN2 homodimers and heterodimers, ITPR-GRP75-VDAC complex, FIS1/BAP31, Vesicle-associated membrane protein B (VAPB)/ protein tyrosine phosphatase interacting protein 51 (PTPIP51) and PDZD8^56–60^. Some Mitochondria–ER-associated proteins, including CisD2, PACS2, PDK4, and SIGMAR1, interact with tethers to modulate Mitochondria–ER contacts^8,58–60^. Here we identified that DIAPH1 regulates Mitochondria–SR/ER tethering via its interaction with MFN2. Through DUOLINK PLA assay, immunoprecipitation studies, binding assays, ELISA, cross-linking and mass spectrometry, and multiple NMR spectroscopy-based strategies, we demonstrate for the first time a direct interaction between DIAPH1 and MFN2.

Consistent with findings in DUOLINK PLA (Fig. 1e), purified DIAPH1 binds to the cytosolic fragment of MFN2 with sub-nanomolar affinity at neutral pH, and the affinity increases to picomolar range at acidic pH levels (Fig. 2b). DIAPH1–MFN2 binding disrupts the intramolecular DID-DAD interaction, which is critical for DIAPH1 autoinhibition, thereby resulting in activation of DIAPH1 at the DIAPH1–MFN2 points of contact. Structural studies of the purified DIAPH1–MFN2 complex show that the DIAPH1 dimer binds to two MFN2 molecules so that the distance between two bound MFN2s is about 10 nm (Fig. 2h). Since MFN2 is located at the outer membrane of both mitochondria and SR, the MFN2-MFN2 distance observed in the DIAPH1–MFN2 complex is consistent with the EM measurements of the distances between mitochondria and SR (Fig. 1b). Taken together, structural studies support that DIAPH1 functions as a regulator of mitochondria–ER/SR tether via its interaction with MFN2.

Mitochondria and SR/ER contacts are responsible for a wide range of cellular processes, including mitophagy, autophagy, mitochondrial biogenesis, ER/SR stress responses, oxidative stress, lipid exchange, calcium signaling, intracellular trafficking, and immune responses. Here, we demonstrate that deletion or silencing of *DIAPH1* or *AGER* increases the distances between mitochondria and SR/ER and that *DIAPH1* silencing in HiPSC-CMs facilitates mitochondria biogenesis, enhances mitophagy, mitochondrial membrane potential, lipid synthesis that occurs at Mitochondria-ER/SR contact area, enhanced caffeine-induced calcium release from ER/SR, and aids in mitochondria turnover (Fig. 4). However, shortening these distances in HiPSC-CMs, using linker constructs activated by low dose rapamycin, reverses the beneficial effects of reducing *DIAPH1* expression. In contrast, small molecule antagonism of RAGE–DIAPH1 interaction using RAGE229 reduced DIAPH1–MFN2 interaction and increased the mitochondria–SR/ER distance.

Our studies suggest that DIAPH1 deletion highlighted two contrasting phenotypes, mitochondria biogenesis and mitophagy. In HiPSC-CMs subjected to *DIAPH1* silencing, the protein expression of key mitophagy markers PARKIN and PINK1 was highly upregulated along with the upregulation of NRF2 required for biogenesis. Our MitoTimer studies provide insight into how these two contrasting mitochondria processes may be balanced in *DIAPH1*-silenced HiPSC-CMs. In this regard, Gottlieb and colleagues^61^ demonstrated that the red mitochondria detected in the MitoTimer technique were decorated with LC3, supporting the notion that these senescent mitochondria were targeted for autophagic removal. In contrast, the green mitochondria were indicative of the young mitochondria engaged in mitochondrial biogenesis. As previously discussed by Gottlieb and colleagues^61^, and as suggested by our MitoTimer data (Fig. 4), a dynamic balance between mitophagy and mitochondrial biogenesis is required for superior mitochondria function. Our data demonstrate this phenomenon and hence the protection rendered by silencing *DIAPH1* under stress conditions.

Our studies suggest that disruption of mitochondria–SR/ER tethering is a protective mechanism under H/R and I/R in CMs and hearts, respectively. These findings are consistent with two earlier studies that demonstrated protection from H/R and I/R through modulation of the Mitochondria–ER tethering/contacts. First, studies by Ovize et al.^1^ showed protection from H/R by modulation of SR/ER-mitochondria Ca^2+^ crosstalk via the VDAC1/Grp75/IP3R1 complex^1^. Second, studies in MFN2-MFN1 double null mice hearts showed that disruption of mitochondria–SR/ER tethering resulted in a reduction in ischemic injury and attenuation of ROS^62^. While our overall findings on protection from I/R and H/R injury in *DIAPH1*-silenced hearts and *DIAPH1*-silenced HiPSC-CMs are similar to the protection observed in MFN2–MFN1 knockdown conditions, including rescued mitochondria pore opening, there are distinct differences between *DIAPH1* deletion vs*. MFN2* deletion. First, in the HiPSC-CMs, *DIAPH1* deletion and silencing did not reduce *MFN2* mRNA expression. Second, unlike in *MFN2* deletion^62^, silencing of *DIAPH1* also resulted in increased mitochondria respiration and enhanced mitochondrial biogenesis. Third, while silencing of *MFN2* impaired mitophagy and was linked to changes in phosphorylation of MFN2 by PINK1 kinase^9,62^, *DIAPH1* silencing increased mitophagy and mitochondria turnover. Hence, *DIAPH1* silencing fosters MFN2-dependent and MFN2-independent mechanisms by which it attenuates mitochondria stress and protects from I/R and H/R injury.

These considerations underscore that in contrast to targeting MFN2 to modulate tethering, a translational focus on DIAPH1 that results in increasing mitochondria–SR/ER distance to augment mitochondrial functions and reduce I/R injuries may be a superior approach. This premise is further buttressed by our findings that RAGE ligands enhance DIAPH1-MFN2 interactions and that silencing of *AGER* and/or small molecule antagonists of RAGE–DIAPH1 interaction reduces DIAPH1-MFN2 interactions and increases mitochondria–SR/ER distance. Earlier studies demonstrated that the cytoplasmic domain of RAGE binds to DIAPH1^48^ through the latter’s FH1 domain^48,63,64^. Previous work reported that ligand-induced association of RAGE homodimers on the cell surface increases the molecular dimension of RAGE, thereby recruiting DIAPH1 and facilitating downstream signal transduction pathways^63^. Here, we demonstrated that DIAPH1 utilizes its released inhibitory domains to bind two MFN2 molecules located on the mitochondria and SR/ER to facilitate tethering of these organelles; these interactions are specific and susceptible to therapeutic approaches to reduce the detrimental effects evoked by I/R injury in the heart.

Hence, we propose that unlike direct manipulation of MFN2 in which metabolic dysfunction may be triggered as evidenced by studies reporting the effects of *Mfn2* deletion, by contrast, deletion/silencing (in CMs) or antagonism of DIAPH1 reduces cardiac I/R-mediated infarct size; prevents loss of cardiac function; and increases levels of cardiac ATP^18,49^. Collectively, the present studies unveil a pivotal mechanism by which mitochondria-ER communications and their critical impact on metabolism and cellular fate and survival are regulated through extracellular cues triggered by the accumulation of RAGE ligands, which, through DIAPH1 binding to MFN2, engage the mitochondria-SR/ER interface and are susceptible to interventions to protect the heart in I/R.

## Methods

All animal procedures were approved by the Institutional Animal Care and Use Committee at New York University Grossman School of Medicine. All mice studied were male and in the C57BL/6 J background, had free access to water and food, and were subjected to 12-hour light/dark cycles.

### *Diaph1* floxed mice

Based on our bioinformatics analysis of the mouse Diaph1 cDNA sequence (NM_007858) and the exon / intron organization of the gene, which has 28 exons separated by 27 introns, we developed a floxed mouse line suitable for the generation of a conditional deletion model for the Diaph1 gene. As illustrated in Fig. 5a in the manuscript, we designed a targeting strategy leading to the insertion of a loxP site together with an FRT-flanked neomycin selection cassette within the intron 7 and a single distal loxP within the intron 3 of Diaph1. This was followed by homologous recombination in embryonic stem (ES) cells, blastocyst injection, generation and identification of chimeras, identification of chimeric mice, and breeding with Flp deleter mice to remove the neomycin cassette. As shown in Supplementary Figure 10a, we validated the generation of the floxed *Diaph1* mice by Flp-excised PCR using the primer pair 35548flp-AMS1 and 35549-AMS1. The excision status of *Diaph1* allele was analyzed based on the PCR amplification product of different sizes: 0.5, 0.4, and 2.2 kb corresponding to the Flp-mediated Neo-excised allele, the wild-type allele and the recombined (non-excised) allele respectively.

### Ex vivo ischemia/reperfusion (I/R)

Male mice, aged approximately 3–5 months, were anesthetized with an intraperitoneal injection of ketamine and xylazine (120 mg/kg body weight and 5 mg/kg body weight, respectively), and after deep anesthesia was achieved and documented, hearts were rapidly excised and retrograde perfused (Harvard Apparatus) at 37 °C in a nonrecirculating mode through the aorta as published^18,65^. The hearts were perfused at a constant pressure at a flow rate of 2.5 mL/min with modified oxygenated Krebs–Henseleit buffer containing 118 mmol/l NaCl, 4.7 mmol/l KCl, 2.5 mmol/l CaCl_2_, 1.2 mmol/l MgCl_2_, 25 mmol/l NaHCO_3_, 5 mmol/l glucose, 0.4 mmol/l palmitate, 0.4 mmol/l BSA, and 70 mU/l insulin^18,65^. After the hearts were allowed to equilibrate for 30 mins (baseline), they were subjected to 30 min of global (zero-flow) ischemia and 60 min of reperfusion with modified Krebs–Henseleit buffer.

### In vivo ischemia/reperfusion

Male mice, aged approximately 3–5 months, were anesthetized with an intraperitoneal injection of ketamine and xylazine (120 mg/kg body weight and 5 mg/kg body weight, respectively) and subjected to left anterior descending coronary artery occlusion followed by reperfusion (LAD/reperfusion), as published earlier^18,66^. The surgical procedures were performed by an operator naive to the mouse genotype. Briefly, the LAD was ligated for 30 min, and then blood flow was restored and the mice were monitored and sacrificed 48 h after the procedure.

### Measurement of infarct area

At the end of ex vivo and in Vivo LAD/reperfusion studies, mice hearts were used to assess the area at risk and infarct area by 2,3,5-triphenyl-2H-tetrazolium chloride (TTC) and Evan’s blue staining, as published^18,66^. Heart sections were then placed into a pre-warmed 1% 2,3,5-Triphenyltetrazolium chloride (TTC) solution and kept at 37 °C for 10 min. The slices were removed from the solution and photographed. The images were analyzed using AxioVision software.

### Echocardiography measurements

Echocardiography was performed using a Vevo 2100 high-resolution ultrasound imaging system (Visual Sonics Inc. Toronto, Ontario, Canada). Mice were anesthetized using 2% isoflurane, inhaled via a nose cone, and placed on a temperature-maintained platform in a supine position. Core body temperature was monitored using a rectal probe and maintained at 37 °C with an infrared heat lamp throughout the procedure. Hair was removed from the chest using Nair hair remover. Warmed ultrasound transmission gel was placed on the chest, and left parasternal long-axis view and basal short-axis views were acquired. Stroke volume, cardiac output, and ejection fraction were evaluated on the left parasternal long axis view, while fractional shortening was calculated from the short axis view using Vevo 2100 software.

### Human heart samples

De-identified formalin-fixed representative sections of the archival blocks from the hearts of patients with end-stage ischemic heart failure were obtained at the time of heart transplantation from the NYU Langone Health pathology laboratory under protocol # s20-01151 approved by the Institutional Review Board at New York University Grossman School of Medicine. Sections from archival tissue blocks from patients’ post-transplant biopsies with no evidence of rejection or ischemia and normal ejection fraction were used as normal controls.

### Cell culture

#### HiPSCs and HiPSC-CMs

Human-induced pluripotent stem cell (HiPSC) line NCRM1 was obtained from the NIH regenerative medicine program. HiPSC-derived CMs (HiPSC-CMs) were obtained by differentiating HiPSCs. Human adult dermal fibroblasts (cat# 10HU-014) and fibroblast growth medium (cat # MD-0011) were purchased from iXCells Biotechnologies. Cells were initially cultured in a growth medium containing 10% FBS and 1% penicillin/streptomycin until fully confluent. Reprogramming was performed using an RNA-based approach using ReproRNA™-OKSGM kit (cat # 05930) from STEMCEL^TM^ technologies. In brief, the protocol was performed as per the manufacturer’s instructions. At approximately day 35–40, under sterile aseptic conditions, isolated iPSC colonies were cut using a needle and picked into separate wells of 6-well plates coated with Matrigel. HiPSCs were further maintained up to 6 passages to obtain pure colonies with clear edges. HiPSCs were further validated by immunofluorescence staining for iPSC markers using antibodies to OCT4, SOX2, and NANOG. Early passage cells were used for all studies. HiPSCs were cultured on plates coated with hESC-qualified Matrigel® matrix (Corning, cat# 354277) and maintained in mTeSR^TM^1 maintenance medium (STEMCEL^TM^ technologies cat# 85850). Differentiation of CMs was performed using STEMdiff™ Cardiomyocyte Differentiation Kit (STEMCEL^TM^ technologies cat# 05010). Upon 100% confluency of hiPSCs, considered as day 0, differentiation was initiated according to the manufacturer’s guidelines. All experiments were performed between days 25–35 after successful differentiation.

#### H9C2 cell line

Rat myoblast cell lines derived from embryonic BD1X rat heart tissue were obtained from ATCC (cat# CRL-1446). In brief, cells were cultured in DMEM 5 mM glucose (ThermoFisher) with 10% FBS and 1% penicillin/streptomycin. All experiments were performed in passages (P) 6–10.

#### HMVEC-Cs

HMVECs of cardiac origin (HMVEC-C) were obtained from Lonza (cat# CC-7030). ECs were cultured and maintained in an EBM-2 basal medium supplemented with EGM-2 MV SingleQuots™ Kit (cat# CC-3156/CC-4147). Experiments were performed at P6, considering primary ECs may become senescent at passages P8–10^67^.

#### Bone marrow-derived macrophages

Mouse BMDMs were obtained by isolating, culturing, and differentiating cells into macrophages from femora and isolated bone marrow from WT and mice globally devoid of *Diaph1* as described before^68^.

#### Gene silencing studies in vitro

Short-hairpin(sh) approach using lentiviral particles was used for studies to achieve the silencing of genes of interest. Silencing *DIAPH1* and *AGER* in HiPSC-CMs and *DIAPH1* in H9C2 and HMVECs was achieved using mission shRNA custom lentiviral particles from Millipore Sigma (Supplementary Table 8). Short-hairpin targeting scrambled (Scr) sequence was used as a control for studies. For all cell types, transduction was performed at a confluency of 30–40%. In total, 1.5 × 10^6^ viral particles of either Scr or gene of interest at an approximate multiplicity of infection (MOI) of 3 were used for transduction. Hexadimethrine bromide (Sigma cat# 107689) at a concentration of 8 µg/ml was used to increase lentiviral uptake and transduction efficiency. Cells were incubated for 5–6 h with Lentiviral particles to obtain excellent transduction efficiency, and thereafter, cultures were replaced with regular growth medium. Forty-eight hours post transduction, cells were selected against puromycin resistance (Sigma cat# P8833) at a concentration of 2 ng/ml for successful transduction. In the case of HiPSC-CMs, due to poor transduction efficiency, precursor HiPSCs were initially transduced and tested for silencing efficiency by qPCR. Upon achieving successful transduction, the cells were differentiated into HiPSC-CMs for experiments.

#### Induction of hypoxia and reoxygenation

Exposure of cells to hypoxia and reoxygenation (H/R) was achieved using BioSpherix hypoxia chamber. In studies in which H/R was performed, cells were exposed to 0.1% oxygen for 30 min followed by 1 h reoxygenation, 21% oxygen.

#### LDH measurement

LDH release was measured in the conditioned medium obtained from HiPSC-CMs exposed to H/R. The levels of LDH were assayed using commercially available kits from Sigma Aldrich (cat # MAK066) according to the manufacturer’s instructions. Absorbance at 450 nm was measured using TECAN Infinity Pro 200 plate readers. Data were normalized to total protein concentration, as measured by BCA assay.

#### Flow cytometry

shScr and sh*DIAPH1* HiPSC-CMs were trypsinized, passed through 100 µm filters, and re-suspended in cell sorting buffer (50 mM Tris pH 7.5, 0.5 mM EDTA, and 1% BSA). Cells were incubated with Fc Block (Biolegend) for 30 mins at 4 °C before staining with fluorescence-labeled primary antibodies and washed with sorting buffer prior to analysis. The antibodies used were anti-rabbit TNNT2-FITC (ABCAM cat # ab105439) and anti-rabbit TNNI3 (US Biological, cat # 043107). Cells were analyzed using an LSR II analyzer (BD Bioscience) available at NYU core facility, processed by FACS DIVA, and analyzed using FlowJo software.

#### RNAseq studies

High-quality RNA devoid of genomic DNA from shScr and sh*DIAPH1* silenced HiPSC-CMs was obtained using RNeasy Plus Mini Kit (QIAGEN, cat#74134). cDNA synthesis was performed using qScript™ cDNA SuperMix (Quanta BioSciences, Inc. cat # 95048). Before proceeding to RNAseq, samples were analyzed using Bioanalyzer High Sensitivity RNA Analysis. All samples used in the studies had a minimum RNA Integrity number (RIN) value of 9.5 or above. Raw 52-bp paired-end Illumina read data were processed following standard quality control practices to remove low-quality reads and adapter sequence contamination^69–71^. Remaining high-quality trimmed read pairs were aligned to the human genome (hg19) using STAR 2.7.3a, and read pair counts per gene were summed with the feature Counts function in subread 1.6.3 using the GENCODE 34lift37 annotation release to obtain a gene expression matrix^72–74^. Technical replicates across two Illumina sequencing batches were summed.

Differential gene expression among *DIAPH1* silencing and Scr was tested under baseline conditions and under hypoxia using limma with TMM normalization and voom transformation^75,76^. We modeled “group”, defined by combinations of knockdown status and hypoxia status, as a fixed effect (~0+ group) and defined contrasts to test differential expression between *DIAPH1* silencing and Scr in each treatment condition and between hypoxia treatments in each silencing status. We also tested differential expression at the level of KEGG, REACTOME, and GO gene sets using ROAST and CAMERA^77,78^. False-discovery rate (FDR) < 0.05 was applied throughout^79^.

#### Nanostring

Multiplex detection of gene expression was performed using nCounter technology by NanoString. Customized gene code sets including housekeeping human genes related to Mitochondria and ER function, were identified and short-listed for multiplex detection. RNA free of genomic contaminants was extracted, as noted above. Further processing and running of code sets was performed by the Gene Technology Transfer Core at NYU Grossman School of Medicine as per the manufacturer’s guidelines.

#### Quantitative PCR

RNA for both cells and animal tissue was extracted using RNeasy Plus Mini Kit (QIAGEN, cat#74134). cDNA synthesis was performed using qScript™ cDNA SuperMix (Quanta BioSciences, Inc. cat # 95048). RT-qPCR was performed using TaqMan gene expression assays. A standard 20 ng of RNA input per reaction was used for all qPCR studies. Applied Biosystems 7500 Fast Real-Time PCR System was used to run all reactions. ΔCT method of analysis was performed to assess the relative fold change in the expression of the indicated genes. All gene expression data were normalized to housekeeping genes 18 s, *GAPDH* or *PPIA* according to host species. The list of genes used to determine expression is shown in Supplementary Table 9.

#### Mitochondria movement measurements

Measurement of Mitochondria movement was adapted from Li et al.^80^. shScr and sh*DIAPH1* HiPSC-CMs were initially stained with 200 nM mitochondrial live cell imaging dye MitoTracker™ Red CMXRos (cat# M7512, Thermo Fisher Scientific) for 30 min followed by TimeLapse imaging at 40× magnification using Nikon Eclipse Ti Epifluorescence Microscope (Inverted) at an interval of 0.5 s for 5 mins To automate measurements, plugin using macros was created to generate Kymograms and measure the movement (µm/s) of randomly free moving mitochondria in the frames using Fiji ImageJ software. To generate kymograms and track mitochondria movement, a composite image from multiple frames was initially generated and then the traces of moving mitochondria were tracked using a segmented line tool to get the accurate path to generate a kymogram as published in the literature^81^. The resultant kymogram generated was used to measure speed using the same macros function. Specifically, the vertical lines in the kymograph represent stationary mitochondria, while non-linear, slanted/tilted lines/curves represent motile mitochondria. For a mitochondrion to be considered stationary, displacement of ≤5 μm for the entire recording period was considered immobile. The macros plugin used for quantification has been uploaded accordingly.

#### CS activity

CS activity was measured in HiPSC-CMs using Citrate Synthase Assay Kit (Sigma, cat# CS0720). The assay and calculations were performed according to the manufacturer’s instructions. In brief, HiPSC-CMs were cultured in 60 mm cell culture dishes. After exposure with or without hypoxic conditions, cells were washed with 1× PBS, later extracted for protein with CelLytic MT Cell Lysis Reagent (Sigma cat#C3228) with Protease Inhibitor Cocktail (Sigma cat# P8340). Cells were centrifuged at 16,000×*g* for 10 min to eliminate cell debris. The harvested supernatant was used to perform the assay. According to the protocol, absorbance at 412 nm was recorded using a TECAN plate reader.

#### MitoSOX

Mitochondrial superoxide production was assessed with live cell imaging fluorescence dye MitoSOX (ThermoFisher Scientific, cat# M36008). HiPSC-CMs were cultured on 4 well chamber slides (Labtek cat # 154526). After exposure to or without H/R conditions, the cells were incubated with a 5 µM concentration of dye in a maintenance medium for 10 min. Wells were later washed three times with 1× PBS, followed by fixation in 3.7% paraformaldehyde (PFA). Images were taken at 10× magnification with an RFP filter using EVOS XL Core Imaging System. Microscope capture settings for intensity and exposure were kept constant for all images. A minimum of four images were taken per well. Relative intensity for every image was calculated using NIH ImageJ software.

#### Mitochondrial permeability transition pore (MPTP)

MPTP in HiPSC-CMs exposed to H/R was assessed using a Mitochondrial Permeability Transition Pore Assay Kit (cat# ab239704, ABCAM). Using a flow-cytometry-based approach, the experiment was performed as per the manufacturer’s guidelines.

#### Mitochondrial membrane potential

Mitochondrial membrane potential measurements were measured using Mitochondrial Permeability Transition:MitoPT™ JC-1 Kit (Bio-Rad ICT-944). HiPSC-CMs exposed to H/R were cultured on tissue culture chamber slides. Staining was performed as per the manufacturer’s guidelines. Cells were fixed with 3% PFA. Imaging was performed using a Leica SP8 confocal microscope at 63× magnification. The fluorescence signal for JC-1 green was detected at ex./em. range 488–527 nm. Laser intensity for the 488 nm filter was maintained constant at 5%, and Argon laser power at 20%. The fluorescence signal for JC-1 red was detected at ex./em. range 561 nm to 620 nm. Laser intensity for the 561 nm line was maintained at 5%. Image acquisition settings were kept constant for the complete experiment. DAPI line 405 nm was used at 40% for clear nuclei identification and counting purposes. Fluorescence intensity for green and red channels was quantified using NIH ImageJ software. Data were presented as the ratio of red intensity to green intensity.

#### F/G actin measurements

Filamentous and globular actin in HiPSC-CMs was measured using Alexa Fluor™ 488 Phalloidin (Fisher Scientific cat # A12379) to stain F-actin (green) and Deoxyribonuclease I, Alexa Fluor™ 594 Conjugate (Fisher Scientific cat # D12372) to stain G-actin (red). Imaging was performed using a Leica SP8 confocal microscope at 63× magnification. Fluorescence signal for F-actin was detected at ex. 495 nm and emission at 518 nm. Laser intensity for the 488 nm filter was maintained constant at 10% and Argon laser power at 20%. Fluorescence signal for G-actin was detected at ex. 590 nm and emission at 617 nm. Laser intensity for the 594 nm line was maintained at 10.3%. Image acquisition settings were kept constant for the complete experiment. DAPI line 405 nm was used at 40% for clear nuclei identification and counting purposes. Fluorescence intensity for green and red channels was quantified using NIH ImageJ software. Data are represented as relative intensity of F/G actin to determine changes in actin polymerization under both baseline and H/R in *DIAPH1*-silenced HiPSC-CMs and controls.

#### MitoTimer studies

MitoTimer (MitoT) technique is used to understand the mitochondrial turnover and to distinguish between the young and old or dysfunctional mitochondria^82^. The novel MitoT construct was a generous gift from Dr. Roberta A. Gottlieb from Cedars-Sinai. Due to the difficulties faced to efficiently transfect HiPSC-CMs and to prevent dual transfection with rtTA plasmid (tetracycline-controlled transcription activation), which is expected to decrease transfection efficiency and selection, we constructed a new plasmid that includes both MitoT and rtTA genes in a single construct. pCW entry vector containing hPGK (human phosphoglycerate kinase) promoter for ubiquitous expression of MitoT and rtTA proteins with puromycin resistance gene for positive selection was used (pCW-MitoT-hPGK-puro-rtTA). Transfection was performed using lipofectamine LTX reagent in a 6-well cell culture plate according to the manufacturer’s protocol. The protocol was optimized for 5 µg input of plasmid DNA per each well of a 6-well plate. Forty-eight-hour post transfection, cells were transferred onto 4-well chamber slides. Upon successful recovery on chamber slides, the cells were treated with 2 µg/ml doxycycline for Tet-on induction for 48 h. Cells were fixed with 3.7% PFA, followed by mounting and imaging. For in vivo experiments, alpha-MHC-MitoTimer mice were crossed with global *Diaph1* knockout mice to obtain MitoTimer expression in the hearts^39^. Perfused Baseline and I/R mice hearts were embedded in an optimum cutting temperature (OCT) solution. Cryo-preserved hearts were sectioned at 7 µm thickness. Cryo-sections obtained were further fixed with 3.7% PFA, followed by mounting and confocal imaging. Fluorescence imaging was performed using Leica SP8 confocal microscope at 63× magnification. For mice hearts, the fluorescence signal for green was detected at ex/em range 488–530 nm. Laser intensity for the 488 nm filter was maintained constant at 1.8%, and Argon laser power at 20%. The fluorescence signal for red was detected at ex/em. range 561–590 nm. Laser intensity for 562 nm line was maintained at 2%. For HiPSC-CMs, the fluorescence signal for green was detected at ex/em range 488–530 nm. Laser intensity for the 488 nm filter was maintained constant at 28.42%, and Argon laser power at 20%. The fluorescence signal for red was detected at ex/em. range 561–590 nm. Laser intensity for the 562 nm line was maintained at 29.99%. DAPI line 405 nm was used at 40% for clear nuclei identification and counting purposes. Image acquisition settings were kept constant for the complete experiment. Several randomly selected images were taken per each well. Quantification of fluorescence intensity per image was performed using NIH ImageJ software.

#### Seahorse studies

Mitochondrial stress in HiPSC-CMs was performed using the Seahorse XFp Cell Mitochondria Stress Test Kit by Agilent Technologies (cat # 103015). The study was performed as per the manufacturer’s guidelines. In brief, 50,000 cells were seeded into each well excluding two blank wells of 8-well cell culture miniplates suitable for the XFp analyzer. Concentrations of Oligomycin, FCCP, and Rotenone were used at 2, 5, and 1 µM, respectively. Data for OCR, basal respiration, and ATP production in samples were calculated from Wave software (Agilent Technologies) and normalized to the corresponding total protein concentration.

#### Electron microscopy

Cultured cells and heart tissues were fixed with 2.5% glutaraldehyde and 2% paraformaldehyde in 0.1 M cacodylate buffer and post-fixed with 1% OsO4 in 0.1 M cacodylate buffer. The cells were stained *en bloc* with 1% uranyl acetate, then dehydrated in a graded series of ethanol followed by propylene oxide and embedded with EMbed 812 (Electron Microscopy Sciences, Hatfield, PA). Sections were cut at 70 nm on a Leica EM UC6 ultramicrotome and picked up on copper grids, stained with 3% uranyl acetate for 15 min and Reynolds lead citrate for 5 min. Grids were viewed using a Philips CM12 TEM (Philips) transmission electron microscope and photographed using a Gatan 4k × 2.7k digital camera (Gatan Inc.).

- Electron microscopy: Mitochondria–SR distance measurement. Mitochondria–SR distance was measured, as published in the literature^13,50^ using NIH-ImageJ software. All images were assessed under identical magnification conditions. The straight line segment tool was used to mark the distance between the SR and outer mitochondria membrane and to generate the distance measurements. Before quantification, the global scale was set according to the nm scale. For HiPSC-CMs, we scored 150 Mito-SR contacts obtained from two consecutive batches of cells. For mice, we used the same approach from two mice cohorts/batches. Quantification was obtained from a minimum of 10 different sections imaged from each batch. The processing and sectioning of cells and tissues was performed by NYU microscopy core faculty, who were naïve to the experimental groups, and quantification was performed by investigators naïve to the experimental conditions.

#### Immuno-precipitation studies and Western blotting

For extraction of proteins in HiPSC-CMs, cells were initially washed with 1× PBS and later lysed using CelLytic MT Cell Lysis Reagent (Sigma cat#C3228) with Protease Inhibitor Cocktail (Sigma cat# P8340). Cell lysates were snap-frozen and preserved at −80 °C until further use. For immuno-precipitation studies, Agarose A/G (Cell Signaling cat # 9863/3747) beads were used to pull down the desired proteins. In total, 200 µg of protein was used for immunoprecipitation pull-down studies according to the manufacturer’s guidelines. In brief, protein lysate was incubated with 2 µg of either DIAPH1 (ABCAM cat # ab129167) and MFN2 (ABCAM cat # ab205236) antibodies incubated overnight, followed by the addition of 30 µl of 50% agarose beads incubated for 4 h. Excess unbound protein–antibody was eliminated by washing 5 times with 500 µl cell lysis buffer. The resultant complex was suspended in 30 µl 2× Laemmli buffer. The samples were incubated at 95 °C for 5 min, followed by SDS-PAGE and Western blotting with DIAPH1, MFN2, or MFN1 (ABCAM cat # ab191853) primary antibodies. For secondary antibody and detection, LI-COR IRDye rabbit 800CW and mouse 680RD were used at a dilution of 1:5000. For protein expression studies, 20 µg of protein from the indicated samples was suspended in 1× Laemmli buffer followed by incubation at 95 °C for 5 min followed by SDS page. Details of antibodies used are listed in Supplementary Table 10.

#### Protein–protein interaction studies

Duolink™ PLA technology was used to study the endogenous protein interactions between DIAPH1 and MFN2. Duolink™ In Situ Red Starter Kit Mouse/Rabbit (MilliporeSigma, cat# DUO92101) was used. In brief, cells were cultured on 4- or 8-well chamber slides coated with or without Matrigel according to cell type. For mice, cryocut sections from OCT-preserved heart tissues were used and sections were cut at 7 µm thickness. Cells or OCT-recovered tissue sections were initially fixed with 3.7% PFA; thereafter, the detailed protocol was followed as described by the manufacturer. For human biopsies, the tissue was processed for paraffin sectioning of 7 µm thickness, followed by the staining protocol. Monoclonal rabbit anti-DIAPH1 (Abcam, cat# ab129167) targeting the c-terminus of DIAPH1 amino acid sequence and mouse anti-MFN2 (Abcam, cat# ab56889), which have been validated for non-specific binding, were employed. All other antibodies used for DUOLINK PLA studies (Supplementary Table 10) were previously validated for specificity by manufacturers or from the literature. The fluorescence signal of interactions was detected at an excitation of 594 nm and emission at 624 nm. Images were captured using a Leica SP8 confocal microscope at 63× magnification. The total number of interactions per cell and normalized with DAPI counter nuclear stain was quantified using NIH ImageJ and plotted using Prism GraphPad software.

#### DIAPH1–MFN2 PLA and SR localization studies

HiPSC-CMs were initially exposed to H/R as we have observed highest number of DIAPH1–MFN2 interactions under these conditions. To study DIAPH1–MFN2 interactions and ER localization, live cells were initially stained with 500 nM ER-Tracker™ Green (BODIPY™ FL Glibenclamide), (Fisher Scientific cat # E3425) for 45 min. Post-staining, cells were fixed with 3.7% PFA and DUOLINK PLA assay was performed as described above. Images were captured using a Leica SP8 confocal microscope at 63× magnification objective followed by manual zoom to obtain the best images for the region of interest. Fluorescence signal for ER tracker was detected at ex. 488 nm and emission at 514 nm.

#### Annexin V-FITC apoptosis staining

Apoptosis in HiPSC-CMs was measured using commercially available Annexin V-FITC Apoptosis Staining/Detection Kit (ABCAM; cat # ab14085) as per manufacturer’s instructions. In brief, HiPSC-CMs were cultured in 4 well chamber slides. Post-exposure to H/R, cells were incubated with Annexin V-FITC stain for 5 min, followed by fixation and mounting using a DAPI mounting medium. Images were captured using a Leica SP8 confocal microscope at 63× magnification objective. The fluorescence signal for Annexin V-FITC was detected at ex. 488 nm and emission at 530 nm. Laser intensity for the 488 nm filter was maintained constant at 15%, and Argon laser power at 30%. DAPI line 405 nm was used at 40% for clear nuclei identification and counting purposes. Image acquisition settings were kept constant for the complete experiment. Fluorescence intensity quantification for Annexin V-FITC and nuclei count was performed using NIH ImageJ software.

#### Immuno-electron microscopy: Tokuyasu method

Cells exposed to H/R were fixed with 2% paraformaldehyde in 0.1 M phosphate buffer (PB) containing 0.05% glutaraldehyde, pH 7.2–7.4 for 2 h on ice, then changed to 2% paraformaldehyde in 0.1 M phosphate buffer and continue fixing overnight at 4 °C. The cells were washed with 0.1 M PB 3 times and 5 mins each, then treated with 50 mM glycine in PB for 30 mins to quench unbounded aldehyde. After 10% gelatin embedding, the cells were infused with 2.3 M sucrose, mounted on cryosection sample chucks, and stored in liquid nitrogen. Cryosections were cut at 90 nm, and incubated with DIAPH1 rabbit monoclonal antibody (AB129167, 1:10 or 1:20 dilution), followed by application of protein A or goat anti-rabbit conjugated secondary antibodies (18 nm Colloidal Gold-AffiniPure Goat Anti-Rabbit IgG (H + L), Jackson ImmunoReasearch Laboratories, Inc., West Grove, PA); 15 nm Protein A Gold, Cell Microscopy Center, University Medical Center Utrecht, 35584 CX Utrecht, The Netherlands); or MFN2 mouse monoclonal antibody (1:5 or 1:10 dilution) followed by application of goat anti-mouse conjugated secondary antibodies (18 nm Colloidal Gold-AffiniPure Goat anti-mouse IgG (H + L), Jackson ImmunoReasearch Laboratories, Inc., West Grove, PA). The sections are washed with PBS, fixed with 1% glutaraldehyde in PBS, washed with water, then stained and embedded with a mix of % methyl cellulose-uranyl acetate. All stained grids were examined under the JEOL1400 Flash electron microscope (Japan) and photographed with a Gatan Rio 16 camera (Gatan Inc., Pleasanton, CA).

#### SR stress staining

Staining of SR in HiPSC-CMs exposed to H/R was performed using ER-ID® Red assay kit (GFP-CERTIFIED®) (Enzo Life Sciences, cat # ENZ-51026-K500). The experiment was performed as per the manufacturer’s instructions. In brief, live cells in 4 well chamber slides were stained using 1× staining solution containing 1 µl of ER-ID Red Detection Reagent in 1 ml of 1× assay buffer provided in the assay kit for 30 min at 37 °C protected from light. Cells were washed twice with PBS, followed by fixation with 3.7% PFA, and mounted with DAPI mounting solution. Images were captured using a Leica SP8 confocal microscope at 63× magnification objective. The fluorescence signal for ER-ID® Red was detected at ex. 561 nm and emission at 590 nm. Laser intensity for the 561 nm line was maintained at 7%. DAPI line 405 nm was used at 40% for clear nuclei identification and counting purposes. Image acquisition settings were kept constant for the complete experiment. Fluorescence intensity quantification for ER-ID® Red and nuclei count was performed using NIH ImageJ software.

#### Measurement of phosphatidylserine and phosphatidylcholine

Fluorometric measurements of phosphatidylserine and phosphatidylcholine were performed using commercially available kits from ABCAM as per the manufacturer’s protocol. (Phosphatidylserine Assay Kit (Fluorometric) cat # ab273295; Phosphatidylcholine Assay Kit (Colorimetric/Fluorometric) cat# ab83377). HiPSC-CMs were initially incubated with 0.3 g/L l-Serine (cat # S4311, MilliporeSigma) for 72 h with one medium change at 48 h followed by exposure to H/R. HiPSC-CMs, after exposure to H/R, were detached and collected in 1.7 ml collection tubes using a gentle dissociation medium. After washing twice with PBS, cells were re-suspended in 250 µl of ice-cold PBS followed by three times snap freeze/thaw cycles and centrifuged at 16,000×*g* for 10 min. A supernatant devoid of cell debris was used to perform measurements. Phosphatidylserine was measured at Ex/Em = 538/587 nm, and phosphatidylcholine was measured at Ex/Em = 535/587 nm using a TECAN Infinity Pro 200 plate reader. Data were analyzed using instructions outlined in the protocol.

#### Calcium studies

All studies were performed in H9C2 cells with either sh*Diaph1* or shScr conditions. To measure calcium in the ER lumen, we used recently identified^43^ Mag-Fluo-4 AM dye, and for cytosolic calcium measurements, we used Fluo-4 AM dye. (Mag-Fluo-4, AM, cell permeant, cat # M14206, Fluo-4, AM, cell permeant cat # F14217, Thermo Fisher Scientific Inc.). In brief, the H9C2 cells were exposed to 30 min hypoxia followed by dye incubation during 1 h reoxygenation. 20 µM Mag-Fluo-4 AM was diluted in HEPES-buffered saline (HBS: 135 mM NaCl, 5.9 mM KCl, 11.6 mM HEPES, 1.5 mM CaCl_2_, 11.5 mM glucose, 1.2 mM MgCl_2_, fatty acid-free BSA (1 mg/mL) and pluronic acid (0.02 % w/v, pH 7.3) and cells were incubated for 60 mins at room temperature. For Fluo-4 AM, cells were incubated in the same buffer with 5 µM dye for 45 min at 37 °C. After loading dye, cells were washed twice with HBS, followed by fluorescence kinetic measurements with excitation at 490 nm and emission at 517 nm with a time interval of 15 s using a TECAN infinity pro 200 plate reader. After basal fluorescence intensity recording was completed, cells were exposed to 20 mM caffeine final concentration per well, followed by recording measurements for at least 5 mins. ER, calcium release was calculated as the difference (Δ) between basal and the value after caffeine stimulation.

#### Expression and purification of DID

The expression plasmid coding for DID (*pet28-DID*) with an N-terminal tag (sequence MGSSERSHHHHHHSGSE) was purchased from Genscript. The DID construct used here comprises residues 142–380 of human protein diaphanous homolog 1 (Uniprot entry O60610) was purchased from Genscript. *E. coli* BL21(DE3) cells transformed with *pet28-DID* were grown overnight at 37 °C on an agar plate containing Luria–Broth (LB) and 35 µg/mL kanamycin. A colony from this plate was inoculated into a 13-mL LB starter culture with 35 µg/mL kanamycin, grown overnight at 37 °C. The starter culture was transferred to 1 L of LB with 35 µg/mL kanamycin and incubated at 37 °C until the optical density at 600 nm (OD_600_) reached 0.70. To induce overexpression of DID without stable isotope labeling, isopropyl β-d-1-thiogalactopyranoside (IPTG) was added to the LB culture to a final concentration of 1 mM and cells were incubated for 4 h at 37 °C.

To overexpress DID with *U*-^15^N labeling, cells from the LB culture at an OD_600_ of 0.70 were harvested by centrifugation at 4000×g and resuspended in minimal medium at pH 7.0 (5 mM Na_2_HPO_4_, 2.5 mM KH_2_PO_4_, 1 mM NaCl, 2 mM MgSO_4_, 0.1 mM CaCl_2_, and 1 mg/L thiamine hydrochloride) supplemented with 35 µg/mL kanamycin. For *U*-^15^N labeling, [^15^N]-ammonium chloride (99%, 1 g) was used as the sole nitrogen source (Sigma Aldrich), and 2 g of glucose was used as the sole carbon source. IPTG at a concentration of 1 mM induced overexpression of *U*-^15^N labeled DID for 4 h at 37 °C.

Cells were harvested by centrifugation at 4000×*g*. The harvested cell pellet was resuspended in a lysis buffer (10 mM Tris, 10 mM Na_2_HPO_4_/NaH_2_PO_4_, 4 M urea, 1 M NaCl, and 12.5% (w/v) sucrose at pH 8.0) supplemented with a protease inhibitor cocktail (Roche) and sonicated. The lysate was centrifuged for 1 h at 48,400×*g* at 4 °C. The supernatant was loaded onto a nickel-agarose affinity column (Qiagen) equilibrated with lysis buffer. The partially denatured protein bound to the column was refolded by washing the column with refolding buffer (10 mM Na_2_HPO_4_/NaH_2_PO_4_, 300 mM NaCl, 10 mM imidazole at pH 8.0). The column was washed with wash buffer (10 mM Na_2_HPO_4_/NaH_2_PO_4_, 300 mM NaCl, 20 mM imidazole at pH 8.0) followed by elution buffer (10 mM Na_2_HPO_4_/NaH_2_PO_4_, 300 mM NaCl, 250 mM imidazole at pH 8.0). The eluted protein was dialyzed overnight against 20 mM Na_2_HPO_4_/NaH_2_PO_4_, 300 mM NaCl pH 8.0 at 4 °C. The purified DID was concentrated and stored at −80 °C with 20% (v/v) glycerol until use in NMR or cross-linking mass spectrometry experiments.

The concentration of DID was determined via absorbance of the tryptophan residue at 280 nm using an extinction coefficient of 10,220 M^−1^ cm^−1^. Unlabeled DID has a molecular weight of 29,078 Da (calculated using the EXPASY ProtParam tool)^38^.

#### Expression and purification of MFN2

The MFN2 construct used in NMR and cross-linking mass spectrometry studies comprises the sequence of modified, truncated human MFN2 (deleted residues 1–19 and 401–705 from full-length human MFN2 corresponding to Uniprot entry O95140) appended to an N-terminal His-tag (Supplementary Table 1). Similar to the construct previously used in the study of Li et. al.^35^, the construct includes the GTPase and HD1/2 domains of full-length MFN2. The gene was inserted into a kanamycin-resistant expression plasmid *pet28-MFN2* purchased from Genscript. The plasmid was transformed into BL21(DE3) cells and grown on an LB-agar plate with 35 µg/mL kanamycin overnight at 37 °C. A 13 mL starter culture of Terrific Broth (TB) containing 35 µg/mL kanamycin was grown overnight at 37 °C from a single colony of transformed cells. This starter culture was introduced into IL TB containing 35 µg/mL kanamycin and grown to an OD of 0.60. IPTG (100 µM) was added to the TB culture to induce overexpression, which was carried out overnight at 18 °C.

The cells were harvested by centrifugation at 4000×*g*, then resuspended in a lysis buffer composed of 50 mM HEPES pH 7.5, 1 M NaCl, 30 mM imidazole, 2.5 mM β-mercaptoethanol, 1 µM DNase I, and a protease inhibitor cocktail (Roche). The cells were lysed by sonication followed by incubation for 15 mins at 37 °C to allow digestion of DNA. The lysate was centrifuged at 40,000×*g* for 50 min, and the supernatant fraction was loaded onto a nickel-agarose affinity column (Qiagen) equilibrated with 20 mM HEPES, pH 7.5, 1 M NaCl, 30 mM imidazole. The column was washed with the same buffer and MFN2 was eluted by increasing the concentration of imidazole to 300 mM. The eluted protein was dialyzed overnight at 4 °C against 20 mM HEPES, pH 7.5, 150 mM KCl, 5 mM MgCl_2_, and 1 mM DTT.

Since MFN2 is a weak GTPase^35^ and the presence of bound nucleotide does not change the MFN2 structure (PDB entries 6JFL(apo) and 6JFK(GDP-bound) have an RMSD of 1.5 Å), the apo form was used for all experiments. The dialyzed protein was concentrated to 2 mL and reacted with Quick Calf Intestinal Phosphatase (CIP, purchased from New England Biolabs) at 25 °C to hydrolyze GDP bound to MFN2. Any precipitate that formed as a result of the hydrolysis reaction was removed by centrifugation and the supernatant was diluted by a factor of 10 with lysis buffer. The GDP-free sample was subjected to a second round of the nickel-agarose affinity chromatography procedure described above, and the excess imidazole from eluted GDP-free protein was removed by dialysis. The dialyzed sample was concentrated and then frozen at −80 °C with 20% (v/v) glycerol until further use. The concentration of MFN2 was determined via absorbance of the tryptophan residue at 280 nm using an extinction coefficient of 35,200 M^−1^ cm^−1^ ^38^.

### Expression and purification of DAD^M1199L^ mutant

The DNA sequence for the M1199L mutant peptide, DAD^M1199L^, was purchased from Genscript and inserted into pTM-7 to express an N-terminal his-tagged TrpL-DAD^M1199L^ fusion protein^83^. Fusing the DAD^M1199L^ peptide to a hydrophobic protein (TrpL) directed the gene product into inclusion bodies. The methionine residues in TrpL were mutated to leucine residues^84^ to allow for a single cyanogen bromide cleavage site between TrpL and DAD^M1199L^. The M1199L mutation is conservative and does not change the biding affinity to DID. The plasmid was transformed into *E. coli* BL21(DE3) to overexpress both labeled and unlabeled peptides as described for the DID constructs.

To purify DAD^M1199L^, the bacterial cell pellet was resuspended in 50 mM Tris-HCl, pH 7.2, 1% w/v Triton-X-100 and 1 mM EDTA, sonicated as described above, and centrifuged at 10,000*g* for 30 min at 4 °C. The pellet was washed with a series of buffers, 50 mM Tris-HCl, pH 7.2, containing 2% w/v Triton-X-100 and 1 mM EDTA, followed by 25 mM Tris-HCl, pH 7.2 containing 1 M NaCl and 0.5 mM EDTA, and 50 mM Tris-HCl, pH 7.2. The cells were sonicated and centrifuged between washes. The final pellet was dissolved in denaturing buffer, 50 mM sodium phosphate, pH 8.3, containing 6 M guanidinium chloride, and clarified by centrifugation at 10,000*g*. The supernatant was incubated with Ni-NTA beads overnight for batch binding. The beads were packed into a column and washed once with denaturing buffer. The column was washed with 5 volumes of 50 mM sodium phosphate, pH 7.0, containing 6 M urea, followed by 50 mM sodium phosphate, pH 6.4 and 6 M urea. Elution was carried out using 50 mM sodium phosphate, pH 3.6 and 6 M urea. Fractions containing TrpL-DAD^M1199L^were pooled, dialyzed into water, and lyophilized. Cleavage was performed by dissolving the TrpL-DAD^M1199L^ fusion protein in 70 % formic acid with a 100 molar excess of cyanogen bromide and incubating for 1.5 h at 25 °C. The cleaved products were dried *in vacuo*, and loaded onto a C18 column (Agilent ZORBAX 300SB-C18) for high-performance liquid chromatography (HPLC). Cleaved products were resolved using a gradient of 0 %-90 % acetonitrile in 0.1% trifluoroacetic acid. DAD^M1199L^ was eluted first and was collected, lyophilized, and stored at −20 °C. DAD^M1199L^ stock solutions were prepared by dissolving the peptide in 10 mM potassium phosphate buffer, pH 6.8, containing 15 mM hexamethylphosphoramide (HMPA). The concentration of DAD^M1199L^ stock solutions was determined by integrating HPLC chromatogram traces at 260 nm, the approximate wavelength of maximal absorption of phenylalanine residues relative to reference wild-type DAD peptide solutions.

#### NMR titration experiments

Protein samples of [*U*-^15^N]-DID were prepared in NMR buffer: 20 mM potassium phosphate, 1 mM 4-(2-aminoethyl)benzenesulfonyl fluoride hydrochloride (AEBSF), 0.5 mM ethylenediaminetetraacetic acid (EDTA), 1 mM dithiothreitol (DTT), 100 mM sodium chloride, 15 mM HMPA, and 10% v/v D_2_O at pH 7.5. DTT was added to ensure that cysteine residues in the sample would be in a reduced state.

^1^H-^15^N HSQC^36^ spectra of 100 µM [*U*-^15^N]-DID were monitored at 305 K upon the addition of MFN2 (25 µM up to 100 µM). To examine the effect of DAD on DID-MFN2 binding, synthetic DAD peptide (DETGVMDSLLEALQSGAAFRRKRGPRQAN, purchased from GenScript) was added to the mixture containing 100 µM [*U*-^15^N]-DID and 100 µM MFN2. DAD concentrations in the mixture ranged from 50 µM up to 500 µM.

To probe possible MFN2-DAD interactions, ^1^H-^15^N HSQC^36^ spectra of 100 µM [*U*-^15^N]-DAD^M1199L^ were monitored at 305 K upon the addition of 125 µM MFN2.

NMR spectra were recorded using a Bruker Avance II spectrometer with a proton Larmor frequency of 700 MHz and equipped with a TXI cryoprobe. All spectra were processed using Topspin 2.1 (Bruker).

### Cross-linking reaction

Cross-linking was performed using homo-bifunctional (amine-to-amine) and hetero-bifunctional (amine-to-sulfhydryl) cross-linkers (Supplementary Table 2). Cross-linkers (Thermo Fisher) were added to DID/MFN2 mixtures in cross-linking buffer (20 mM potassium phosphate buffer at pH 7.5 with 100 mM NaCl) to a final concentration of 250–500 µM cross-linker. Each reaction mixture contained 50 µM DID and 50 µM MFN2. The following amine-to-amine cross-linkers were used: disuccinimidyl glutarate (DSG), bis(sulfosuccinimidyl) suberate (BS3), bis(succinimidyl) penta(ethylene glycol) (BS(PEG)_5_), and bis(succinimidyl) nona(ethylene glycol) (BS(PEG)_9_). These amine-to-amine cross-linkers had spacer arm lengths of 7.7, 11.4, 21.7, and 35.8 Å, respectively. The amine-to-sulfhydryl cross-linkers used were N-(k-Maleimidoundecanoyloxy) sulfosuccinimide ester (KMUS), sulfosuccinimidyl 4-(*N*-maleimidomethyl)cyclohexane-1-carboxylate (SMCC), and succinimidyl 4-(*p*-maleimidophenyl)butyrate (SMPB) had spacer arm lengths of 16.3, 8.3, and 11.6 Å, respectively. The reaction mixtures were incubated for 1–2 h at 25 °C, after which cross-linking was quenched by the addition of quenching buffer (0.5 M Tris at pH 7.5 and 10 mM betamercaptoethanol) to a final concentration of 62 mM Tris and 1.24 mM betamercaptoethanol.

Cross-linked proteins were resolved by sodium dodecyl sulfate polyacrylamide gel electrophoresis (SDS-PAGE), using an 8% acrylamide resolving gel. Cross-linked DID/MFN2 bands (appearing at ~75 kDa on the acrylamide gel) were excised from Coomassie-stained gels and cut into ~1 mm^3^ pieces. Gel pieces were destained in water and washed with 100 mM NH_4_HCO_3_ with 22 mM tris(2-carboxyethyl)phosphine (TCEP). The mixture was centrifuged to separate the gel pieces from the supernatant, and the wash with TCEP was repeated. The TCEP-treated gel pieces were dehydrated by soaking in acetonitrile (ACN) twice, then dried by speed vacuum centrifugation.

Dried gel pieces were subsequently treated with 22 mM TCEP in 100 mM NH_4_HCO_3_ at 37 °C for 30 min. Gel pieces were dehydrated again using ACN and dried by speed vacuum centrifugation, after which they were treated with 40 mM iodoacetamide in 100 mM NH_4_HCO_3_ for 30 min at 37 °C in the dark. Excess liquid was removed and the gel was once again dehydrated using ACN and dried.

In-gel digestion was then performed by incubating the gel pieces in a solution of 12.5 ng/µL trypsin in 100 mM NH_4_HCO_3_ overnight at 37 °C. The excess liquid was removed, and digested peptides were extracted from the gel pieces by washing with 5% (v/v) formic acid and 50% (v/v) ACN in water. The extracts were concentrated by speed vacuum centrifugation and stored until use in LC–MS/MS analysis.

### Mass spectrometry

Cross-linked peptides were resuspended in 0.1% (v/v) trifluoroacetic acid and 3.0% (v/v) ACN in water. In a typical experiment, 30 µL aliquots of the peptide samples were trapped and desalted isocratically on a home-packed C18 pre-column cartridge Everest C18 (Grace) for 6 min with 0.1% formic acid delivered by the auxiliary pump at 40 μl/min. The peptides were then eluted from the pre-column and separated on a C18 capillary column (15 cm × 500 μm internal diameter) packed with C18-300 particles (5 µm resin, Advanced Chromatography Technologies). Peptides were eluted at a flow rate of 20 µl/min with a 40-min gradient of 5-to-80% ACN in 0.1% formic acid. The C18 column was connected in line with a Thermo Scientific Orbitrap Velos mass spectrometer operating with an emitter voltage of 4.5 kV and ion transfer tube temperature at 275 °C. The Velos was operated in a data-dependent acquisition mode where multiply charged ions with abundance >6000 cps were selected for the MS/MS fragmentation. Full scan mass spectra (350–2000 m/z, 30,000 resolution) were detected in the orbitrap analyzer after the accumulation of 10^6^ ions. Peptides with multiple charges were selected automatically and fragmented in the collision cell through high-energy collision dissociation with 34% normalized collision energy at a resolution of 7500. A lock mass of diisooctyl phthalate, m/z 391.28428, was used for mass calibration. Data analysis was performed using pLink version 2^85^. The cross-linking mass spectrometry was performed at the RNA Epitranscriptomics & Proteomics Resource located at the Life Sciences Building, University at Albany, supported by the SUNY Research Foundation.

### Computational docking

The Multi-body interface of HADDOCK (high ambiguity driven docking) version 2.2^86^ was used to dock one DID-DD dimer and two MFN2 monomers to form the DID–DD–MFN2 A_2_B_2_ tetrameric complex (Supplementary Tables 2-7**)**. Structural coordinates of the human DID-DD dimer (residues 142-444 on each monomer following residue numbering in Uniprot O60610) were from the homology model constructed using SWISS-MODEL^87^. To make this model, the sequence of human DID–DD was aligned with the sequence of mouse DID–DD, using PDB entry 3EG5^88^ as a template. Structural coordinates of MFN2 (residues 24–400, 706–756 of full-length human MFN2 following residue numbering in Uniprot O95140) were taken from PDB entry 6JFK^35^.

Positive cross-links identified between DID and MFN2 by mass spectrometry were used to generate UIRs between DID–DD and MFN2 monomers (Supplementary Table 2). C2 symmetry constraints were employed within the DID–DD dimer, as well as between two MFN2 monomers during the docking to generate an A_2_B_2_ tetramer with overall C2 symmetry. AIRs, when used in the docking protocol, were generated by identifying the interaction surface (residues in DID within 5 Å of the molecular surface of DAD, selected by Chimera)^89^ between DID and DAD from PDB entry 2F31^37^. These residues were thus defined as active residues in DID–DD, while active residues in MFN2 were those involved in positive cross-links (Supplementary Table 3).

Two types of docking protocols were used, which differed only in the presence or absence of AIRs. The parameters for these protocols are summarized in Supplementary Table 6. The docking solutions that had the least violations (using either protocol) (Supplementary Table 4) were chosen to represent the MFN2–DID–DD tetrameric complex. These models belonged to the ‘most reliable’ cluster of docking solutions, which had the lowest HADDOCK scores. Docking statistics are summarized in Supplementary Table 7. Comparing a representative structure from each docking protocol yielded a Cα RMSD of 8.4 Å. Nine out of 18 unambiguous distance restraints applied were followed, meaning distance violations were at or below 5 Å, which is the upper and lower bound correction defined in the docking protocol. Three of these distance violations had a magnitude of 5.1 Å, while six had magnitudes in the range 7.5–29.1 Å. Notably, the six largest violations are found in the region spanning DID and the GTPase domain of MFN2, and these distance constraints came from the detection of residue pairs cross-linked using SMCC. It is possible that the bulky ring in the spacer arm of SMCC interfered with the binding between DID and the GTPase domain of MFN2, yielding a cross-linked conformation that deviated from that cross-linked using DSG and BS(PEG)_9_ (both of which have linear spacers). In addition, it has been reported that the GTPase domain of MFN2 changes conformation upon binding to GDP^35^, therefore it is also possible that the residues in this domain exhibit some degree of flexibility that allows MFN2 to form more contacts with DID–DD, which would correspond to shorter distances between the DID–DD–MFN2 residue pairs cross-linked by SMCC.

Figures of the docked DID–DD–MFN2 A_2_B_2_ complex were made using Chimera version 1.14^89^ or Discovery Studio^90^. Chimera^90^ MatchMaker version 1.14 was used to superpose representative docked solutions^91^. Swiss PDB viewer version 4.1.0 was used to calculate Cα RMSD^92^. PQR files were created from the structural coordinates of the MFN2 and DID–DD segments, and DelPhi was used to calculate the electrostatic potentials of each segment using an ionic strength of 0.3, solvent dielectric of 80, and solute dielectric of 2^93^.

#### Plasmids

Plasmid *mEmerald-Calreticulin-N-16*, which was used as an ER marker, was purchased from Addgene Inc. Plasmid *pECFP-Mito*, which was used as a mitochondrial marker, was obtained from Takara Bio. Plasmid *pTagRFP-N*, which was used as a vector for *DIAPH1* cloning, was obtained from Evrogen Inc. A fragment containing human *DIAPH1* was PCR amplified using source clone #401258087 (Open Biosystems) as a template and inserted into the vector at *XhoI* and *AgeI* sites upstream of a fluorescent protein sequence to obtain *DIAPH1-RFP*.

#### Sample preparation for confocal microscopy and plate assays

HEK293T cells were plated on 15 mm glass coverslips (Ted Pella, Inc.) and treated with collagen type I (Sigma). Cells were grown at 37 °C, with 5% CO_2_ in low glucose DMEM medium (Thermo Scientific) supplemented with 10% FBS, to 50–70% confluency. Transfections were carried out with FuGene 6 reagent (Promega) according to the manufacturer’s recommendations. Cells were incubated with transfection mix for 36 h, washed with PBS, and fixed in 4% formaldehyde/PBS for 20–30 min at 37 °C. Coverslips were washed 3 times with 1 mL of PBS and mounted with Fluoromount G (Electron Microscopy Sciences).

For plate assays, HEK293T cells were transfected with *DIAPH1-eCFP* construct^63^ and harvested after 3 d expression. Cells were lysed with the RIPA buffer on ice; the lysates were centrifuged; the supernatants were stored at −80 °C. The amount of DIAPH1-eCFP in the extracts was estimated by comparing the fluorescence of commercially available recombinant CFP (BioVision) and lysates. Measurements were run on the Synergy H1 plate reader (BioTek Instruments) in 384 well Fluotrac plates (Greiner) at 456 nm excitation and 492 nm emission wavelength. Concentrations of DIAPH1-eCFP were estimated to be in the range of 60–77 nM, averaging at about 70 nM. Images were acquired on Zeiss LSM 710 confocal imaging system equipped with X63, 1.4 NA oil immersion objective. 405 nm UV laser at 5.5% power was used for the DAPI excitation, 458 nm argon laser line at 14% power—for the eCFP excitation, 488 nm line at 3 % power—for the Emerald excitation, and 561 nm line at 2% power—for the RFP excitation. The emission window of 411–453 nm was used for the DAPI emission registration, 462–480 nm for CFP, 494–523 nm for Emerald, and 566–633 nm for RFP.

#### Binding assays

Clear High Bind 96-well plates (Corning) were coated with MFN2 (0.1 μM MFN2 in 50 μl Na_2_CO_3,_ pH=9.6) overnight at 4 °C. Maximum binding capacity was estimated in a separate experiment to be about 80 ng (1.635 pMol) of MFN2 per well. Wells were washed two times with PBS, two times with PBST (PBS containing 0.05% Tween 20), and blocked with 3% BSA in PBST for 3 h at room temperature. BSA was removed from preselected wells; the wells were washed two times with TBS (Tris-buffer saline) and treated with 2 μM of DID in 50 μl TBS pH 7.9 overnight at 4 °C. All wells were washed three times with PBST, and varying amounts of cell lysates were added in 50 μl of TBS pH = 7.2 or TBS pH 6.2 per well. Plates were incubated overnight at 4 °C, then washed four times with PBST, and anti-GFP antibody-HRP conjugate (ABCAM) was added at 1:5000 dilution in 50 μl PBST with 3% BSA. Plates were incubated overnight at 4 °C, washed three times with PBST, two times with PBS, and then 75 μL of TMB substrate (Thermo Fisher) was added. Plates were incubated for 20 mins at room temperature and reaction was terminated by the addition of 75 μl of 0.18 M sulfuric acid. A Synergy H1 plate reader (BioTek Instruments) was used to measure absorbance at 460 nm, and background signals from the wells with no MFN2 were subtracted. The resulting data were fit with “one site—total and nonspecific binding” regression model in GraphPad Prism version 9.0 software (GraphPad).

#### Linker studies

In order to physically reduce the distance between mitochondria and ER, we performed linker studies^13^. For targeting the outer mitochondrial membrane surface, N-terminal mitochondrial localization sequence V84389 of mAKAP1 (MAIQLRSLFPLALPGLLALLGWWWFFSRKK) was fused to N terminus of FKBP12 through the linker sequence DLELKLRILQSTVPRARDPPVAT. The recombinant protein was tagged with RFP for fluorescence labeling. Similarly, for targeting ER, the C-terminal localization sequence UBC6 protein from Yeast (MVYIGIAIFLFVGLFMK) fused with the same linker sequence was fused to FRB. The fused protein was tagged with YFP for fluorescence detection of the expressed sequence. Both the constructs were inserted into the pcDNA3-eGFP backbone for mammalian expression. Cells were treated with a short burst of rapamycin at a concentration of 100 nM for 10 min for the formation of FKBP12–FRB heterodimerization and linking both mitochondria and ER together. After 10 min, cells were replaced with fresh medium before being exposed to H/R stress. This process of low-dose treatment at 100 nM for 10 min is not sufficient to trigger any physiological response^13,94^. To validate the successful transfection of constructs, confocal microscopic images at 63× magnification were taken to show cells with positive RFP and YFP signals. All experiments were performed under H/R stress as described above. Both *shScr* and *shDIAPH1* HiPSC-CMs were transfected with mitochondria and SR constructs with or without fluorescent tags. To obtain the desired distance of 5 nm in *DIAPH1*-silenced groups, cells were treated with rapamycin, whereas the other two groups shScr and sh*DIAPH1*, received vehicle (VEH, DMSO) as controls.

### Statistics and reproducibility

All data, unless otherwise mentioned in the text, have been obtained for at least a minimum of two independent experiments performed at different time points. Shapiro–Wilk test for normality was performed for each group of data before performing any pairwise comparisons.

### Reporting summary

Further information on research design is available in the Nature Portfolio Reporting Summary linked to this article.

### Supplementary information

Supplementary Information

Description of Additional Supplementary Files

Supplementary Movie 1

Supplementary Movie 2

Reporting Summary

### Source data

Source data

## Data Availability

The authors declare that the data supporting the findings of this study are available within the paper and its Supplementary Information. Human iPSC derived CM raw and aligned sequencing data, resulting raw and normalized count data, and supporting sample data are publicly available through NCBI GEO database with accession number: GSE214149. All biological materials used in this study are available as indicated in the Methods section (all sources and catalog numbers are provided). Research material are available upon request. All mass spectrometry data were deposited to ProteomeXchange and jPOST sites with accession numbers PXD045744 and JPST002335, respectively. Source data are provided with this paper.
