# Supplementary material for: DIAPH1-MFN2 interaction regulates mitochondria-SR/ER contact and modulates ischemic/hypoxic stress
Source: Nat Commun. 2023 Oct 30;14:6900. doi: 10.1038/s41467-023-42521-x (PMC10616211; doi:10.1038/s41467-023-42521-x)
Supplement: Supplementary file 1 — Supplementary Information [file 41467_2023_42521_MOESM1_ESM.pdf]

Supplementary Information:

DIAPH1-MFN2 interaction regulates mitochondria-SR/ER contact and modulates ischemic/hypoxic stress

Gautham Yepuri[1], Lisa M. Ramirez[2], Gregory G. Theophall[2], Sergei V Reverdatto[2], Nosirudeen Quadri[1], Syed Nurul Hasan, Lei Bu[3], Devi Thiagarajan, Robin Wilson[1], Raquel López Díez[1], Paul F. Gugger[1], Kaamashri Mangar[1], Navneet Narula[4], Stuart D. Katz[3], Boyan Zhou[5], Huilin Li[5], Aleksandr B. Stotland[6], Roberta A. Gottlieb[7], Ann Marie Schmidt[1], Alexander Shekhtman[2#], Ravichandran Ramasamy[1#*]

[1]Diabetes Research Program, Division of Endocrinology, Diabetes and Metabolism, Department of Medicine, NYU Grossman School of Medicine, New York, New York 10016
[2]Department of Chemistry, University of Albany, State University of New York, Albany, New York, 12222
[3]Department of Medicine, Leon H. Charney Division of Cardiology, NYU Grossman School of Medicine, New York, New York 10016
[4]Department of Pathology, NYU Grossman School of Medicine, New York, New York 10016
[5]Department of Population Health, NYU Grossman School of Medicine, New York, New York 10016
[6]Department of Cardiology, Smidt Heart Institute, Cedars-Sinai Medical Center, Los Angeles, CA 90048
[7]Department of Biomedical Sciences, Smidt Heart Institute, Cedars-Sinai Medical Center, Los Angeles, CA 90048

*Corresponding Author, email: ravichandran.ramasamy@nyulangone.org,

# These authors jointly supervised this work

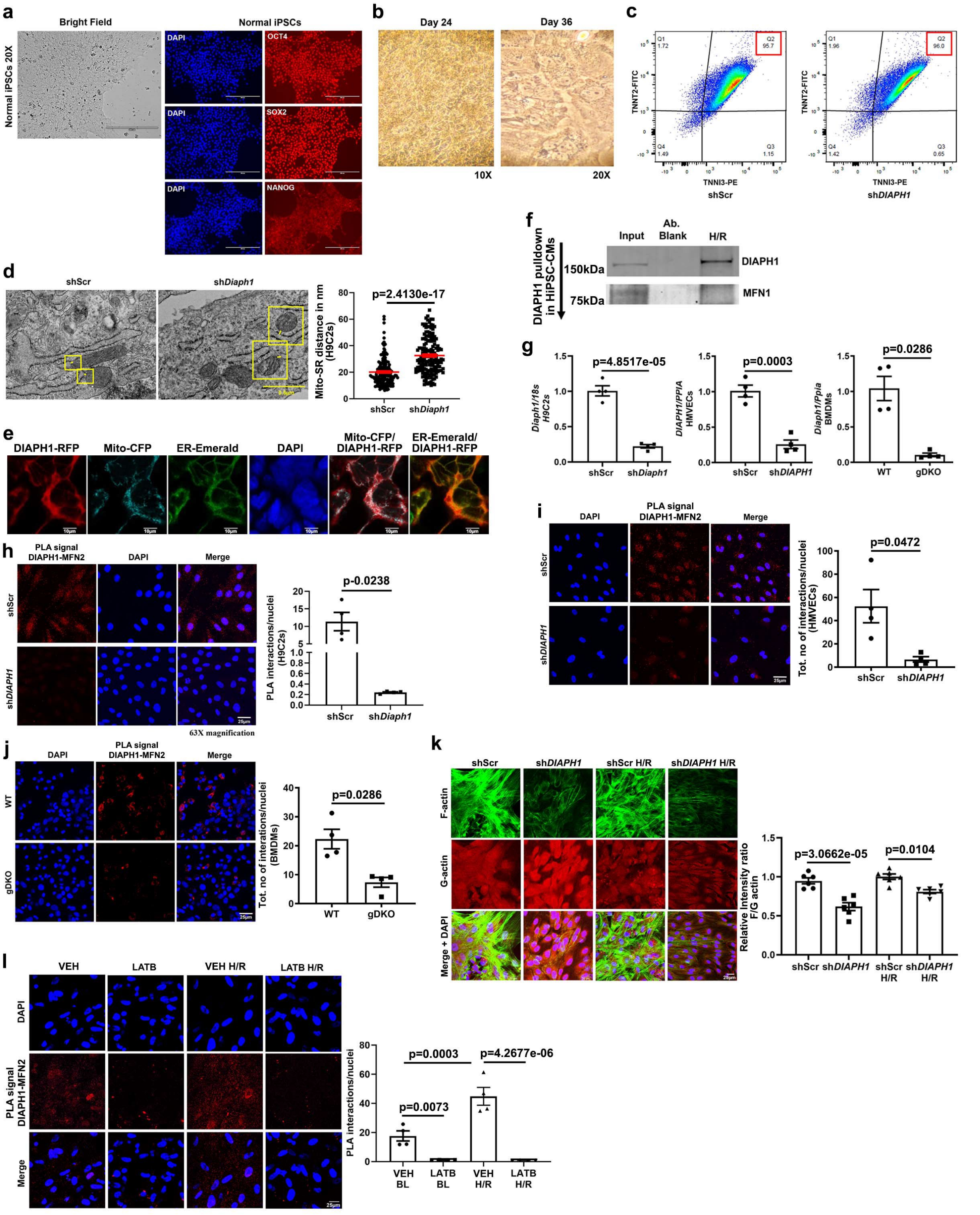

Supplementary figure 1| Data supporting figure 1: a. represents bright field image with clean borders of iPSC colony successfully reprogrammed from human adult dermal fibroblasts and IF

staining for positive iPSC markers OCT4/SOX2 and NANOG. Scale bar 200 μm. **b.** Videos represent beating of CMs differentiated from HiPSCs at day 24 using 10x and day 36 using 20x objective with Olympus CK-2 Phase Contrast Microscope. **c.** Flow-cytometry showing double positivity for CM marker cTNT and adult CM marker TNNI3. **d.** TEM images to measure Mito-ER distance in H9C2 cells and respective quantification. Scale bar 0.5 μm. (n=4 biologically independent samples, wilcoxon rank-sum test for p values) **e.** Fluorescent images using Zeiss LSM 710 confocal imaging system of HEK293T expressing Mito marker pECFP-Mito (CFP), DIAPH1-red fluorescent protein, (RFP), and ER marker mEmerald-Calreticulin-N-16 (Emerald), and the overlaid images. Nuclear stained (DAPI stained) HEK293T are shown prior to the overlay images. Scale bar 10 μm. **f.** represents pulldown assay using antibody to DIAPH1 in HiPSCs followed by Western blotting with antibody to MFN1. **g.** qPCR showing *DIAPH1* gene expression normalized either to 18s or *PPIA* in H9C2, HMVECs and BMDMs from WT or global DKO ("gDKO") mice **h-i-j.** Leica SP8 confocal microscopy images of DUOLINK PLA and respective quantification of signal demonstrating DIAPH1-MFN2 interactions in H9C2 cells (**h**), **i.** HMVECs (i) and **j.** BMDMs (j). (**g-j,** n=4 biologically independent samples, unpaired t-test (H9C2 and HMVECs) and wilcoxon rank-sum test(BMDMs) was performed for p values). **k**. Leica SP8 confocal microscopy images of Alexa Fluor™ 488 Phalloidin to stain F-actin (green) and Deoxyribonuclease I, Alexa Fluor™ 594 Conjugate to stain G-actin (red) and corresponding quantification of relative intensity of F/G actin. Scale bar 25 μm. (n=6 biologically independent samples, TukeyHSD test was performed for p value). **l**. represents Leica SP8 confocal microscopy images of DUOLINK PLA signal in HiPSC-CMs either treated with VEH (DMSO) or 10 μM Latrunculin-B (LATB) for 1 h before H/R. (n=4 biologically independent samples, Welch's

ANOVA with t-test pooled SD Test for p value). Data are presented as the mean ± SEM. Source data are provided as a Source Data file.

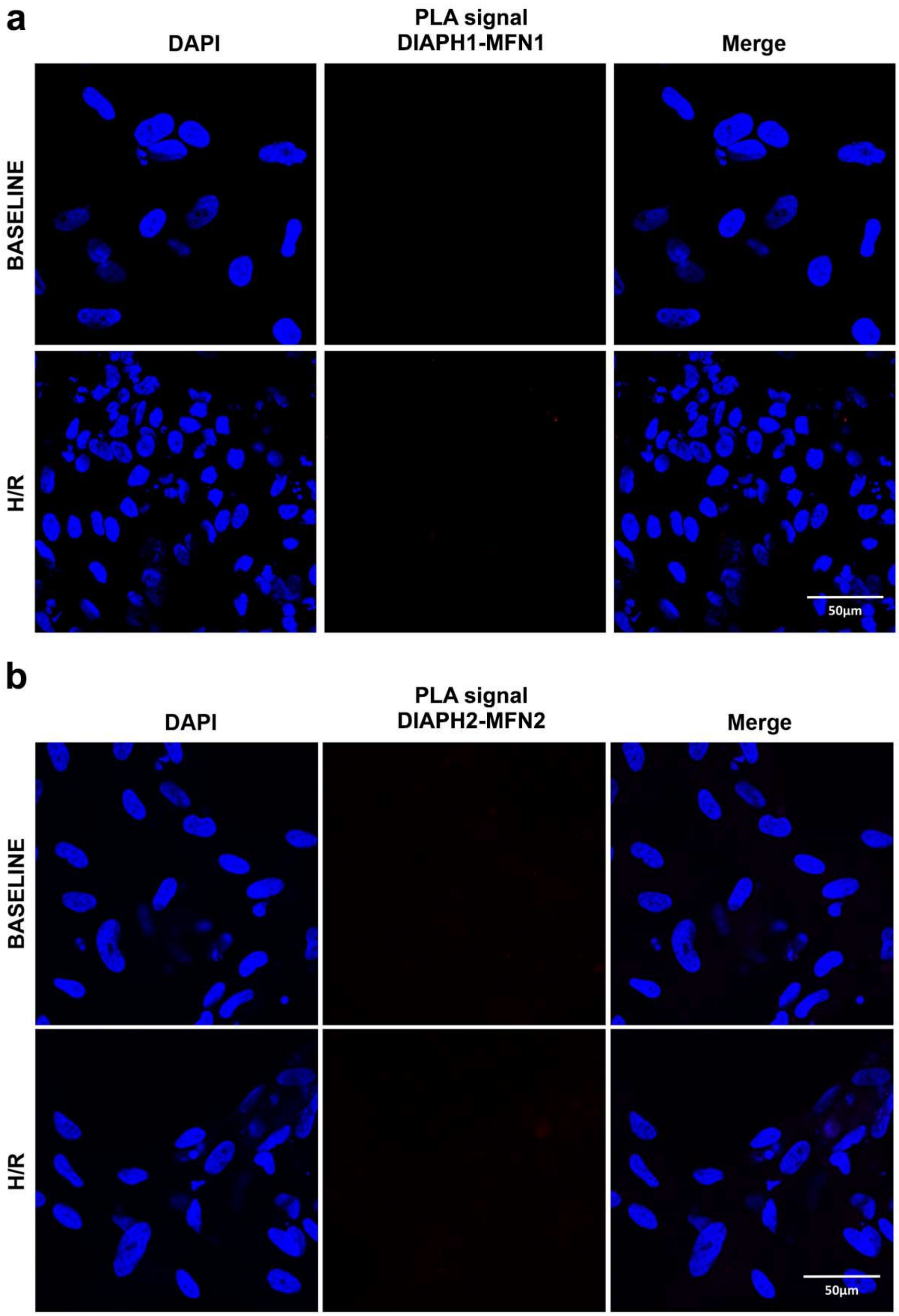

Supplementary figure 2| Data supporting figure 1: a. Leica SP8 confocal microscopy images at 63X magnification representing DUOLINK PLA for DIAPH1-MFN2 under both baseline and H/R conditions, and **b**. DIAPH2-MFN2 interactions in HiPSC-CMs under both baseline and H/R

conditions. (n=4 biologically independent samples). Source data are provided as a Source Data file.

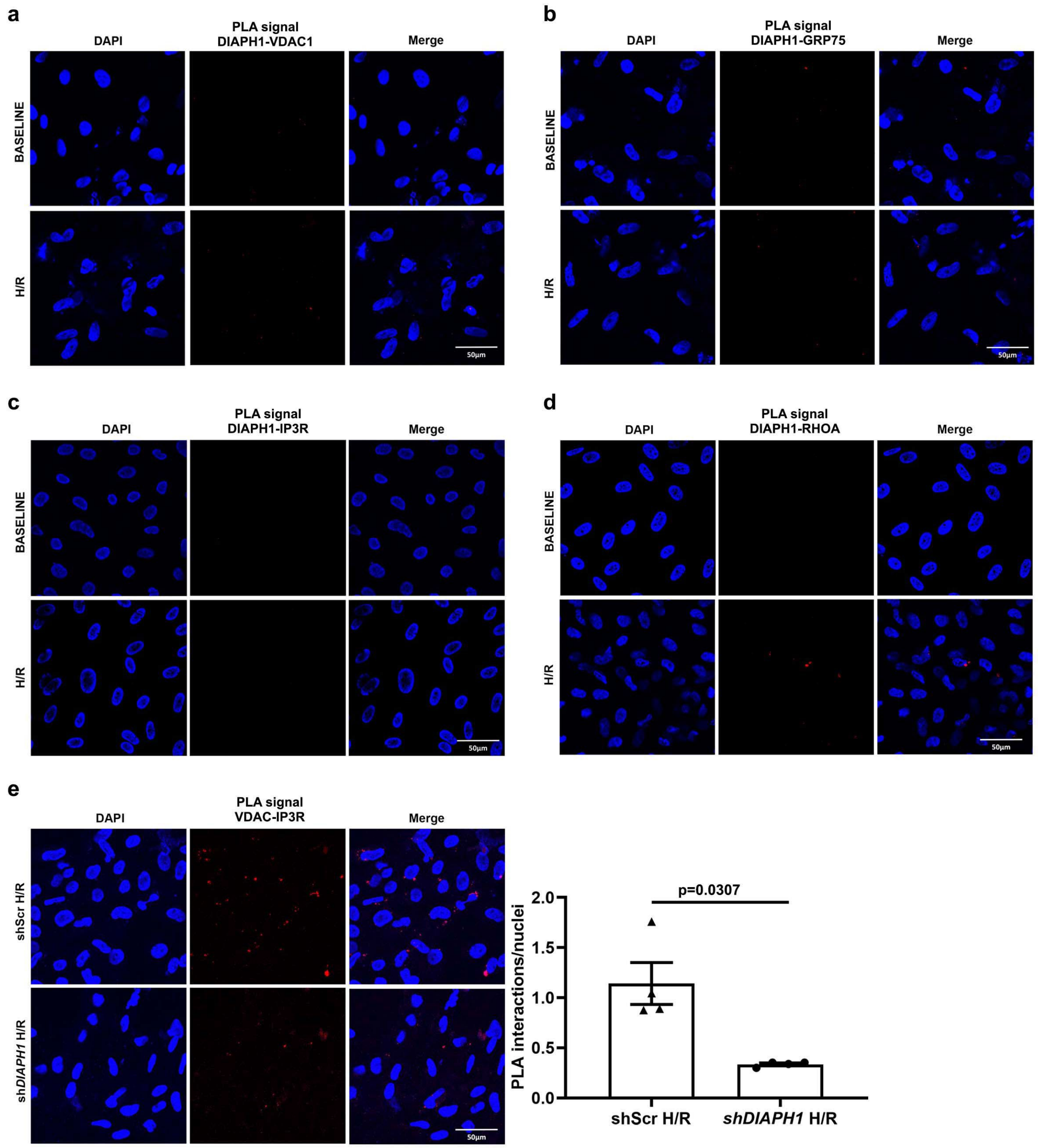

Supplementary figure 3| Data supporting figure 1: a. Leica SP8 confocal microscopy images at 63X magnification representing DUOLINK PLA for DIAPH1-VDAC1, **b.** DIAPH1-GRP75, **c.** DIAPH1-IP3R and **d.** DIAPH1-RHOA interactions in HiPSC-CMs under baseline and H/R conditions. (**a-d**, n=4 biologically independent samples). **e.** represents DUOLINK PLA VDAC-IP3R interactions along with quantification in shScr and sh*DIAPH1* HiPSC-CMs under H/R. (n=4

biologically independent samples, unpaired t test was performed for p value). Data are presented as the mean ± SEM. Source data are provided as a Source Data file.

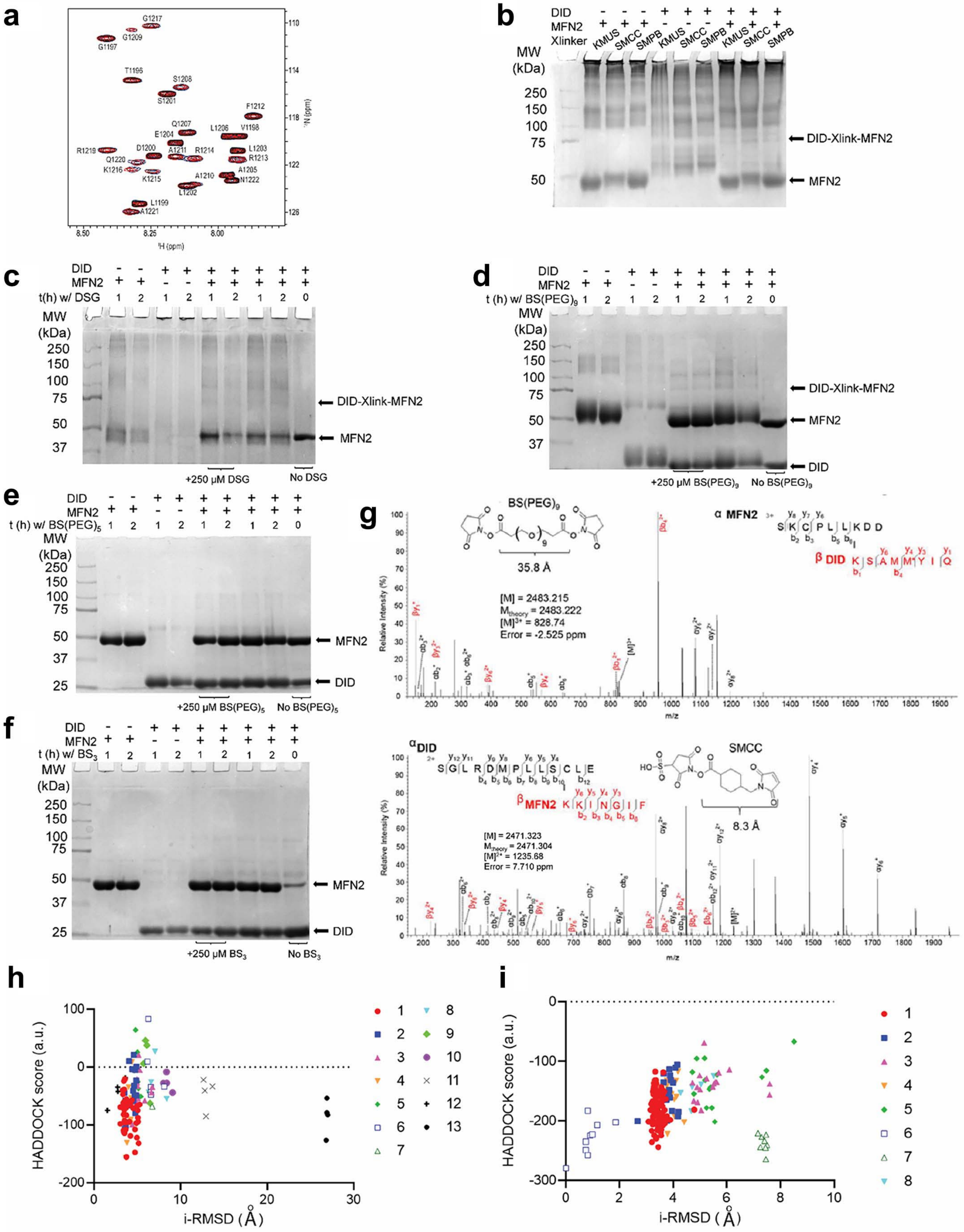
a
G1217
G1209
G1197
T1195
S1208
S1201
F1212
Q1207
L1206
V1198
E1204
R1219
D1200
A1211
R1214
L1203
R1213
Q1220
K1216
K1215
A1210
A1205
N1222
L1202
L1199
A1221
¹H (ppm)
¹⁵N (ppm)
b
DID
MFN2
Xlinker
KMUS
SMCC
SMPB
MW (kDa)
DID-Xlink-MFN2
MFN2
c
t(h) w/ DSG
+250 μM DSG
No DSG
d
t (h) w/ BS(PEG)9
+250 μM BS(PEG)9
No BS(PEG)9
DID
e
t (h) w/ BS(PEG)5
+250 μM BS(PEG)5
No BS(PEG)5
f
t (h) w/ BS3
+250 μM BS3
No BS3
g
BS(PEG)9
35.8 Å
α MFN2
β DID
[M] = 2483.215
Mtheory = 2483.222
[M]3+ = 828.74
Error = -2.525 ppm
Relative Intensity (%)
m/z
αDID
βMFN2
SMCC
8.3 Å
[M] = 2471.323
Mtheory = 2471.304
[M]2+ = 1235.68
Error = 7.710 ppm
h
HADDOCK score (a.u.)
i-RMSD (Å)
i
HADDOCK score (a.u.)
i-RMSD (Å)

Supplementary figure 4 | Data supporting figure 2: DID-MFN2 interactions. **a.** ^{1}H-^{15}N-HSQC spectra of 100 µM [*U*-^{15}N]-DAD^{M1199L} upon titration with 125 µM of MFN2 of 15N DAD^{M1199L} showing no interaction. **b-f.** SDS-PAGE analysis of cross-linked DID and MFN2. DID and/or MFN2 (50 µM) were incubated with cross-linkers (500 µM or 250 µM) for 1-2 h at room temperature. Aliquots of the cross-linking reaction were loaded onto an 8% SDS-polyacrylamide resolving gel. Bands corresponding to the DID monomer, MFN2 monomer, or the cross-linked DID-MFN2 1:1 complex are indicated by black arrows. **b.** Comparison between cross-linking reactions carried out with heterobifunctional (amine-to-sulfhydryl) cross-linkers KMUS, SMCC, and SMPB. Lane 9 shows a distinct band ~75 kDa pertaining to the 1:1 DID-MFN2 complex when SMCC is used. **c-f.** Cross-linking reactions using the homobifunctional (amine-to-amine) cross-linkers, DSG **c**, $BS(PEG)_9$ **d**, $BS(PEG)_5$ **e**, and BS_3 **f**. In **c** and **d**, Lanes 6-9 show a faint band at ~75 kDa corresponding to the 1:1 DID-MFN2 complex. This band is absent in control reactions carried out with DID + cross-linker and MFN2 + cross-linker. In panels **(e)** and **(f)**, this band is also absent in Lanes 6-9. **g.** Representative mass spectra of DID-MFN2 peptides cross-linked using $BS(PEG)_9$ (top) and SMCC (bottom). **h-i**. Distribution of docking solutions based on HADDOCK score and the RMSD of the interfacial residues (i-RMSD). iRMSD is calculated between the average structure in the cluster and the lowest energy structure of the reference [PDB: 6JFK]. **h.** Docking results using both AIRs and UIRs and **i.** Docking results using only UIRs.

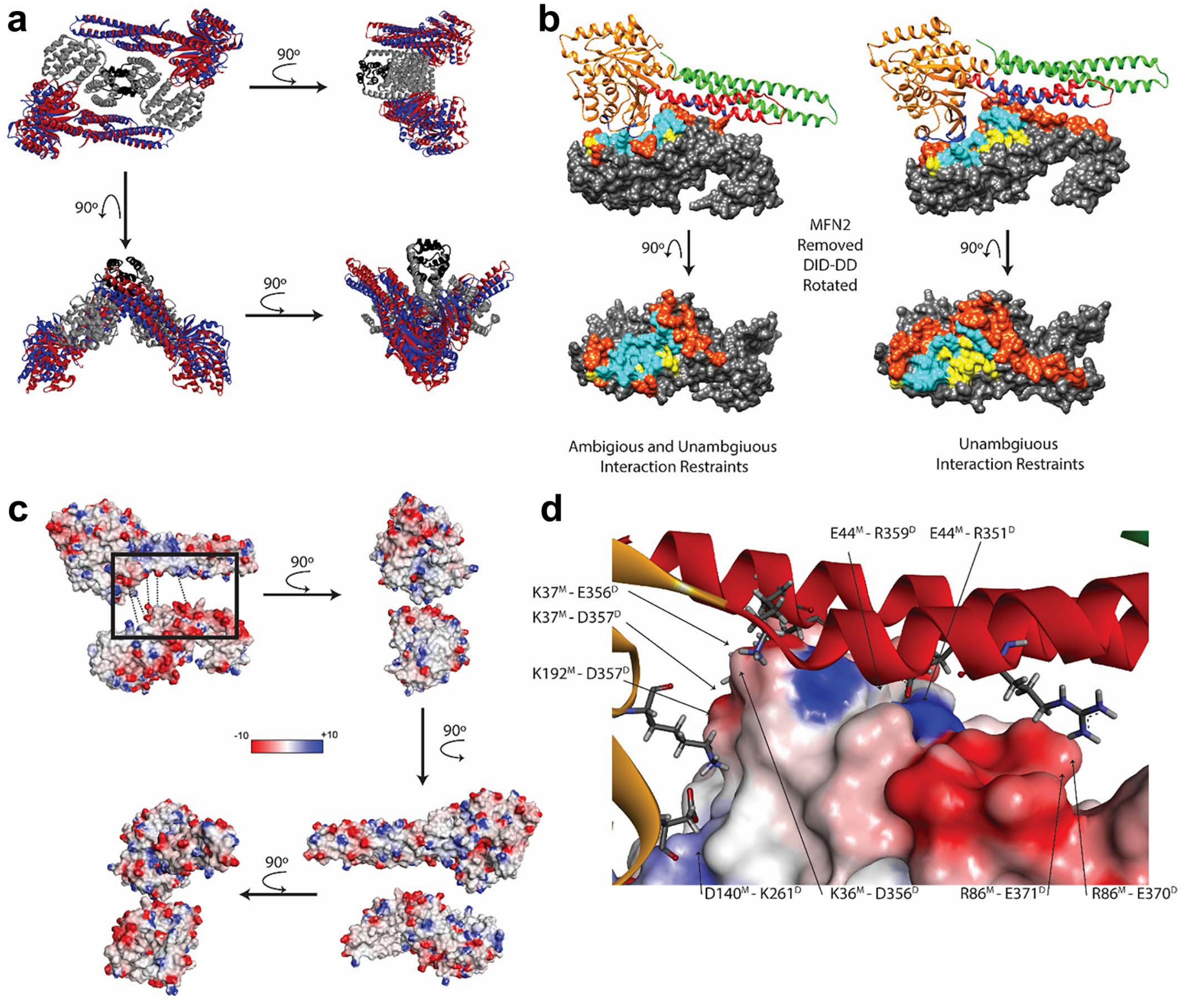

Supplementary figure 5 | Data supporting figure 2: Comparison and analysis of structural models of the DID-DD:MFN2 tetrameric complex using UIRs or both UIRs and AIRs. **a.** Blue and red ribbons represent MFN2 monomers using UIRs and UIRs & AIRs, respectively. Grey and black ribbons represent the DID-DD dimer. **b.** Both docking models show the binding interfaces of DID and MFN2 overlap with the binding interface of DID and DAD. Residues of MFN2 in contact with DID-DD are colored blue. The residues of DID that belong to both the binding interface between DID-DAD and DID-MFN2 are colored cyan. Those that are in the binding interface between DID-DAD exclusively are colored yellow, and those that belong to the

DID-MFN2 binding interface exclusively are colored orange. **c.** The electrostatic maps of MFN2 and DID-DD complex were shown by translating the molecules 50 Å apart from each other to highlight the surface and charge complementarity of the complex. Salt bridges are shown with small dotted lines; the solid box area is depicted in panel **d.** Salt bridges between MFN2 and DID in are labeled with arrows. Residues of MFN2 are labeled with an "M" superscript, and residues of DID-DD are labeled with a "D" superscript.

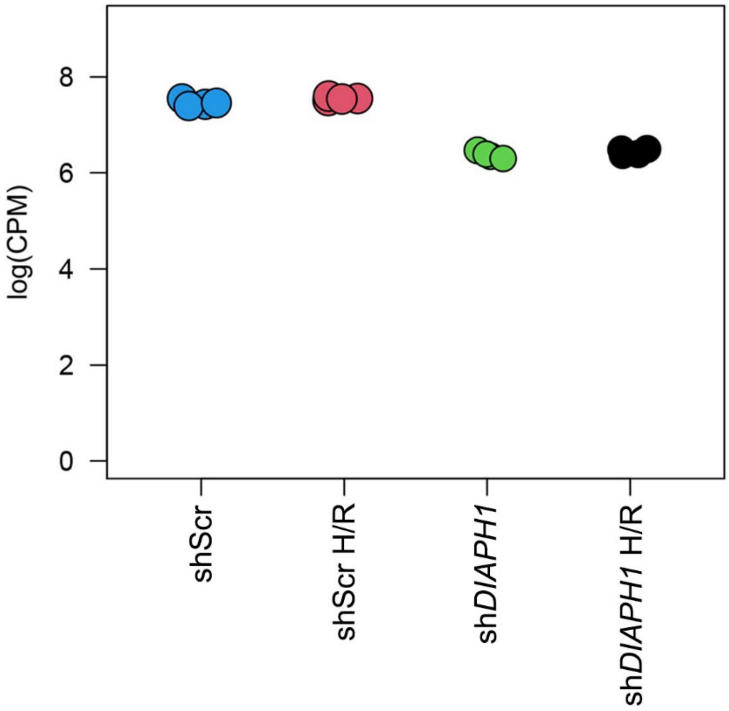

Supplementary figure 6 | Data supporting figure 3: Principle component analysis plot of studies in HiPSCs from bulk RNAseq data and the effects of *DIAPH1* silencing under baseline and H/R conditions. (n=4 biologically independent samples).

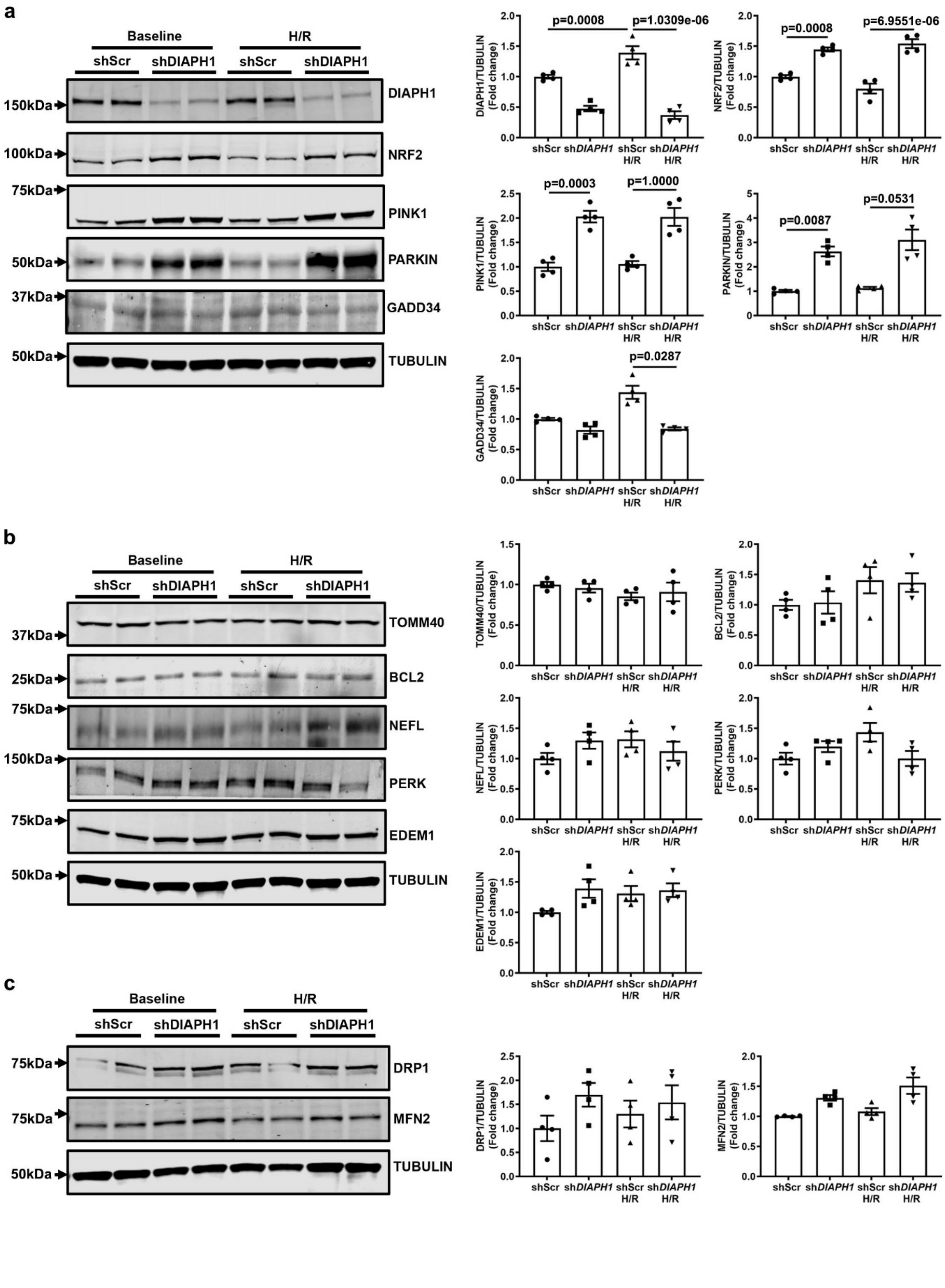

Supplementary figure 7| Data supporting figure 3: a. Western blot analysis was performed in shScr and sh*DIAPH1* HiPSC-CMs under baseline and H/R conditions. **a** represents proteins which

were significantly up- or down-regulated and **b** represents proteins which were not influenced by silencing *DIAPH1* supporting gene expression data from figure 3f. **c.** protein expression data for Mito fission/fusion markers. α-Tubulin was used as loading control to normalize all blots. Quantification was performed using NIH-ImageJ software. (n=4 biologically independent samples. Welch's ANOVA with Games-Howell pairwise comparison Test for PARKIN, GADD34, Krushal-Wallis with Dunn's pairwise comparison test for PERK, EDEM1 and MFN2 and ANOVA with TukeyHSD pairwise comparison for DIAPH1, NRF2, TOMM40, BCL2, NEFL, PINK1, and DRP1for p values). Data are presented as the mean ± SEM. All statistics and source data are provided as a Source Data file.

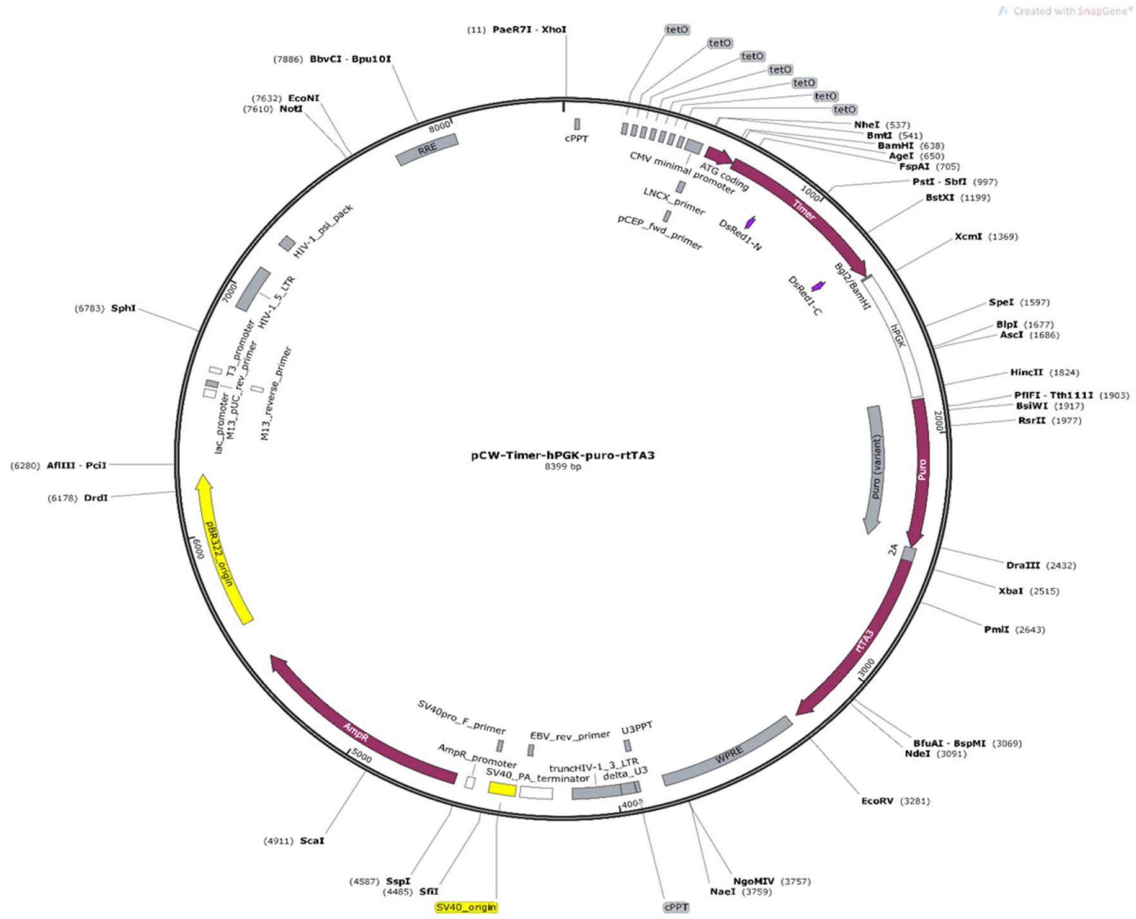

Supplementary figure 8| Data supporting figure 4: Gene map representing integration of both MitoT and doxycycline inducible rtTA3 constructs for efficient transfection.

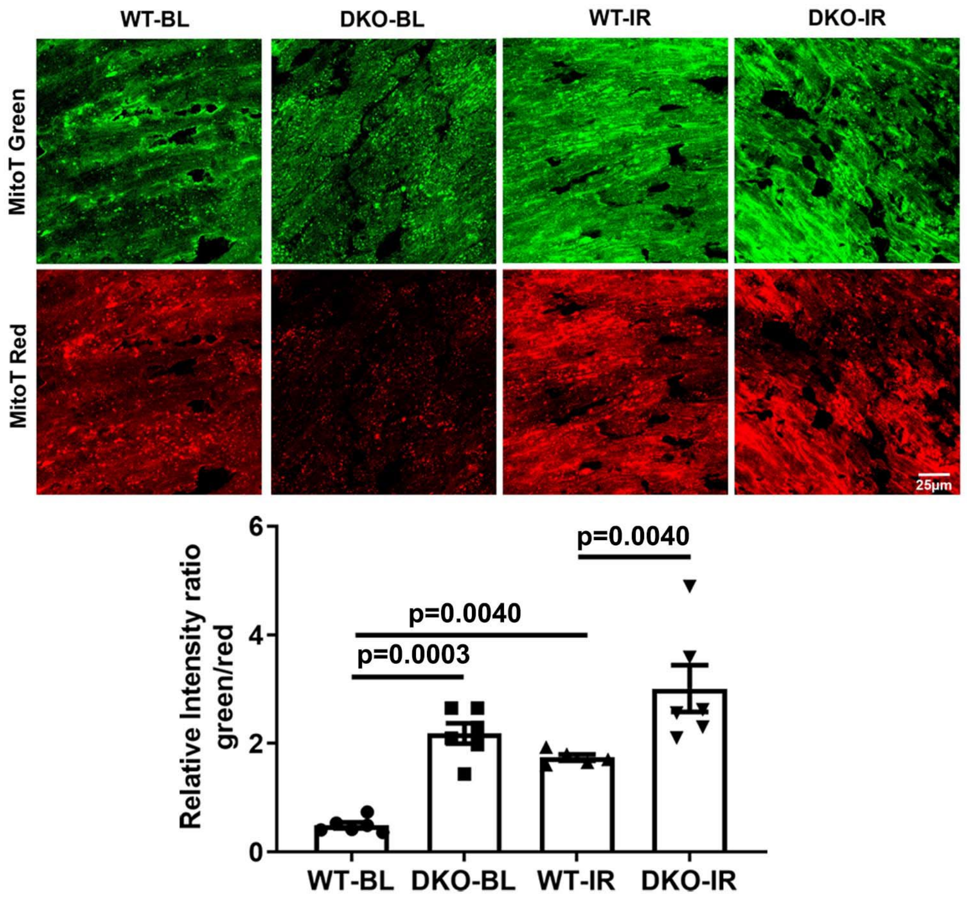

Supplementary figure 9| Data supporting figure 4: isolated perfused mice hearts from WT and global *Diaph1* null (DKO) mice expressing MitoTimer gene. Fluorescence imaging was performed using LEICA SP8 confocal microscope at 63X magnification. Fluorescence signal for green was detected at ex/em range. 488 nm to 530 nm. Laser intensity for 488nm filter was maintained constant at 1.8% and Argon laser power at 20%. Fluorescence signal for red was detected at ex/em. range 561 nm to 590 nm. Laser intensity for 562 nm line was maintained at 2%. Quantification represents ratio of relative intensity of green to red indicating the turnover of Mito.(n=6, n=5 (WT-IR) biologically independent samples, Welch's ANOVA with t-test with pooled SD pairwise comparison test was performed for p value). All statistics and source data are provided as a Source Data file.

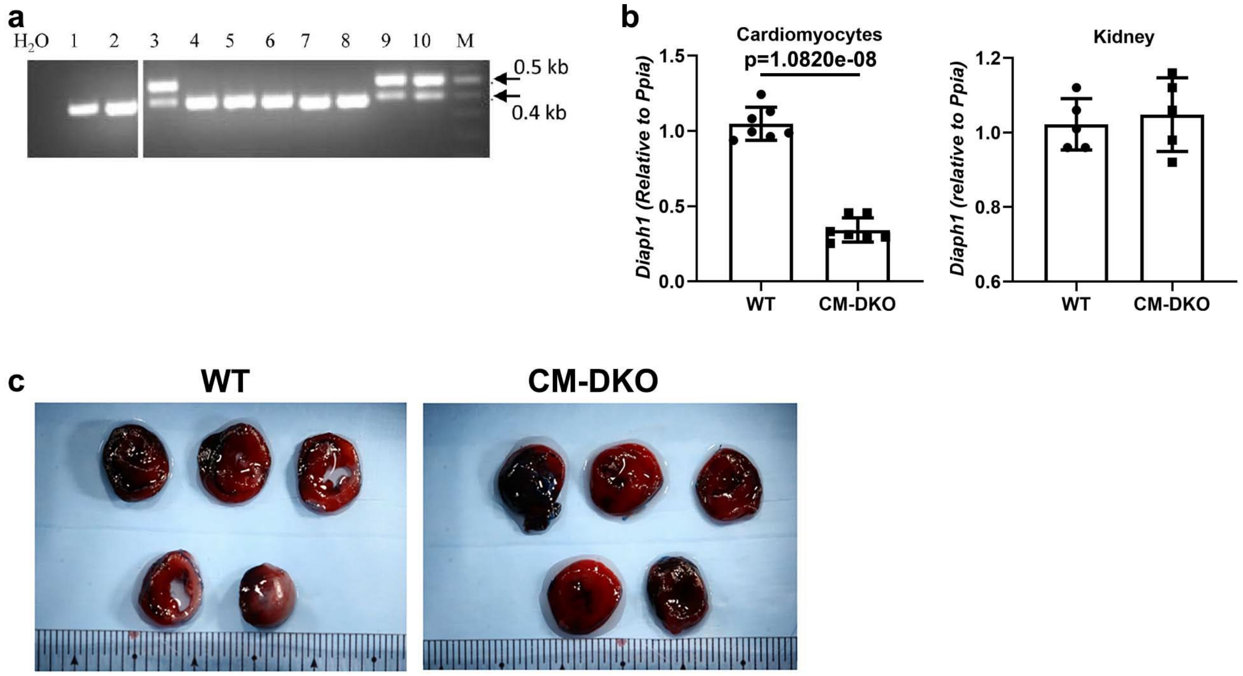

Supplementary figure 10| Data supporting figure 5: a. Detection of the Flp-mediated excision event by PCR. Lane 3, 9 & 10: Heterozygous *Diaph1* floxed mice. Lane 4-8: The WT non-recombined allele. Lane 1 & 2: WT genomic DNA was used as a positive control. PCR without template (H_2O) served as a negative control. M: 1 kb DNA-ladder (NEB). **b.** qPCR on CMs isolated from WT and *Diaph1* floxed mice to measure *Diaph1* silencing, which was not observed in kidney. (n=7 biologically independent samples, unpaired t-test was performed for p value). **c.** represents TTC-stained sections from mice hearts for quantification displayed in figure 5b. Data are presented as the mean ± SEM. All statistics and source data are provided as a Source Data file.

Supplementary Tables

Supplementary Table 1 | Data supporting figure 2. Sequence Comparison between MFN2 Constructs

Full-length human MFN2*, Uniprot ID **O95140**
MSLLFSRCNSIVTVKKNKRHM [22]AEVNASPLKHFVTAKKKINGIFEQLGAYIQESATFLEDT YRNAELDPVTTEEQVLDVKGYLSKVRGISEVLARRHMKVAFFGRTSNGKSTVINAMLWDK VLPSGIGHTTNCFLRVEGTDGHEAFLLTEGSEEKRSAKTVNQLAHALHQDKQLHAGSLVS VMWPNSKCPLLKDDLVLMDSPGIDVTTELDSWIDKFCLDADVFVLVANSESTLMQTEKHF FHKVSERLSRPNIFILNNRWDASASEPEYMEEVRRQHMERCTSFLVDELGVVDRSQAGDR IFFVSAKEVLNARIQKAQGMPEGGGALAEGFQVRMFEFQNFERRFEECISQSAVKTKFEQ HTVRAKQIAEAVRLIMDSLHMAAREQQVYCEEMREERQDR[400] LKFIDKQLELLAQDYKLRIK QITEEVERQVSTAMAEEIRRLSVLVDDYQMDFHPSPVVLKVYKNELHRHIEEGLGRNMSD RCSTAITNSLQTMQQDMIDGLKPLLPVSVRSQIDMLVPRQCFSLNYDLNCDKLCADFQED IEFHFSLGWTMLVNRFLGPKNSRRALMGYNDQVQRPIPLTPANPSMPPLPQGSLTQEEFM VSMVTGLASLTSRTSMGILVVGGVVWKAVGWRLIALSFGLYGLLYVYERLTWTTKAKERA FKRQFVEHASEKLQLVISYTGSNCSHQVQQELSGTFAHLCQQVDV [706]TRENLEQEIAAMNKK IEVLDSLQSKAKLLRNKAGWLDSELNMFTHQYLQPSR[757]
Truncated, modified cytosolic-MFN2*
MGSSHHHHHHSSGLVPRGSHM [22]AEVNASPLKHFVTAKKKINGIFEQLGAYIQESATFLEDT YRNAELDPVTTEEQVLDVKGYLSKVRGISEVLARRHMKVAFFGRTSNGKSTVINAMLWDKV LPSGIGHTTNCFLRVEGTDGHEAFLLTEGSEEKRSAKTVNQLAHALHQDKQLHAGSLVSVMW PNSKCPLLKDDLVLMDSPGIDVTTELDSWIDKFCLDADVFVLVANSESTLMQTEKHFFHKVSE RLSRPNIFILNNRWDASASEPEYMEEVRRQHMERCTSFLVDELGVVDRSQAGDRIFFVSAKEV LNARIQKAQGMPEGGGALAEGFQVRMFEFQNFERRFEECISQSAVKTKFEQHTVRAKQIAEAV RLIMDSLHMAAREQQVYCEEMREERQDR[400] [706]TRENLEQEIAAMNKKIEVLDSLQSKAKLLRNKAGWLDSELNMFTHQYLQPSR[757]

* MFN2 domains HD1, GTPase, and HD2 are colored in red, orange, and green, respectively. Residues highlighted in yellow are present in the construct used by Li *et al. Nat Commun* 10, 4914 (2019). https://doi.org/10.1038/s41467-019-12912-0, [PDB entry 6JFK].

Supplementary Table 2 | Data supporting figure 2. Intermolecular cross-links between DID and MFN2

Cross-linker reagent	DSG	SMCC		
Spacer length	7.7 Å	8.3 Å		
Specificity	Homobifunctional<br>Reactive toward primary amines	Heterobifunctional<br>Forms a thio-ether bond between a primary amine and a sulfhydryl group		
Sequences of cross-linked peptides	^{369}GEEDSYDL**K**G^{378}-^{74}QVLDV**K**GY^{81}	^{221}LKAFMNN**K**F^{229}-^{131}N**C**FLRVE^{137}	^{154}SGLRDMPLLS**C**LE^{166}-^{37}**K**KINGIF^{43}	^{158}**K**TVNQLAHALH^{168}-^{158}DMPLLS**C**L1^{65}
Cross-linked residues	DID K377 – MFN2 K79	DID K228 – MFN2 C132	DID C164 – MFN2 K37	MFN2 K158-DID C164
$M_{theoretical}$	2128.992 Da	2211.109 Da	2471.304 Da	2341.204 Da
$[M]_{experimental}$	2128.976 Da	2211.107 Da	2471.323 Da	2341.195 Da
Error	-7.3 ppm	-0.86 ppm	7.7 ppm	-4.0 ppm

Cross-linker reagent	BS(PEG)$_9$				
Spacer length	35.8 Å				
Specificity	Homobifunctional<br>Reactive toward primary amines				
Sequences of cross-linked peptides	^{186}SKCPLL**K**DD^{194}-^{143}**K**SAMM*YIQ^{150}<br>M* - oxidized Methionine	^{103}GRTSNG**K**STV^{112}-^{311}**K**VGCLQL^{317}	^{305}SA**K**EVLNA^{312}-^{225}MNN**K**FGIK^{232}	^{350}SQSAV**K**T^{356}-^{300}DGL**K**SGTTIAL K^{311}	^{727}LQS**K**AK^{732}-^{307}TIAL**K**VGC^{314}-
Cross-linked residues	MFN2 K192 – DID K143	MFN2 K109 – DID K311	MFN2 K307 – DID K228	MFN2 K355 – DID K303	MFN2 730 – DID K311
$M_{theoretical}$	2483.222 Da	2244.200	2260.240	2401.317	1956.118
$[M]_{experimental}$	2483.215 Da	2244.177	2260.200	2401.358	1956.085
Error	-2.5 ppm	-10 ppm	16 ppm	17 ppm	-17 ppm

Supplementary Table 3| Data supporting figure 2. Active residues on MFN2 and DID-DD used to define ambiguous interaction restraints

Active Residues on each MFN2 monomer (positive cross-links with DID)	Active Residues on each DID monomer (within 5 Å of DAD in the model of a DID-DAD complex)
37, 79, 109, 132, 158, 192, 307, 355, 730	177, 178, 222, 225, 226,227, 231, 261, 262, 264, 265, 266, 268, 29, 316, 319, 320, 323, 357, 360, 361, 366

Supplementary Table 4 | Data supporting figure 2. Distance violations in the representative model of the docked A_2B_2 DID-DD-MFN2 complex

Atom pairs	Expected distances	Observed distances	Violations at or below 5 Å	Violations above 5 Å
N^{ε} of Lys 143 (DID-DD, seg ID B) and N^{ε} of Lys 192 (MFN2, seg ID A)	35.8 Å	38.7 Å	2.9 Å	-
N^{ε} of Lys 1143 (DID-DD, seg ID B) and N^{ε} of Lys 2192 (MFN2, seg ID C)	35.8 Å	36.4 Å	0.6 Å	-
N^{ε} of Lys 109 (MFN2, seg ID A) and N^{ε} of Lys 311 (DID-DD, seg ID B)	35.8 Å	40.9 Å	-	5.1 Å
N^{ε} of Lys 2109 (MFN2, seg ID C) and N^{ε} of Lys 1311 (DID-DD, seg ID B)	35.8 Å	40.9 Å	-	5.1 Å
N^{ε} of Lys 307 (MFN2, seg ID A) and N^{ε} of Lys 228 (DID-DD, seg ID B)	35.8 Å	30.7 Å	-	-5.1 Å
N^{ε} of Lys 2307 (MFN2, seg ID C) and N^{ε} of Lys 1228 (DID-DD, seg ID B)	35.8 Å	30.9 Å	-4.9 Å	-
N^{ε} of Lys 355 (MFN2, seg ID A) and N^{ε} of Lys 303 (DID-DD, seg ID B)	35.8 Å	40.8 Å	5.0 Å	-
N^{ε} of Lys 2355 (MFN2, seg ID C) and N^{ε} of Lys 1303 (DID-DD, seg ID B)	35.8 Å	40.7 Å	4.9 Å	-
N^{ε} of Lys 730 (MFN2, seg ID A) and N^{ε} of Lys 311 (DID-DD, seg ID B)	35.8 Å	30.9 Å	-4.9 Å	-
N^{ε} of Lys 2730 (MFN2, seg ID C) and N^{ε} of Lys 1311 (DID-DD, seg ID B)	35.8 Å	30.8 Å	-5.0 Å	-
N^{ε} of Lys 79 (MFN2, seg ID A) and N^{ε} of Lys 377 (DID-DD, seg ID B)	7.7 Å	9.1 Å	1.4 Å	-
N^{ε} of Lys 2079 (MFN2, seg ID C) and N^{ε} of Lys 1377 (DID-DD, seg ID B)	7.7 Å	8.4 Å	0.7 Å	-
N^{ε} of Lys 158 (MFN2, seg ID A) and S^{γ} of Cys 164 (DID-DD, seg ID B)	8.3 Å	17.7 Å	-	9.4 Å
N^{ε} of Lys 2158 (MFN2, seg ID C) and S^{γ} of Cys 1164 (DID-DD, seg ID B)	8.3 Å	19.1 Å	-	10.8 Å
N^{ε} of Lys 37 (MFN2, seg ID A) and S^{γ} of Cys 164 (DID-DD, seg ID B)	8.3 Å	37.4 Å	-	29.1 Å
N^{ε} of Lys 2037 (MFN2, seg ID C) and S^{γ} of Cys 1164 (DID-DD, seg ID B)	8.3 Å	35.6 Å	-	27.3 Å
S^{γ} of Cys 132 (MFN2, seg ID A) and N^{ε} of Lys 228 (DID-DD, seg ID B)	8.3 Å	15.8 Å	-	7.5 Å
S^{γ} of Cys 2132 (MFN2, seg ID C) and N^{ε} of Lys 1228 (DID-DD, seg ID B)	8.3 Å	17.2 Å	-	8.9 Å

Supplementary Table 5| Data supporting figure 2. Hydrogen bonds and salt bridges in the representative model of the docked A_2B_2 DID-DD-MFN2 complex

Hydrogen bonds	Salt bridges
HZ2 of Lys 36 (MFN2) and OE1 of Glu 356 (DID-DD)	NZ of Lys 36 (MFN2) and OE1 of Glu 356 (DID-DD)
HZ2 of Lys 37 (MFN2) and OD1 of Asn 355 (DID-DD)	NZ of Lys 37 (MFN2) and OD1 of Asp 357 (DID-DD)
O of Lys 37 (MFN2) and HH12 of Arg 359 (DID-DD)	NZ of Lys 38 (MFN2) and OE1 of Glu 354 (DID-DD)
O of Lys 37 (MFN2) and HH22 of Arg 359 (DID-DD)	NZ of Lys 38 (MFN2) and OD2 of Asp 357 (DID-DD)
HZ2 of Lys 38 (MFN2) and OD2 of Asp 357 (DID-DD)	OE1 of Glu 44 (MFN2) and NE of Arg 351 (DID-DD)
HZ3 of Lys 38 (MFN2) and OD2 of Asp 357 (DID-DD)	OE1 of Glu 44 (MFN2) and NH1 of Arg 351 (DID-DD)
HZ2 of Lys 38 (MFN2) and OE1 of Glu 354 (DID-DD)	OE2 of Glu 44 (MFN2) and NH1 of Arg 359 (DID-DD)
OE1 of Glu 44 (MFN2) and HH11 of Arg 351 (DID-DD)	NH1 of Arg 86 (MFN2) and OE2 of Glu 370 (DID-DD)
HH11 of Arg 86 (MFN2) and OE2 of Glu 370 (DID-DD)	NH2 of Arg 86 (MFN2) and OE2 of Glu 371 (DID-DD)
OE1 of Glu 90 (MFN2) and HD22 of Asn 363 (DID-DD)	OD1 of Asp 140 (MFN2) and NZ of Lys 261 (DID-DD)
O of Asp 140 (MFN2) and HZ2 of Lys 261 (DID-DD)	NZ of Lys 192 (MFN2) and OD1 of Asp 357 (DID-DD)
O of Asp 140 (MFN2) and HZ3 of Lys 261 (DID-DD)	
OD1 of Asp 140 (MFN2) and HZ3 of Lys 261 (DID-DD)	
OD1 of Asp 140 (MFN2) and HZ2 of Lys 261 (DID-DD)	
OD1 of Asp 140 (MFN2) and HE21 of Gln 316 (DID-DD)	
O of Gly 141 (MFN2) and HZ3 of Lys 261 (DID-DD)	
ND1 of His 142 (MFN2) and O of Lys 261 (DID-DD)	
HE2 of His 142 (MFN2) and O of Met 135 (DID-DD)	
HZ2 of Lys 158 (MFN2) and O of Leu 172 (DID-DD)	
HZ1 of Lys 158 (MFN2) and OD1 of Ala 123 (DID-DD)	
HZ1 of Lys 158 (MFN2) and OD1 of Asn 226 (DID-DD)	
HZ3 of Lys 158 (MFN2) and OD1 of Asn 226 (DID-DD)	

Supplementary Table 6 | Data supporting figure 2. HADDOCK Parameters

Parameter	Value
Structure definition	Molecule 1, Segment ID A: first MFN2 monomer (residue numbers 24-756)*<br>Molecule 2, Segment ID B: DID-DD dimer (residue numbers 142-444 on the first monomer and 1142-1444 on the second monomer)*<br>Molecule 3, Segment ID C: second MFN2 monomer (residue numbers 2024-2756)*<br>*Numbering on the second monomer corresponds to the value from the first monomer incremented by 1000 (for DID) or 2000 (for MFN2) to distinguish between monomers during docking
Histidine protonation states	Automatically defined (default)
Semi-flexible and fully-flexible segments	Automatically defined (default)
Molecular interaction matrix	Between Molecule 1 and Molecule 1: 1.0<br>Between Molecule 1 and Molecule 2: 1.0<br>Between Molecule 1 and Molecule 3: 1.0<br>Between Molecule 2 and Molecule 1: 1.0<br>Between Molecule 2 and Molecule 2: 1.0<br>Between Molecule 2 and Molecule 3: 1.0<br>Between Molecule 3 and Molecule 1: 1.0<br>Between Molecule 3 and Molecule 2: 1.0<br>Between Molecule 3 and Molecule 3: 1.0
Distance restraints	Unambiguous restraints:<br>assign (resid 192 and segid A and name NZ) (resid 143 and segid B and name NZ) 35.8 5.0 5.0<br>assign (resid 109 and segid A and name NZ) (resid 311 and segid B and name NZ) 35.8 5.0 5.0<br>assign (resid 307 and segid A and name NZ) (resid 228 and segid B and name NZ) 35.8 5.0 5.0<br>assign (resid 355 and segid A and name NZ) (resid 303 and segid B and name NZ) 35.8 5.0 5.0<br>assign (resid 730 and segid A and name NZ) (resid 311 and segid B and name NZ) 35.8 5.0 5.0<br>assign (resid 79 and segid A and name NZ) (resid 377 and segid B and name NZ) 7.7 5.0 5.0<br>assign (resid 158 and segid A and name NZ) (resid 164 and segid B and name SG) 8.3 5.0 5.0<br>assign (resid 37 and segid A and name NZ) (resid 164 and segid B and name SG) 8.3 5.0 5.0<br>assign (resid 132 and segid A and name SG) (resid 228 and segid B and name NZ) 8.3 5.0 5.0<br>assign (resid 2192 and segid C and name NZ) (resid 1143 and segid B and name NZ) 35.8 5.0 5.0<br>assign (resid 2109 and segid C and name NZ) (resid 1311 and segid B and name NZ) 35.8 5.0 5.0<br>assign (resid 2307 and segid C and name NZ) (resid 1228 and segid B and name NZ) 35.8 5.0 5.0

	assign (resid 2355 and segid C and name NZ) (resid 1303 and segid B and name NZ) 35.8 5.0 5.0<br>assign (resid 2730 and segid C and name NZ) (resid 1311 and segid B and name NZ) 35.8 5.0 5.0<br>assign (resid 2079 and segid C and name NZ) (resid 1377 and segid B and name NZ) 7.7 5.0 5.0<br>assign (resid 2158 and segid C and name NZ) (resid 1164 and segid B and name SG) 8.3 5.0 5.0<br>assign (resid 2037 and segid C and name NZ) (resid 1164 and segid B and name SG) 8.3 5.0 5.0<br>assign (resid 2132 and segid C and name SG) (resid 1228 and segid B and name NZ) 8.3 5.0 5.0<br><br>Non-polar hydrogens removed during docking (default)<br>Random exclusion of a fraction of AIRs enabled, with 2 partitions for random exclusion (default)
Sampling parameters (default)	Number of structures for rigid docking: 1000<br>Number of trials for rigid body minimization: 5<br>Sample 180 degrees rotated solutions during rigid body EM: Enabled<br>Number of structures for semi-flexible refinement: 200<br>Solvent used for last iteration: water<br>Number of structures for explicit solvent refinement: 200<br>Electrostatic energy term, Epsilon: 10.0
Parameters for clustering (default)	Clustering method – Fraction of Common Contacts (FCC)<br>RMSD Cutoff for clustering: 0.6 Angstrom<br>Minimum cluster size: 4
Dihedral and hydrogen bond restraints, residual dipolar couplings, pseudo contact shift restraints, relaxation anisotropy restraints	None
Noncrystallographic symmetry restraints	None
Symmetry Restraints	Enabled, with force constant of 10.0<br>C2 Symmetry Pairs:<br>First C2 pair: Seg ID B residues 142-444 and seg ID B residues 1142-1444<br>Second C2 pair: Seg ID A residues 24-756 and seg ID C residues 2024-2756
Energy constants for unambiguous restraints and ambiguous restraints (default)<br>Iterations<br>it0: Rigid body EM<br>it1: Semi-flexible SA<br>it2: Water refinement	First iteration: 0<br>Last iteration: 2<br>Energy constants per stage<br>Hot: 10.0<br>Cool1: 10.0<br>Cool2: 50.0<br>Cool3: 50.0

Energy and interaction parameters (default)	Nonbonded parameters: OPLSX, dihedral angle restraints included<br>Electrostatic energy terms included during rigid body docking and semi-flexible SA<br>Constant dielectric (cdie) electrostatic energy term<br>Scaling of intermolecular interactions for rigid body EM: 1.0<br>Scaling of intermolecular interactions for semi-flexible SA (it1): initial values of 0.001 for both rigid body dynamic stage and cool2, 0.05 for cool3; final values of 0.001 for rigid body dynamic stage and 1.0 for both cool2 and cool3
Scoring parameters	Default values for it0, it1, and water refinement<br>Evdw: 0.01, 1.0, 1.0<br>Eelec: 1.0, 1.0, 0.2<br>Eair: 0.01, 0.1, 0.1<br>Esym: 0.1,0.1,0.1<br>Edesolv: 1.0,1.0,1.0<br>dEint: 0.0, 0.0, 0.0

Supplementary Table 7| Data supporting figure 2. Statistics for the most reliable clusters of docking solutions obtained using different docking protocols

Type of interaction restraints	AIRs (DID residues bound to DAD were defined as active residues) Unambiguous interaction restraints (from positive cross-links between DID and MFN2)	Unambiguous interaction restraints (from positive cross-links between DID and MFN2)
Most reliable cluster	Cluster 1	Cluster 6
HADDOCK score (a.u.)	-139.0 ± 13.1	-255.0 ±16.1
Cluster size	47	9
RMSD from the overall lowest-energy structure ($kcal \cdot mol^{-1}$)	6.0 ± 0.6	0.6 ± 0.3
Van der Waals energy ($kcal \cdot mol^{-1}$)	-122.6 ± 9.6	-154.5 ± 12.9
Electrostatic energy ($kcal \cdot mol^{-1}$)	-1508.8 ± 109.8	-1009.1 ± 30.3
Desolvation energy ($kcal \cdot mol^{-1}$)	23.1 ± 17.6	56.4 ± 13.3
Restraints violation energy ($kcal \cdot mol^{-1}$)	1707.3 ± 164.01	433.4 ± 31.21
Buried Surface Area ($Å^2$)	5453.2 ± 340.4	5429.8 ± 147.1
Z-Score	-1.9	-1.4

Supplementary Table 8: Clone IDs (Materials and Methods)

Clone ID	Knockdown Gene	Target Sequence
TRCN0000118678	*DIAPH1*	GCCCAGAATCTCTCAATCTTT
TRCN00000626660	*AGER*	GCGGCTGGAATGGAAACTGAA
SHC002V	Controls	Non-Mammalian

Supplementary Table 9: Primers used in the studies (Materials and Methods)

**Taqman Catalog #**	**Human Gene**
Hs00946556_m1	*DIAPH1*
Hs00762436_s1	*TOMM40*
Hs00975961_g1	*NRF2*
Hs01048932_g1	*BCL2*
Hs00196245_m1	*NEFL*
Hs01038322_m1	*PARKIN*
Hs00984003_m1	*PERK*
Hs00169585_m1	*GADD34*
Hs00976004_m1	*EDEM1*
Hs04194521_s1	*PPIA*
**Taqman Catalog #**	**Rat Gene**
Rn01406955_m1	*Diaph1*
Rn00690933_m1	*Ppia*
**Taqman Catalog #**	**Mouse Gene**
Mm00438700_m1	*Diaph1*
Mm02342430_g1	*Ppia*

Supplementary Table 10: Primary antibodies used for DUOLINK PLA and Western blotting studies

Antibodies for PLA	Host	Company	Cat #	Dilution
DIAPH1	Rabbit	ABCAM	ab129167	1:200
DIAPH2	Rabbit	ABCAM	ab181165	1:200
MFN2	Mouse	ABCAM	ab56889	1:150
MFN1	Mouse	SIGMA	WH0055669M4	1:200
VDAC1	Mouse	ABCAM	ab186321	1:200
GRP75	Mouse	ABCAM	ab2799	1:200
IP3R	Mouse	ABCAM	ab252536	1:200
IP3R1	Rabbit	Cell Signaling	8568	1:200
RHOA	Mouse	ABCAM	ab54835	1:200
**Antibodies for WB**	**Host**	**Company**	**Cat #**	
DIAPH1	Rabbit	ABCAM	ab129167	1:1000
MFN2	Mouse	ABCAM	ab56889	1:1000
NRF2	Rabbit	ABCAM	ab137550	1:1000
PINK1	Rabbit	Cell Signaling	6946S	1:1000
PARKIN	Mouse	Cell Signaling	4211S	1:1000
GADD34	Mouse	ABCAM	ab236516	1:1000
α-TUBULIN	Mouse	SIGMA	T5168	1:25000
TOMM40	Rabbit	ABCAM	ab185543	1:1000
BCL2	Rabbit	ABCAM	ab32124	1:1000
NEFL	Rabbit	Novus Biologicals	NB300-131	1:1000
PERK	Mouse	Cell Signaling	3192S	1:1000
EDEM1	Rabbit	ABCAM	ab200645	1:1000
DRP1	Rabbit	ABCAM	ab184247	1:1000
