# Supplementary material for: DIAPH1-MFN2 interaction regulates mitochondria-SR/ER contact and modulates ischemic/hypoxic stress
Source: Nat Commun. 2023 Oct 30;14:6900. doi: 10.1038/s41467-023-42521-x (PMC10616211; doi:10.1038/s41467-023-42521-x)
Supplement: Supplementary file 2 — Description of Additional Supplementary Files [file 41467_2023_42521_MOESM2_ESM.pdf]

File name: Supplementary Movie 1

Description: Video represents beating of CMs differentiated from HiPSCs at day 24 using 10x objective with Olympus CK-2 Phase Contrast Microscope.

File name: Supplementary Movie 2

Description: Video represents beating of CMs differentiated from HiPSCs at day 36 using 20x objective with Olympus CK-2 Phase Contrast Microscope.
