# Supplementary material for: DIAPH1-MFN2 interaction regulates mitochondria-SR/ER contact and modulates ischemic/hypoxic stress
Source: Nat Commun. 2023 Oct 30;14:6900. doi: 10.1038/s41467-023-42521-x (PMC10616211; doi:10.1038/s41467-023-42521-x)
Supplement: Supplementary file 5 — Reporting Summary [file 41467_2023_42521_MOESM5_ESM.pdf]

nature portfolio

Corresponding author(s): Ravichandran Ramasamy

Last updated by author(s): Sep 29, 2023

Reporting Summary

Nature Portfolio wishes to improve the reproducibility of the work that we publish. This form provides structure for consistency and transparency in reporting. For further information on Nature Portfolio policies, see our Editorial Policies and the Editorial Policy Checklist.

Statistics

For all statistical analyses, confirm that the following items are present in the figure legend, table legend, main text, or Methods section.

n/a	Confirmed	
☐	☒	The exact sample size (*n*) for each experimental group/condition, given as a discrete number and unit of measurement
☐	☒	A statement on whether measurements were taken from distinct samples or whether the same sample was measured repeatedly
☐	☒	The statistical test(s) used AND whether they are one- or two-sided<br>*Only common tests should be described solely by name; describe more complex techniques in the Methods section.*
☒	☐	A description of all covariates tested
☐	☒	A description of any assumptions or corrections, such as tests of normality and adjustment for multiple comparisons
☐	☒	A full description of the statistical parameters including central tendency (e.g. means) or other basic estimates (e.g. regression coefficient) AND variation (e.g. standard deviation) or associated estimates of uncertainty (e.g. confidence intervals)
☐	☒	For null hypothesis testing, the test statistic (e.g. *F*, *t*, *r*) with confidence intervals, effect sizes, degrees of freedom and *P* value noted<br>*Give P values as exact values whenever suitable.*
☒	☐	For Bayesian analysis, information on the choice of priors and Markov chain Monte Carlo settings
☒	☐	For hierarchical and complex designs, identification of the appropriate level for tests and full reporting of outcomes
☒	☐	Estimates of effect sizes (e.g. Cohen's *d*, Pearson's *r*), indicating how they were calculated

Our web collection on statistics for biologists contains articles on many of the points above.

Software and code

Policy information about availability of computer code

Data collection	NanoString multiplex detection of gene expression was performed using nCounter technology by NanoString<br>Colorimetric/Florometric data was collected using TECAN Infinity Pro 200 plate reader<br>Confocal microscopy imaging was performed using LEICA SP8 confocal microscope and all other image collection was performed using EVOS XL Core Imaging System<br>OCR measurements for mitochondrial stress were collected using Seahorse XFp analyzer<br>For Electron microscopy, images were obtained using Philips CM12 TEM (Philips) transmission electron microscope, photographed using a Gatan 4k x 2.7k digital camera (Gatan Inc.).<br>Echocardiography was performed using Vevo 2100 high-resolution ultrasound imaging system (Visual Sonics Inc. Toronto, Ontario, Canada)<br>Odyssey (Li-COR) Infrared Imaging System was used to collect the western blots images<br>LSRII UV (BD Biosciences) cell analyzer and BD FACSDiva software was used to collect the flow cytometry data<br>For structural studies NMR spectra were recorded using a Bruker Avance II spectrometer with a proton Larmor frequency of 700 MHz and equipped with a TXI cryoprobe. All spectra were processed using Topspin 2.1 (Bruker).<br>For mass spectrometric studies, Thermo Scientific Orbitrap Velos mass spectrometer operating with an emitter voltage at 4.5 kV and ion transfer tube temperature at 275*C was used. The Velos was operated in a data dependent acquisition mode where multiple charged ions with abundance > 6000 cps were selected for the MS/MS fragmentation.
Data analysis	All microscopy images were analyzed using NIH-ImageJ/Fiji distribution of ImageJ 2.0.0 software.<br>Graphs plots were generated using Graph Pad Prism 9.<br>RNASeq Analysis was performed as follows.<br>Fastq files were aligned to the mouse genome with Rsubread.<br>Gene expression was quantified with featureCounts.

Differential expression analysis was performed with Limma 3.38.3
Pathway Analysis was performed with iPathwayguide and WebGestalt
Hierarchical clustering was performed using Cluster 3.0.
Dendrograms and heatmaps were displayed using JavaTreeview.
All NMR spectra were processed using Topspin 2.1 (Bruker).
DIAPH1-MFN2 complex is a structural model obtained by the HADDOCK docking program
Seahorse XFp analysis was performed using Wave Version 2.6.0.31 software
Measurement of infarct area was performed using AxioVision software
Echocardiography analysis was performed using Vevo 2100 software.
Mass spectrometric data analysis was performed using the system software pLink version 2.

For manuscripts utilizing custom algorithms or software that are central to the research but not yet described in published literature, software must be made available to editors and reviewers. We strongly encourage code deposition in a community repository (e.g. GitHub). See the Nature Portfolio guidelines for submitting code & software for further information.

Data

Policy information about availability of data

All manuscripts must include a data availability statement. This statement should provide the following information, where applicable:

- Accession codes, unique identifiers, or web links for publicly available datasets
- A description of any restrictions on data availability
- For clinical datasets or third party data, please ensure that the statement adheres to our policy

The authors declare that the data supporting the findings of this study are available within the paper and its Supplementary Information. Human iPSC derived CM raw and aligned sequencing data, resulting raw and normalized count data, and supporting sample data are publicly available through NCBI GEO database with accession number: GSE214149 (https://www.ncbi.nlm.nih.gov/geo/query/acc.cgi) . All biological materials used in this study are available as indicated in the Methods section (all sources and catalogue numbers are provided). Research material are available upon request. All mass spectrometry data were deposited to ProteomeXchange, https://www.proteomexchange.org/, and jPOST, https://repository.jpostdb.org/, sites with accession numbers PXD045744 and JPST002335, respectively. Source data are provided with this paper.

Research involving human participants, their data, or biological material

Policy information about studies with human participants or human data. See also policy information about sex, gender (identity/presentation), and sexual orientation and race, ethnicity and racism.

Reporting on sex and gender	De-identified formalin-fixed representative sections of the archival blocks from hearts with end stage heart failure were obtained from Dr. Katz's laboratory under protocol # s20-01151 approved by Institutional Review Board at New York University Grossman Medical Center. Sections from archival tissue blocks from patients' post-transplant biopsies with no evidence of rejection and normal ejection fraction was used as normal controls
Reporting on race, ethnicity, or other socially relevant groupings	De-identified formalin-fixed representative sections of the archival blocks from hearts with end stage heart failure were obtained from Dr. Katz's laboratory under protocol # s20-01151 approved by Institutional Review Board at New York University Grossman Medical Center. Sections from archival tissue blocks from patients' post-transplant biopsies with no evidence of rejection and normal ejection fraction was used as normal controls.
Population characteristics	De-identified formalin-fixed representative sections of the archival blocks from hearts with end stage heart failure were obtained from Dr. Katz's laboratory under protocol # s20-01151 approved by Institutional Review Board at New York University Grossman Medical Center. Sections from archival tissue blocks from patients' post-transplant biopsies with no evidence of rejection and normal ejection fraction was used as normal controls.
Recruitment	De-identified formalin-fixed representative sections of the archival blocks from hearts with end stage heart failure were obtained from Dr. Katz's laboratory under protocol # s20-01151 approved by Institutional Review Board at New York University Grossman Medical Center. Sections from archival tissue blocks from patients' post-transplant biopsies with no evidence of rejection and normal ejection fraction was used as normal controls.
Ethics oversight	De-identified formalin-fixed representative sections of the archival blocks from hearts with end stage heart failure were obtained from Dr. Katz's laboratory under protocol # s20-01151 approved by Institutional Review Board at New York University Grossman Medical Center. Sections from archival tissue blocks from patients' post-transplant biopsies with no evidence of rejection and normal ejection fraction was used as normal controls.

Note that full information on the approval of the study protocol must also be provided in the manuscript.

Field-specific reporting

Please select the one below that is the best fit for your research. If you are not sure, read the appropriate sections before making your selection.

☒ Life sciences ☐ Behavioural & social sciences ☐ Ecological, evolutionary & environmental sciences

For a reference copy of the document with all sections, see nature.com/documents/nr-reporting-summary-flat.pdf

Life sciences study design

All studies must disclose on these points even when the disclosure is negative.

Sample size	sample size for animals was chosen to ensure adequate statistical power calculations. samples were chosen from different batches to ensure reproducibility. For in vitro studies, am minimum of 4 biologically independent samples were used arising from a minimum of two independent batches performed from either different source and time points to ensure reproducibility.
Data exclusions	no data was excluded from this manuscript unless stated in the methods.
Replication	All n except otherwise mentioned in figure legends represents biological replicates obtained from minimum of two consecutive batches performed at different time points using different parental source. Each batch comprises of no more than two biological replicates.
Randomization	Mice were randomly assigned to each group. Littermates from the same 10 breeding pairs per genotype were selected for use in this study.
Blinding	For animal experiments, the experimenter was naive to the experimental code or mouse tag number during quantification only. Analysis and extraction for RNAseq and NanoString data was performed by a third person without experimental knowledge. Mt-SR distance and Mito Velocity measurements was performed by experimenters who were blinded by groups. Blinding of all other data was not possible as knowledge of group identity is required for the loading procedure and analysis. Blinding is not necessary since the methods used are objective.

Reporting for specific materials, systems and methods

We require information from authors about some types of materials, experimental systems and methods used in many studies. Here, indicate whether each material, system or method listed is relevant to your study. If you are not sure if a list item applies to your research, read the appropriate section before selecting a response.

Materials & experimental systems

n/a	Involved in the study	
☐	☒	Antibodies
☐	☒	Eukaryotic cell lines
☒	☐	Palaeontology and archaeology
☐	☒	Animals and other organisms
☒	☐	Clinical data
☒	☐	Dual use research of concern
☒	☐	Plants

Methods

n/a	Involved in the study	
☒	☐	ChIP-seq
☐	☒	Flow cytometry
☒	☐	MRI-based neuroimaging

Antibodies

Antibodies used

Antibodies for PLA Host Company Cat # Dilution
DIAPH1 Rabbit ABCAM ab129167 1:200
DIAPH2 Rabbit ABCAM ab181165 1:200
MFN2 Mouse ABCAM ab56889 1:150
MFN1 Mouse SIGMA WH0055669M4 1:200
VDAC1 Mouse ABCAM ab186321 1:200
GRP75 Mouse ABCAM ab2799 1:200
IP3R Mouse ABCAM ab252536 1:200
IP3R1 Rabbit Cell Signaling 8568 1:200
RHOA Mouse ABCAM ab54835 1:200
Antibodies for WB Host Company Cat #
DIAPH1 Rabbit ABCAM ab129167 1:1000
MFN2 Mouse ABCAM ab56889 1:1000
NRF2 Rabbit ABCAM ab137550 1:1000
PINK1 Rabbit Cell Signaling 6946S 1:1000
PARKIN Mouse Cell Signaling 4211S 1:1000
GADD34 Mouse ABCAM ab236516 1:1000
α-TUBULIN Mouse SIGMA T5168 1:25000
TOMM40 Rabbit ABCAM ab185543 1:1000
BCL2 Rabbit ABCAM ab32124 1:1000
NEFL Rabbit Novus Biologicals NB300-131 1:1000
PERK Mouse Cell Signaling 3192S 1:1000
EDEM1 Rabbit ABCAM ab200645 1:1000
DRP1 Rabbit ABCAM ab184247 1:1000

Secondary Antibodies:
Goat polyclonal IRDye 680RD anti-mouse (925-68070, C90910-20, Li-Cor) (1:5000 for WB) Goat polyclonal IRDye 800RD anti-rabbit (925-32211, C90910-II, Li-Cor) (1:5000 for WB)

	For Flow Cytometry:<br>anti-rabbit TNNT2-FITC (ABCAM cat # ab105439)<br>anti-rabbit TNN13 (US Biological, cat # 043107)
Validation	All the primary antibodies used in this study are commercially available and were used for the applications validated by the manufacturers. All IHC experiments included negative controls by omission of primary antibody. All flow cytometry experiments included negative and positive controls, single staining and fluorescence minus one (FMO) controls. Validation and reactivity procedures are described on the websites of the manufacturers

Eukaryotic cell lines

Policy information about cell lines and Sex and Gender in Research

Cell line source(s)	Two different source of Human Induced Pluri-potent Stem cell (HiPSC) were used:<br>Source 1 NCRM1 was obtained from NIH regenerative medicine program.<br>Source 2 Obtained by reprogramming Human adult dermal fibroblasts (cat# 10HU-014) and fibroblast growth medium (cat # MD-0011) were purchased from iXCells Biotechnologies. Pluripotency was validated as shown in Supplement figure la<br>Rat myoblast cell lines, H9C2 cells were obtained from ATCC (cat# CRL-1446).<br>Human microvascular endothelial cells of cardiac origin (HMVEC-C) were obtained from Lonza (cat# CC-7030)<br>HEK-293T cells were obtained from ATCC (cat# CRL-3216).
Authentication	Cells were authenticated as per the supplier's product sheet, safety data sheet, and certificate of analysis.
Mycoplasma contamination	Cells were authenticated as per the supplier's product sheet for negative mycoplasma contamination. NCRM1 was not tested for contamination. we did not observe any abnormal morphology or behavior from any of our cells used.
Commonly misidentified lines<br>(See ICLAC register)	No commonly misidentified cells were used.

Animals and other research organisms

Policy information about studies involving animals; ARRIVE guidelines recommended for reporting animal research, and Sex and Gender in Research

Laboratory animals	All animals used in this study were male mice. Mice (C57BL/6J background) were deficient for global or cardiomyocyte specific Diaphanous 1 (DIAPH1) (Diaphl—/—). For MitoTimer mice, alpha-MHC-MitoTimer mice were crossed with global Diaphl knockout mice to obtain MitoTimer expression in the hearts. All animals were fed with normal chow diet. Experiments were performed between 3-5 months of age.
Wild animals	No wild animals were used in this study.
Reporting on sex	All animals used in the study were males, and used for objective and validation purpose only. Our manuscript refers to validation of proof of concept study and does not intend to study sex and gender differences.
Field-collected samples	No field collected samples were used in the study.
Ethics oversight	All experiments were performed under protocols approved by the New York University Grossman School of Medicine Institute Animal Care Committee (IACUC) in accordance with international and NIH guidelines.

Note that full information on the approval of the study protocol must also be provided in the manuscript.

Flow Cytometry

Plots

Confirm that:

- [x] The axis labels state the marker and fluorochrome used (e.g. CD4-FITC).
- [x] The axis scales are clearly visible. Include numbers along axes only for bottom left plot of group (a 'group' is an analysis of identical markers).
- [x] All plots are contour plots with outliers or pseudocolor plots.
- [x] A numerical value for number of cells or percentage (with statistics) is provided.

Methodology

Sample preparation	shScr and shDIAPH1 HiPSC-CMs were trypsinized, passed through 100 micron filter and re-suspended in cell sorting buffer (50mM Tris pH 7.5, 0.5 mM EDTA and 1% BSA). Cells were incubated with Fc Block (Biolegend) for 30 min at 4°C before staining with fluorescence labeled primary antibodies and washed with sorting buffer prior to analysis. The antibodies used were anti-rabbit TNNT2-FITC (ABCAM cat # ab105439) and anti-rabbit TNNI3 (US Biological, cat # 043107). for Mitochondrial Permeability Transition Pore (MPTP) in HiPSC-CMs exposed to H/R stress was assessed using Mitochondrial Permeability Transition Pore Assay Kit (cat# ab239704, ABCAM). Using a flow-cytometry based approach, the experiment was performed

	as per manufacturer's guidelines. Cells were analyzed using LSR II analyzer (BD Bioscience) available at NYU core facility, processed by FACS DIVA and analyzed using Flowio software.
Instrument	LSR II analyzer (BD Bioscience)
Software	FACSDiva (BD Biosciences) was used to collect the data, and Flowio 10.8.1 (BD Biosciences) was used to analyzed the flow cytometry data.
Cell population abundance	After removing debris, dead cells and aggregates, single cells were —65% of total HiPSC-CMs. Double positive for anti-rabbit TNNT2-FITC (ABCAM cat # ab105439) and anti-rabbit TNNI3-PE (US Biological, cat # 043107) for HiPSC-CM population were greater than 95%.
Gating strategy	Live dead and Singlets were isolated using(FSC-A/SSC-A and FSC-A/FSC-H) from total HiPSC-CMs. Double positive TNNT2-FITC and TNNI3-PE HiPSC-CM population were gated from singlets.

☒ Tick this box to confirm that a figure exemplifying the gating strategy is provided in the Supplementary Information.
