# Supplementary material for: DIAPH1-MFN2 interaction regulates mitochondria-SR/ER contact and modulates ischemic/hypoxic stress
Source: Nat Commun. 2023 Oct 30;14:6900. doi: 10.1038/s41467-023-42521-x (PMC10616211; doi:10.1038/s41467-023-42521-x)
Supplement: Supplementary file 6 — Source data [file 41467_2023_42521_MOESM6_ESM.zip › Source Data/Statistics file_9.29.2023.docx]

## Statistics file

Boyan Zhou and Huilin Li

## Figure 1

Statistical test for fig1_a EM data iPSC-CMs

| Testing.Category | Group.tested | Method | p | Significant | p. | Sample.size |
| --- | --- | --- | --- | --- | --- | --- |
| Group comparison | Group test | Kruskal-Wallis test | 3.60948112446564e-32 | yes | **** | 150 vs 150 vs 150 vs 150 |
| Pairwise comparison | shScr vs shDIAPH1 | Dunn’s Test | 2.11573665940713e-11 | yes | **** | 150 vs 150 |
| Pairwise comparison | shScr vs shScr HR | Dunn’s Test | 0.00193467993855384 | yes | ** | 150 vs 150 |
| Pairwise comparison | shScr vs shDIAPH1 H/R | Dunn’s Test | 2.11573665940713e-11 | yes | **** | 150 vs 150 |
| Pairwise comparison | shDIAPH1 vs shScr HR | Dunn’s Test | 1.12420026354929e-22 | yes | **** | 150 vs 150 |
| Pairwise comparison | shDIAPH1 vs shDIAPH1 H/R | Dunn’s Test | 0.975148299817434 | no | ns | 150 vs 150 |
| Pairwise comparison | shScr HR vs shDIAPH1 H/R | Dunn’s Test | 1.12420026354929e-22 | yes | **** | 150 vs 150 |

Normality test for fig1_a EM data iPSC-CMs

| Group | Method | p | Significant | p. | Whether.pass | Sample.size | SEM |
| --- | --- | --- | --- | --- | --- | --- | --- |
| shScr | Shapiro-Wilk test | 2.89227379123001e-07 | yes | **** | no | 150 | 22.04 +/- 0.9504 |
| shDIAPH1 | Shapiro-Wilk test | 0.000190080399647166 | yes | *** | no | 150 | 33.42 +/- 1.1757 |
| shScr HR | Shapiro-Wilk test | 7.2345328221514e-10 | yes | **** | no | 150 | 17.7667 +/- 0.8738 |
| shDIAPH1 H/R | Shapiro-Wilk test | 0.0533537984333701 | no | ns | yes | 150 | 33.22 +/- 1.1673 |

Statistical test for fig1_b EM data Mice

| Testing.Category | Group.tested | Method | p | Significant | p. | Sample.size |
| --- | --- | --- | --- | --- | --- | --- |
| Group comparison | Group test | Kruskal-Wallis test | 3.24016531528181e-46 | yes | **** | 153 vs 153 vs 155 vs 153 |
| Pairwise comparison | WT - BL vs DKO - BL | Dunn’s Test | 2.47211092896373e-13 | yes | **** | 153 vs 153 |
| Pairwise comparison | WT - BL vs WT - I/R | Dunn’s Test | 0.00010863427201326 | yes | *** | 153 vs 155 |
| Pairwise comparison | WT - BL vs DKO - I/R | Dunn’s Test | 2.44882985126497e-17 | yes | **** | 153 vs 153 |
| Pairwise comparison | DKO - BL vs WT - I/R | Dunn’s Test | 3.38690891582382e-29 | yes | **** | 153 vs 155 |
| Pairwise comparison | DKO - BL vs DKO - I/R | Dunn’s Test | 0.239601839570116 | no | ns | 153 vs 153 |
| Pairwise comparison | WT - I/R vs DKO - I/R | Dunn’s Test | 4.89164387974694e-35 | yes | **** | 155 vs 153 |

Normality test for fig1_b EM data Mice

| Group | Method | p | Significant | p. | Whether.pass | Sample.size | SEM |
| --- | --- | --- | --- | --- | --- | --- | --- |
| WT - BL | Shapiro-Wilk test | 1.09357847536988e-11 | yes | **** | no | 153 | 16.486 +/- 0.7087 |
| DKO - BL | Shapiro-Wilk test | 3.77824256933662e-07 | yes | **** | no | 153 | 24.8405 +/- 0.8549 |
| WT - I/R | Shapiro-Wilk test | 1.5084132203688e-13 | yes | **** | no | 155 | 12.8542 +/- 0.6068 |
| DKO - I/R | Shapiro-Wilk test | 4.5423054157861e-10 | yes | **** | no | 153 | 28.0456 +/- 1.1235 |

Statistical test for fig1_e DUOLINK IPSCCMs

| Testing.Category | Group.tested | Method | p | Significant | p. | Sample.size |
| --- | --- | --- | --- | --- | --- | --- |
| Group comparison | Group test | ANOVA | 8.47750903460055e-06 | yes | **** | 4 vs 4 vs 4 vs 4 |
| Pairwise comparison | shScr vs shDIAPH1 | TukeyHSD | 0.00200636308286473 | yes | ** | 4 vs 4 |
| Pairwise comparison | shScr vs shScr H/R | TukeyHSD | 0.0287010611258354 | yes | * | 4 vs 4 |
| Pairwise comparison | shScr vs shDIAPH1 H/R | TukeyHSD | 0.00538024827668615 | yes | ** | 4 vs 4 |
| Pairwise comparison | shDIAPH1 vs shScr H/R | TukeyHSD | 1.67450660160107e-05 | yes | **** | 4 vs 4 |
| Pairwise comparison | shDIAPH1 vs shDIAPH1 H/R | TukeyHSD | 0.935175955351035 | no | ns | 4 vs 4 |
| Pairwise comparison | shScr H/R vs shDIAPH1 H/R | TukeyHSD | 3.55516873817852e-05 | yes | **** | 4 vs 4 |

Normality test for fig1_e DUOLINK IPSCCMs

| Group | Method | p | Significant | p. | Whether.pass | Sample.size | SEM |
| --- | --- | --- | --- | --- | --- | --- | --- |
| shScr | Shapiro-Wilk test | 0.397408028240305 | no | ns | yes | 4 | 65.4443 +/- 10.3498 |
| shDIAPH1 | Shapiro-Wilk test | 0.735701185757224 | no | ns | yes | 4 | 11.0662 +/- 3.0315 |
| shScr H/R | Shapiro-Wilk test | 0.565685484802689 | no | ns | yes | 4 | 102.449 +/- 11.0972 |
| shDIAPH1 H/R | Shapiro-Wilk test | 0.562969175978098 | no | ns | yes | 4 | 17.6326 +/- 3.7252 |

Statistical test for figure 1f

| Testing.Category | Group.tested | Method | p | Significant | p. | Sample.size |
| --- | --- | --- | --- | --- | --- | --- |
| Group test | Welch's ANOVA | 6.26499861439329e-06 | yes | **** | 6 vs 6 vs 7 vs 7 vs 9 | Group test |
| TS-20-12480(C) vs TS-20-16337(C) | t-test with pooled SD | 0.413676262608233 | no | ns | 6 vs 6 | TS-20-12480(C) vs TS-20-16337(C) |
| TS-20-12480(C) vs TS-18-21027(Isch-CM) | t-test with pooled SD | 3.15509186269924e-06 | yes | **** | 6 vs 7 | TS-20-12480(C) vs TS-18-21027(Isch-CM) |
| TS-20-12480(C) vs TS-18-27101(Isch-CM) | t-test with pooled SD | 3.67793501641919e-07 | yes | **** | 6 vs 7 | TS-20-12480(C) vs TS-18-27101(Isch-CM) |
| TS-20-12480(C) vs TS-18-27101(Isch-CM) NR | t-test with pooled SD | 0.400322704745469 | no | ns | 6 vs 9 | TS-20-12480(C) vs TS-18-27101(Isch-CM) NR |
| TS-20-16337(C) vs TS-18-21027(Isch-CM) | t-test with pooled SD | 2.96415414514978e-05 | yes | **** | 6 vs 7 | TS-20-16337(C) vs TS-18-21027(Isch-CM) |
| TS-20-16337(C) vs TS-18-27101(Isch-CM) | t-test with pooled SD | 2.95399926052303e-06 | yes | **** | 6 vs 7 | TS-20-16337(C) vs TS-18-27101(Isch-CM) |
| TS-20-16337(C) vs TS-18-27101(Isch-CM) NR | t-test with pooled SD | 0.108742276849834 | no | ns | 6 vs 9 | TS-20-16337(C) vs TS-18-27101(Isch-CM) NR |
| TS-18-21027(Isch-CM) vs TS-18-27101(Isch-CM) | t-test with pooled SD | 0.400322704745469 | no | ns | 7 vs 7 | TS-18-21027(Isch-CM) vs TS-18-27101(Isch-CM) |
| TS-18-21027(Isch-CM) vs TS-18-27101(Isch-CM) NR | t-test with pooled SD | 1.0264300845506e-07 | yes | **** | 7 vs 9 | TS-18-21027(Isch-CM) vs TS-18-27101(Isch-CM) NR |
| TS-18-27101(Isch-CM) vs TS-18-27101(Isch-CM) NR | t-test with pooled SD | 1.32036204922401e-08 | yes | **** | 7 vs 9 | TS-18-27101(Isch-CM) vs TS-18-27101(Isch-CM) NR |

Normality test for figure1h

| Group | Method | p | Significant | p. | Whether.pass | Sample.size | SEM |
| --- | --- | --- | --- | --- | --- | --- | --- |
| TS-20-12480(C) | Shapiro-Wilk test | 0.119781422757382 | no | ns | yes | 6 | 2.2965 +/- 0.2999 |
| TS-20-16337(C) | Shapiro-Wilk test | 0.698301858161908 | no | ns | yes | 6 | 3.103 +/- 0.1239 |
| TS-18-21027(Isch-CM) | Shapiro-Wilk test | 0.845286007289161 | no | ns | yes | 7 | 7.8801 +/- 0.7489 |
| TS-18-27101(Isch-CM) | Shapiro-Wilk test | 0.253697684828709 | no | ns | yes | 7 | 8.7835 +/- 1.1227 |
| TS-18-27101(Isch-CM) NR | Shapiro-Wilk test | 0.623818919001384 | no | ns | yes | 9 | 1.4714 +/- 0.2771 |

Analysis for Fig 1f

**Method:**

To compare the mean number of interaction/nucleus between control (ctrl) patients and patients with ischemic heart disease, we fit the following linear mixed model (LMM):

Number ~ Disease_status + (1|Patient_ID).

To test the difference between Isch-CM and Isch-CM_BZ, we first test the normality of data by the Shapiro-Wilk test. Since they both pass the normality test, we conduct the unpaired *t*-test.

**Result:**

1. LMM: comparison between disease patients and control patients

Estimate Std. Error df t value Pr(>|t|)

(Intercept) 2.699765 0.5415479 24 4.985274 4.314934e-05

TreatmentIsch-CM 5.632021 0.7380054 24 7.631409 7.198365e-08

The patients with ischemic heart disease have about 5.632 more interactions/nucleus than control patients (p=7.198365e-08).

1. Two samples test:

| Groups | Statistic Test | p | Significant | p* | Sample Size |
| --- | --- | --- | --- | --- | --- |
| Isch-CM vs Isch-CM_BZ | unpaired *t*-test | 0.000462 | yes | *** | 7 vs 9 |

| Group | Normality Test | p | Significant | p* | Pass normality test | Sample Size | SEM |
| --- | --- | --- | --- | --- | --- | --- | --- |
| Isch-CM | Shapiro-Wilk test | 0.253698 | no | ns | yes | 7 | 8.7835 +/- 1.1227 |
| Isch-CM_BZ | Shapiro-Wilk test | 0.623819 | no | ns | yes | 9 | 1.4714 +/- 0.2771 |

## Supplementary Figure 1 supporting figure 1

Statistical test for fig 1d

| Groups | Statistic.Test | p | Significant | p. | Sample.Size |
| --- | --- | --- | --- | --- | --- |
| shScr vs shDiaph1 | wilcoxon rank-sum test | 2.41302517784007e-17 | yes | **** | 150 vs 150 |

Normality test for fig 1d

| Group | Normality.Test | p | Significant | p. | Pass.normality.test. | Sample.Size | SEM |
| --- | --- | --- | --- | --- | --- | --- | --- |
| shScr | Shapiro-Wilk test | 4.6416464313923e-10 | yes | **** | no | 150 | 20.1146 +/- 0.8468 |
| shDiaph1 | Shapiro-Wilk test | 0.000260638145363851 | yes | *** | no | 150 | 32.5867 +/- 1.1105 |

Statistical test for fig 1g H9C2

| Groups | Statistic.Test | p | Significant | p. | Sample.Size |
| --- | --- | --- | --- | --- | --- |
| shScr vs shDiaph1 | unpaired t-test | 4.85167845011576e-05 | yes | **** | 4 vs 4 |

Normality test for fig 1g H9C2

| Group | Normality.Test | p | Significant | p. | Pass.normality.test. | Sample.Size | SEM |
| --- | --- | --- | --- | --- | --- | --- | --- |
| shScr | Shapiro-Wilk test | 0.660738062673617 | no | ns | yes | 4 | 1.0077 +/- 0.0723 |
| shDiaph1 | Shapiro-Wilk test | 0.304801545571819 | no | ns | yes | 4 | 0.221 +/- 0.0244 |

Statistical test for fig 1g HMVECs

| Groups | Statistic.Test | p | Significant | p. | Sample.Size |
| --- | --- | --- | --- | --- | --- |
| shScr vs shDIAPH1 | unpaired t-test | 0.000323093980249212 | yes | *** | 4 vs 4 |

Normality test for fig 1g HMVECs

| Group | Normality.Test | p | Significant | p. | Pass.normality.test. | Sample.Size | SEM |
| --- | --- | --- | --- | --- | --- | --- | --- |
| shScr | Shapiro-Wilk test | 0.959646809363284 | no | ns | yes | 4 | 1.0102 +/- 0.0827 |
| shDIAPH1 | Shapiro-Wilk test | 0.269842517156623 | no | ns | yes | 4 | 0.2575 +/- 0.0602 |

Statistical test for fig 1g BMDMs

| Groups | Statistic.Test | p | Significant | p. | Sample.Size |
| --- | --- | --- | --- | --- | --- |
| Wt vs gDKO | wilcoxon rank-sum test | 0.0285714285714286 | yes | * | 4 vs 4 |

Normality test for fig 1g BMDMs

| Group | Normality.Test | p | Significant | p. | Pass.normality.test. | Sample.Size | SEM |
| --- | --- | --- | --- | --- | --- | --- | --- |
| Wt | Shapiro-Wilk test | 0.0437572255792858 | yes | * | no | 4 | 1.0428 +/- 0.1707 |
| gDKO | Shapiro-Wilk test | 0.0434799490656101 | yes | * | no | 4 | 0.1059 +/- 0.0259 |

Statistical test for fig 1h

| Groups | Statistic.Test | p | Significant | p. | Sample.Size |
| --- | --- | --- | --- | --- | --- |
| shScr vs shDiaph1 | unpaired t-test | 0.0238039193076293 | yes | * | 4 vs 4 |

Normality test for fig 1h

| Group | Normality.Test | p | Significant | p. | Pass.normality.test. | Sample.Size | SEM |
| --- | --- | --- | --- | --- | --- | --- | --- |
| shScr | Shapiro-Wilk test | 0.647794336766089 | no | ns | yes | 4 | 11.375 +/- 2.6173 |
| shDiaph1 | Shapiro-Wilk test | 0.577354901880098 | no | ns | yes | 4 | 0.24 +/- 0.0108 |

Statistical test for fig 1i

| Groups | Statistic.Test | p | Significant | p. | Sample.Size |
| --- | --- | --- | --- | --- | --- |
| shScr vs shDIAPH1 | unpaired t-test | 0.0472059956564903 | yes | * | 4 vs 4 |

Normality test for fig 1i

| Group | Normality.Test | p | Significant | p. | Pass.normality.test. | Sample.Size | SEM |
| --- | --- | --- | --- | --- | --- | --- | --- |
| shScr | Shapiro-Wilk test | 0.585885625759469 | no | ns | yes | 4 | 52.4785 +/- 14.286 |
| shDIAPH1 | Shapiro-Wilk test | 0.323025991126571 | no | ns | yes | 4 | 6.6715 +/- 2.3302 |

Statistical test for fig 1j

| Groups | Statistic.Test | p | Significant | p. | Sample.Size |
| --- | --- | --- | --- | --- | --- |
| Wt vs gDKO | wilcoxon rank-sum test | 0.0285714285714286 | yes | * | 4 vs 4 |

Normality test for fig 1j

| Group | Normality.Test | p | Significant | p. | Pass.normality.test. | Sample.Size | SEM |
| --- | --- | --- | --- | --- | --- | --- | --- |
| Wt | Shapiro-Wilk test | 0.473747714646523 | no | ns | yes | 4 | 22.3155 +/- 3.3806 |
| gDKO | Shapiro-Wilk test | 0.0165782265148423 | yes | * | no | 4 | 7.3657 +/- 1.6798 |

Statistical test for fig 1k F/G actin

| Testing.Category | Group.tested | Method | p | Significant | p. | Sample.size |
| --- | --- | --- | --- | --- | --- | --- |
| Group comparison | Group test | ANOVA | 2.93582123674315e-06 | yes | **** | 6 vs 6 vs 6 vs 6 |
| Pairwise comparison | shScr vs shDIAPH1 | TukeyHSD | 3.06622017099967e-05 | yes | **** | 6 vs 6 |
| Pairwise comparison | shScr vs shScr H/R | TukeyHSD | 0.754934105246052 | no | ns | 6 vs 6 |
| Pairwise comparison | shScr vs shDIAPH1 H/R | TukeyHSD | 0.0841357704105087 | no | ns | 6 vs 6 |
| Pairwise comparison | shDIAPH1 vs shScr H/R | TukeyHSD | 3.80320910753351e-06 | yes | **** | 6 vs 6 |
| Pairwise comparison | shDIAPH1 vs shDIAPH1 H/R | TukeyHSD | 0.00920000724652548 | yes | ** | 6 vs 6 |
| Pairwise comparison | shScr H/R vs shDIAPH1 H/R | TukeyHSD | 0.0103929348734079 | yes | * | 6 vs 6 |

Normality test for fig 1k F/G actin

| Group | Method | p | Significant | p. | Whether.pass | Sample.size | SEM |
| --- | --- | --- | --- | --- | --- | --- | --- |
| shScr | Shapiro-Wilk test | 0.491186715996283 | no | ns | yes | 6 | 0.9483 +/- 0.0382 |
| shDIAPH1 | Shapiro-Wilk test | 0.895679289055773 | no | ns | yes | 6 | 0.6176 +/- 0.0501 |
| shScr H/R | Shapiro-Wilk test | 0.886151111158952 | no | ns | yes | 6 | 1.002 +/- 0.0355 |
| shDIAPH1 H/R | Shapiro-Wilk test | 0.787455482756938 | no | ns | yes | 6 | 0.8112 +/- 0.0247 |

Statistical test for fig 1l DUOLINK LATB

| Testing.Category | Group.tested | Method | p | Significant | p. | Sample.size |
| --- | --- | --- | --- | --- | --- | --- |
| Group comparison | Group test | Welch’s ANOVA | 0.00160786922097665 | yes | ** | 4 vs 4 vs 4 vs 4 |
| Pairwise comparison | Veh BL vs LATB BL | t-test with pooled SD | 0.00734390217078443 | yes | ** | 4 vs 4 |
| Pairwise comparison | Veh BL vs Veh H/R | t-test with pooled SD | 0.000287432939649408 | yes | *** | 4 vs 4 |
| Pairwise comparison | Veh BL vs LATB H/R | t-test with pooled SD | 0.00734390217078443 | yes | ** | 4 vs 4 |
| Pairwise comparison | LATB BL vs Veh H/R | t-test with pooled SD | 4.26769469774986e-06 | yes | **** | 4 vs 4 |
| Pairwise comparison | LATB BL vs LATB H/R | t-test with pooled SD | 0.969564163795187 | no | ns | 4 vs 4 |
| Pairwise comparison | Veh H/R vs LATB H/R | t-test with pooled SD | 4.26769469774986e-06 | yes | **** | 4 vs 4 |

Normality test for fig4_g DUOLINK LATB

| Group | Method | p | Significant | p. | Whether.pass | Sample.size | SEM |
| --- | --- | --- | --- | --- | --- | --- | --- |
| Veh BL | Shapiro-Wilk test | 0.248868465117968 | no | ns | yes | 4 | 17.6504 +/- 3.4722 |
| LATB BL | Shapiro-Wilk test | 0.216718916047286 | no | ns | yes | 4 | 1.138 +/- 0.1025 |
| Veh H/R | Shapiro-Wilk test | 0.375968350053896 | no | ns | yes | 4 | 44.8474 +/- 6.1178 |
| LATB H/R | Shapiro-Wilk test | 0.0602307995662259 | no | ns | yes | 4 | 0.9442 +/- 0.068 |

## Supplementary Figure 3 supporting figure 1

Statistical test for S3e_VDAC-IP3R DUOLINK

| Groups | Statistic.Test | p | Significant | p. | Sample.Size |
| --- | --- | --- | --- | --- | --- |
| shScr H/R vs shDIAPH1 H/R | unpaired t-test | 0.0307252268665215 | yes | * | 4 vs 4 |

Normality test for Rev_Figure_VDAC

| Group | Normality.Test | p | Significant | p. | Pass.normality.test. | Sample.Size | SEM |
| --- | --- | --- | --- | --- | --- | --- | --- |
| shScr H/R | Shapiro-Wilk test | 0.0507739063655237 | no | ns | yes | 4 | 1.1421 +/- 0.2094 |
| shDIAPH1 H/R | Shapiro-Wilk test | 0.0991207004274071 | no | ns | yes | 4 | 0.3363 +/- 0.0124 |

## Figure 3

*Statistical test for figure3a*

| Fig 3a LDH iPSC-CMs H/R | shScr H/R vs shDIAPH1 H/R | unpaired t-test | 0.00187815433088463 | yes | ** | 4 vs 4 |
| --- | --- | --- | --- | --- | --- | --- |

## *Normality test for figure1*

| Fig 3a LDH iPSC-CMs H/R | shDIAPH1 H/R | Shapiro-Wilk test | 0.132834480692908 | no | ns | yes | 4 | 0.001 +/- 1e-04 |
| --- | --- | --- | --- | --- | --- | --- | --- | --- |

PCR stats 3f

Statistical test for DIAPH1

| Testing.Category | Group.tested | Method | p | Significant | p. | Sample.size |
| --- | --- | --- | --- | --- | --- | --- |
| Group comparison | Group test | Welch’s ANOVA | 0.00058324788307599 | yes | *** | 4 vs 4 vs 4 vs 4 |
| Pairwise comparison | shScr vs shDIAPH1 | Games-Howell Test | 0.012 | yes | * | 4 vs 4 |
| Pairwise comparison | shScr vs shScr H/R | Games-Howell Test | 0.18 | no | ns | 4 vs 4 |
| Pairwise comparison | shScr vs shDIAPH1 H/R | Games-Howell Test | 0.002 | yes | ** | 4 vs 4 |
| Pairwise comparison | shDIAPH1 vs shScr H/R | Games-Howell Test | 0.063 | no | ns | 4 vs 4 |
| Pairwise comparison | shDIAPH1 vs shDIAPH1 H/R | Games-Howell Test | 0.165 | no | ns | 4 vs 4 |
| Pairwise comparison | shScr H/R vs shDIAPH1 H/R | Games-Howell Test | 0.047 | yes | * | 4 vs 4 |

Normality test for DIAPH1

| Group | Method | p | Significant | p. | Whether.pass | Sample.size | SEM |
| --- | --- | --- | --- | --- | --- | --- | --- |
| shScr | Shapiro-Wilk test | 0.779035933432642 | no | ns | yes | 4 | 1.0095 +/- 0.0788 |
| shDIAPH1 | Shapiro-Wilk test | 0.12738438056277 | no | ns | yes | 4 | 0.4453 +/- 0.0861 |
| shScr H/R | Shapiro-Wilk test | 0.823322783311458 | no | ns | yes | 4 | 2.1132 +/- 0.39 |
| shDIAPH1 H/R | Shapiro-Wilk test | 0.995596899462747 | no | ns | yes | 4 | 0.1942 +/- 0.0335 |

Statistical test for TOMM40

| Testing.Category | Group.tested | Method | p | Significant | p. | Sample.size |
| --- | --- | --- | --- | --- | --- | --- |
| Group comparison | Group test | Kruskal-Wallis test | 0.0149112838974369 | yes | * | 4 vs 4 vs 4 vs 4 |
| Pairwise comparison | shScr vs shDIAPH1 | Dunn’s Test | 0.281716395393203 | no | ns | 4 vs 4 |
| Pairwise comparison | shScr vs shScr H/R | Dunn’s Test | 0.94080265650735 | no | ns | 4 vs 4 |
| Pairwise comparison | shScr vs shDIAPH1 H/R | Dunn’s Test | 0.0180066957872326 | yes | * | 4 vs 4 |
| Pairwise comparison | shDIAPH1 vs shScr H/R | Dunn’s Test | 0.281716395393203 | no | ns | 4 vs 4 |
| Pairwise comparison | shDIAPH1 vs shDIAPH1 H/R | Dunn’s Test | 0.237764503394827 | no | ns | 4 vs 4 |
| Pairwise comparison | shScr H/R vs shDIAPH1 H/R | Dunn’s Test | 0.0180066957872326 | yes | * | 4 vs 4 |

Normality test for TOMM40

| Group | Method | p | Significant | p. | Whether.pass | Sample.size | SEM |
| --- | --- | --- | --- | --- | --- | --- | --- |
| shScr | Shapiro-Wilk test | 0.0369065425322671 | yes | * | no | 4 | 1.0218 +/- 0.1305 |
| shDIAPH1 | Shapiro-Wilk test | 0.281036588672833 | no | ns | yes | 4 | 0.6001 +/- 0.1442 |
| shScr H/R | Shapiro-Wilk test | 0.506720690386913 | no | ns | yes | 4 | 1.039 +/- 0.167 |
| shDIAPH1 H/R | Shapiro-Wilk test | 0.909672994928887 | no | ns | yes | 4 | 0.1373 +/- 0.0436 |

Statistical test for NRF2

| Testing.Category | Group.tested | Method | p | Significant | p. | Sample.size |
| --- | --- | --- | --- | --- | --- | --- |
| Group comparison | Group test | ANOVA | 2.53648022663836e-07 | yes | **** | 4 vs 4 vs 4 vs 4 |
| Pairwise comparison | shScr vs shDIAPH1 | TukeyHSD | 0.00117406195402769 | yes | ** | 4 vs 4 |
| Pairwise comparison | shScr vs shScr H/R | TukeyHSD | 0.115977332476558 | no | ns | 4 vs 4 |
| Pairwise comparison | shScr vs shDIAPH1 H/R | TukeyHSD | 3.89445833626212e-06 | yes | **** | 4 vs 4 |
| Pairwise comparison | shDIAPH1 vs shScr H/R | TukeyHSD | 3.14814701983801e-05 | yes | **** | 4 vs 4 |
| Pairwise comparison | shDIAPH1 vs shDIAPH1 H/R | TukeyHSD | 0.00602734314978648 | yes | ** | 4 vs 4 |
| Pairwise comparison | shScr H/R vs shDIAPH1 H/R | TukeyHSD | 3.04707618248301e-07 | yes | **** | 4 vs 4 |

Normality test for NRF2

| Group | Method | p | Significant | p. | Whether.pass | Sample.size | SEM |
| --- | --- | --- | --- | --- | --- | --- | --- |
| shScr | Shapiro-Wilk test | 0.817204354135727 | no | ns | yes | 4 | 1.0052 +/- 0.0595 |
| shDIAPH1 | Shapiro-Wilk test | 0.395123247793072 | no | ns | yes | 4 | 1.6119 +/- 0.1072 |
| shScr H/R | Shapiro-Wilk test | 0.11364592834599 | no | ns | yes | 4 | 0.7146 +/- 0.0798 |
| shDIAPH1 H/R | Shapiro-Wilk test | 0.492299641053068 | no | ns | yes | 4 | 2.1039 +/- 0.0792 |

Statistical test for BCL2

| Testing.Category | Group.tested | Method | p | Significant | p. | Sample.size |
| --- | --- | --- | --- | --- | --- | --- |
| Group comparison | Group test | ANOVA | 7.83505840963411e-06 | yes | **** | 4 vs 4 vs 4 vs 4 |
| Pairwise comparison | shScr vs shDIAPH1 | TukeyHSD | 0.000127861159554876 | yes | *** | 4 vs 4 |
| Pairwise comparison | shScr vs shScr H/R | TukeyHSD | 0.961023772432706 | no | ns | 4 vs 4 |
| Pairwise comparison | shScr vs shDIAPH1 H/R | TukeyHSD | 0.000226512890393216 | yes | *** | 4 vs 4 |
| Pairwise comparison | shDIAPH1 vs shScr H/R | TukeyHSD | 6.46677088698144e-05 | yes | **** | 4 vs 4 |
| Pairwise comparison | shDIAPH1 vs shDIAPH1 H/R | TukeyHSD | 0.978721523443889 | no | ns | 4 vs 4 |
| Pairwise comparison | shScr H/R vs shDIAPH1 H/R | TukeyHSD | 0.000111863367799558 | yes | *** | 4 vs 4 |

Normality test for BCL2

| Group | Method | p | Significant | p. | Whether.pass | Sample.size | SEM |
| --- | --- | --- | --- | --- | --- | --- | --- |
| shScr | Shapiro-Wilk test | 0.682297151293465 | no | ns | yes | 4 | 1.0013 +/- 0.0289 |
| shDIAPH1 | Shapiro-Wilk test | 0.566959734131144 | no | ns | yes | 4 | 1.7167 +/- 0.0516 |
| shScr H/R | Shapiro-Wilk test | 0.331752358112941 | no | ns | yes | 4 | 0.9488 +/- 0.0619 |
| shDIAPH1 H/R | Shapiro-Wilk test | 0.229304652950095 | no | ns | yes | 4 | 1.6743 +/- 0.1269 |

Statistical test for NEFL

| Testing.Category | Group.tested | Method | p | Significant | p. | Sample.size |
| --- | --- | --- | --- | --- | --- | --- |
| Group comparison | Group test | ANOVA | 4.67469602247669e-05 | yes | **** | 4 vs 4 vs 4 vs 4 |
| Pairwise comparison | shScr vs shDIAPH1 | TukeyHSD | 0.000102521476758932 | yes | *** | 4 vs 4 |
| Pairwise comparison | shScr vs shScr H/R | TukeyHSD | 0.999998511203212 | no | ns | 4 vs 4 |
| Pairwise comparison | shScr vs shDIAPH1 H/R | TukeyHSD | 0.0292072204106628 | yes | * | 4 vs 4 |
| Pairwise comparison | shDIAPH1 vs shScr H/R | TukeyHSD | 0.000100260359368165 | yes | *** | 4 vs 4 |
| Pairwise comparison | shDIAPH1 vs shDIAPH1 H/R | TukeyHSD | 0.0203228657657551 | yes | * | 4 vs 4 |
| Pairwise comparison | shScr H/R vs shDIAPH1 H/R | TukeyHSD | 0.0284134809341214 | yes | * | 4 vs 4 |

Normality test for NEFL

| Group | Method | p | Significant | p. | Whether.pass | Sample.size | SEM |
| --- | --- | --- | --- | --- | --- | --- | --- |
| shScr | Shapiro-Wilk test | 0.20786948685298 | no | ns | yes | 4 | 1.0433 +/- 0.1851 |
| shDIAPH1 | Shapiro-Wilk test | 0.0515073004669421 | no | ns | yes | 4 | 3.4417 +/- 0.1836 |
| shScr H/R | Shapiro-Wilk test | 0.478891526438469 | no | ns | yes | 4 | 1.0377 +/- 0.1882 |
| shDIAPH1 H/R | Shapiro-Wilk test | 0.454883172735534 | no | ns | yes | 4 | 2.2058 +/- 0.3849 |

Statistical test for PERK

| Testing.Category | Group.tested | Method | p | Significant | p. | Sample.size |
| --- | --- | --- | --- | --- | --- | --- |
| Group comparison | Group test | ANOVA | 2.69916859071474e-09 | yes | **** | 4 vs 4 vs 4 vs 4 |
| Pairwise comparison | shScr vs shDIAPH1 | TukeyHSD | 2.79427165185142e-05 | yes | **** | 4 vs 4 |
| Pairwise comparison | shScr vs shScr H/R | TukeyHSD | 2.86599208016991e-05 | yes | **** | 4 vs 4 |
| Pairwise comparison | shScr vs shDIAPH1 H/R | TukeyHSD | 3.39378823976944e-06 | yes | **** | 4 vs 4 |
| Pairwise comparison | shDIAPH1 vs shScr H/R | TukeyHSD | 1.48923934295553e-08 | yes | **** | 4 vs 4 |
| Pairwise comparison | shDIAPH1 vs shDIAPH1 H/R | TukeyHSD | 0.34536738917952 | no | ns | 4 vs 4 |
| Pairwise comparison | shScr H/R vs shDIAPH1 H/R | TukeyHSD | 4.60324123263689e-09 | yes | **** | 4 vs 4 |

Normality test for PERK

| Group | Method | p | Significant | p. | Whether.pass | Sample.size | SEM |
| --- | --- | --- | --- | --- | --- | --- | --- |
| shScr | Shapiro-Wilk test | 0.166788975488255 | no | ns | yes | 4 | 1.0009 +/- 0.0243 |
| shDIAPH1 | Shapiro-Wilk test | 0.374366026851842 | no | ns | yes | 4 | 0.5445 +/- 0.025 |
| shScr H/R | Shapiro-Wilk test | 0.612249900609269 | no | ns | yes | 4 | 1.4562 +/- 0.0555 |
| shDIAPH1 H/R | Shapiro-Wilk test | 0.21852449826609 | no | ns | yes | 4 | 0.4414 +/- 0.052 |

Statistical test of gaddd34

| Testing Category | Group tested | Method | p | Significant | p* | Sample size |
| --- | --- | --- | --- | --- | --- | --- |
| Group comparison | Group test | Welch’s ANOVA | 0.000673640071604387 | yes | *** | 4 vs 4 vs 4 vs 4 |
| Pairwise comparison | shScr vs shDIAPH1 | Games-Howell Test | 0.109 | no | ns | 4 vs 4 |
| Pairwise comparison | shScr vs HR-shScr | Games-Howell Test | 0.202 | no | ns | 4 vs 4 |
| Pairwise comparison | shScr vs HR-shDIAPH1 | Games-Howell Test | 0.000516 | yes | *** | 4 vs 4 |
| Pairwise comparison | shDIAPH1 vs HR-shScr | Games-Howell Test | 0.066 | no | ns | 4 vs 4 |
| Pairwise comparison | shDIAPH1 vs HR-shDIAPH1 | Games-Howell Test | 0.148 | no | ns | 4 vs 4 |
| Pairwise comparison | HR-shScr vs HR-shDIAPH1 | Games-Howell Test | 0.035 | yes | * | 4 vs 4 |

Normality test of gaddd34

| Group | Method | p | Significant | p* | Whether pass | Sample size | SEM |
| --- | --- | --- | --- | --- | --- | --- | --- |
| shScr | Shapiro-Wilk test | 0.616913126959569 | no | ns | yes | 4 | 1.0059 +/- 0.0614 |
| shDIAPH1 | Shapiro-Wilk test | 0.292266260040894 | no | ns | yes | 4 | 0.6016 +/- 0.1187 |
| HR-shScr | Shapiro-Wilk test | 0.803258849858435 | no | ns | yes | 4 | 1.8067 +/- 0.2982 |
| HR-shDIAPH1 | Shapiro-Wilk test | 0.713939094085854 | no | ns | yes | 4 | 0.2369 +/- 0.0629 |

Statistical test for EDEM1

| Testing.Category | Group.tested | Method | p | Significant | p. | Sample.size |
| --- | --- | --- | --- | --- | --- | --- |
| Group comparison | Group test | ANOVA | 8.4225985870239e-06 | yes | **** | 4 vs 4 vs 4 vs 4 |
| Pairwise comparison | shScr vs shDIAPH1 | TukeyHSD | 0.00180982809623997 | yes | ** | 4 vs 4 |
| Pairwise comparison | shScr vs shScr H/R | TukeyHSD | 0.661639618539679 | no | ns | 4 vs 4 |
| Pairwise comparison | shScr vs shDIAPH1 H/R | TukeyHSD | 9.60450853717543e-05 | yes | **** | 4 vs 4 |
| Pairwise comparison | shDIAPH1 vs shScr H/R | TukeyHSD | 0.000289077480086886 | yes | *** | 4 vs 4 |
| Pairwise comparison | shDIAPH1 vs shDIAPH1 H/R | TukeyHSD | 0.272509500114417 | no | ns | 4 vs 4 |
| Pairwise comparison | shScr H/R vs shDIAPH1 H/R | TukeyHSD | 2.02610778773371e-05 | yes | **** | 4 vs 4 |

Normality test for EDEM1

| Group | Method | p | Significant | p. | Whether.pass | Sample.size | SEM |
| --- | --- | --- | --- | --- | --- | --- | --- |
| shScr | Shapiro-Wilk test | 0.267246600113155 | no | ns | yes | 4 | 1.0054 +/- 0.0602 |
| shDIAPH1 | Shapiro-Wilk test | 0.143688147003532 | no | ns | yes | 4 | 0.4129 +/- 0.0473 |
| shScr H/R | Shapiro-Wilk test | 0.235940378535147 | no | ns | yes | 4 | 1.1459 +/- 0.1479 |
| shDIAPH1 H/R | Shapiro-Wilk test | 0.77702288301493 | no | ns | yes | 4 | 0.181 +/- 0.04 |

Statistical test for PARKIN

| Testing.Category | Group.tested | Method | p | Significant | p. | Sample.size |
| --- | --- | --- | --- | --- | --- | --- |
| Group comparison | Group test | Welch’s ANOVA | 0.00150564151628178 | yes | ** | 4 vs 4 vs 4 vs 4 |
| Pairwise comparison | shScr vs shDIAPH1 | Games-Howell Test | 0.01 | yes | * | 4 vs 4 |
| Pairwise comparison | shScr vs shScr H/R | Games-Howell Test | 0.272 | no | ns | 4 vs 4 |
| Pairwise comparison | shScr vs shDIAPH1<U+00A0>H/R | Games-Howell Test | 0.062 | no | ns | 4 vs 4 |
| Pairwise comparison | shDIAPH1 vs shScr H/R | Games-Howell Test | 0.005 | yes | ** | 4 vs 4 |
| Pairwise comparison | shDIAPH1 vs shDIAPH1<U+00A0>H/R | Games-Howell Test | 0.391 | no | ns | 4 vs 4 |
| Pairwise comparison | shScr H/R vs shDIAPH1<U+00A0>H/R | Games-Howell Test | 0.041 | yes | * | 4 vs 4 |

Normality test for PARKIN

| Group | Method | p | Significant | p. | Whether.pass | Sample.size | SEM |
| --- | --- | --- | --- | --- | --- | --- | --- |
| shScr | Shapiro-Wilk test | 0.932512636564979 | no | ns | yes | 4 | 1.001 +/- 0.026 |
| shDIAPH1 | Shapiro-Wilk test | 0.382369192782067 | no | ns | yes | 4 | 4.0362 +/- 0.3571 |
| shScr H/R | Shapiro-Wilk test | 0.0713664721999879 | no | ns | yes | 4 | 0.7581 +/- 0.1045 |
| shDIAPH1<U+00A0>H/R | Shapiro-Wilk test | 0.233678162510981 | no | ns | yes | 4 | 3.0316 +/- 0.459 |

## Supplement figure 7 supporting Figure 3

Western blotting

Statistical test for DIAPH1

| Testing.Category | Group.tested | Method | p | Significant | p. | Sample.size |
| --- | --- | --- | --- | --- | --- | --- |
| Group comparison | Group test | ANOVA | 5.59597364582101e-07 | yes | **** | 4 vs 4 vs 4 vs 4 |
| Pairwise comparison | shScr vs shDIAPH1 | TukeyHSD | 0.000793924552290659 | yes | *** | 4 vs 4 |
| Pairwise comparison | shScr vs shScr H/R | TukeyHSD | 0.00747358929906305 | yes | ** | 4 vs 4 |
| Pairwise comparison | shScr vs shDIAPH1 H/R | TukeyHSD | 0.000146639464313814 | yes | *** | 4 vs 4 |
| Pairwise comparison | shDIAPH1 vs shScr H/R | TukeyHSD | 3.40430239020861e-06 | yes | **** | 4 vs 4 |
| Pairwise comparison | shDIAPH1 vs shDIAPH1 H/R | TukeyHSD | 0.689718904013297 | no | ns | 4 vs 4 |
| Pairwise comparison | shScr H/R vs shDIAPH1 H/R | TukeyHSD | 1.0309167948197e-06 | yes | **** | 4 vs 4 |

Normality test for DIAPH1

| Group | Method | p | Significant | p. | Whether.pass | Sample.size | SEM |
| --- | --- | --- | --- | --- | --- | --- | --- |
| shScr | Shapiro-Wilk test | 0.855226848707416 | no | ns | yes | 4 | 1 +/- 0.0311 |
| shDIAPH1 | Shapiro-Wilk test | 0.148572987544409 | no | ns | yes | 4 | 0.4775 +/- 0.0453 |
| shScr H/R | Shapiro-Wilk test | 0.3786662555897 | no | ns | yes | 4 | 1.3925 +/- 0.1085 |
| shDIAPH1 H/R | Shapiro-Wilk test | 0.415290335069471 | no | ns | yes | 4 | 0.37 +/- 0.0626 |

Statistical test for NRF2

| Testing.Category | Group.tested | Method | p | Significant | p. | Sample.size |
| --- | --- | --- | --- | --- | --- | --- |
| Group comparison | Group test | ANOVA | 2.85581714852292e-06 | yes | **** | 4 vs 4 vs 4 vs 4 |
| Pairwise comparison | shScr vs shDIAPH1 | TukeyHSD | 0.000843383337985837 | yes | *** | 4 vs 4 |
| Pairwise comparison | shScr vs shScr H/R | TukeyHSD | 0.143755366635979 | no | ns | 4 vs 4 |
| Pairwise comparison | shScr vs shDIAPH1 H/R | TukeyHSD | 0.000148909721308432 | yes | *** | 4 vs 4 |
| Pairwise comparison | shDIAPH1 vs shScr H/R | TukeyHSD | 2.8621924706318e-05 | yes | **** | 4 vs 4 |
| Pairwise comparison | shDIAPH1 vs shDIAPH1 H/R | TukeyHSD | 0.673970299650393 | no | ns | 4 vs 4 |
| Pairwise comparison | shScr H/R vs shDIAPH1 H/R | TukeyHSD | 6.95511074089161e-06 | yes | **** | 4 vs 4 |

Normality test for NRF2

| Group | Method | p | Significant | p. | Whether.pass | Sample.size | SEM |
| --- | --- | --- | --- | --- | --- | --- | --- |
| shScr | Shapiro-Wilk test | 0.714280151194756 | no | ns | yes | 4 | 1 +/- 0.0274 |
| shDIAPH1 | Shapiro-Wilk test | 0.905730806645054 | no | ns | yes | 4 | 1.4475 +/- 0.0335 |
| shScr H/R | Shapiro-Wilk test | 0.398855897458036 | no | ns | yes | 4 | 0.805 +/- 0.0811 |
| shDIAPH1 H/R | Shapiro-Wilk test | 0.122076234164899 | no | ns | yes | 4 | 1.5425 +/- 0.074 |

Statistical test for PARKIN

| Groups | Statistic.Test | p | Significant | p. | Sample.Size |
| --- | --- | --- | --- | --- | --- |
| shScr H/R vs shDIAPH1 H/R | unpaired t-test | 0.0178746850412273 | yes | * | 4 vs 4 |

Normality test for PARKIN

| Group | Normality.Test | p | Significant | p. | Pass.normality.test. | Sample.Size | SEM |
| --- | --- | --- | --- | --- | --- | --- | --- |
| shScr H/R | Shapiro-Wilk test | 0.395149083369143 | no | ns | yes | 4 | 1.13 +/- 0.0471 |
| shDIAPH1 H/R | Shapiro-Wilk test | 0.295358172172475 | no | ns | yes | 4 | 3.1075 +/- 0.4236 |

Statistical test for PARKIN in group test

| Testing.Category | Group.tested | Method | p | Significant | p. | Sample.size |
| --- | --- | --- | --- | --- | --- | --- |
| Group comparison | Group test | Welch’s ANOVA | 0.000981682156355231 | yes | *** | 4 vs 4 vs 4 vs 4 |
| Pairwise comparison | shScr BL vs shDIAPH1 BL | Games-Howell Test | 0.00868289053106897 | yes | ** | 4 vs 4 |
| Pairwise comparison | shScr BL vs shScr H/R | Games-Howell Test | 0.31624861704111 | no | ns | 4 vs 4 |
| Pairwise comparison | shScr BL vs shDIAPH1 H/R | Games-Howell Test | 0.0446089235911507 | yes | * | 4 vs 4 |
| Pairwise comparison | shDIAPH1 BL vs shScr H/R | Games-Howell Test | 0.0115368319472801 | yes | * | 4 vs 4 |
| Pairwise comparison | shDIAPH1 BL vs shDIAPH1 H/R | Games-Howell Test | 0.747935662319374 | no | ns | 4 vs 4 |
| Pairwise comparison | shScr H/R vs shDIAPH1 H/R | Games-Howell Test | 0.0531169044419649 | no | ns | 4 vs 4 |

Normality test for PARKIN in group test

| Group | Method | p | Significant | p. | Whether.pass | Sample.size | SEM |
| --- | --- | --- | --- | --- | --- | --- | --- |
| shScr BL | Shapiro-Wilk test | 0.880457220879903 | no | ns | yes | 4 | 1 +/- 0.0492 |
| shDIAPH1 BL | Shapiro-Wilk test | 0.91024202038031 | no | ns | yes | 4 | 2.63 +/- 0.1992 |
| shScr H/R | Shapiro-Wilk test | 0.395149083369143 | no | ns | yes | 4 | 1.13 +/- 0.0471 |
| shDIAPH1 H/R | Shapiro-Wilk test | 0.295358172172475 | no | ns | yes | 4 | 3.1075 +/- 0.4236 |

Statistical test for GADD34

| Groups | Statistic.Test | p | Significant | p. | Sample.Size |
| --- | --- | --- | --- | --- | --- |
| shScr H/R vs shDIAPH1 H/R | unpaired t-test | 0.00917129440633854 | yes | ** | 4 vs 4 |

Normality test for GADD34

| Group | Normality.Test | p | Significant | p. | Pass.normality.test. | Sample.Size | SEM |
| --- | --- | --- | --- | --- | --- | --- | --- |
| shScr H/R | Shapiro-Wilk test | 0.542352455786218 | no | ns | yes | 4 | 1.44 +/- 0.1072 |
| shDIAPH1 H/R | Shapiro-Wilk test | 0.677416346457884 | no | ns | yes | 4 | 0.84 +/- 0.0255 |

Statistical test for GADD34 in group test

| Testing.Category | Group.tested | Method | p | Significant | p. | Sample.size |
| --- | --- | --- | --- | --- | --- | --- |
| Group comparison | Group test | Welch’s ANOVA | 0.00403039945456765 | yes | ** | 4 vs 4 vs 4 vs 4 |
| Pairwise comparison | shScr BL vs shDIAPH1 BL | Games-Howell Test | 0.143887496143254 | no | ns | 4 vs 4 |
| Pairwise comparison | shScr BL vs shScr H/R | Games-Howell Test | 0.0717814953848556 | no | ns | 4 vs 4 |
| Pairwise comparison | shScr BL vs shDIAPH1 H/R | Games-Howell Test | 0.0122511459272286 | yes | * | 4 vs 4 |
| Pairwise comparison | shDIAPH1 BL vs shScr H/R | Games-Howell Test | 0.0170488645186444 | yes | * | 4 vs 4 |
| Pairwise comparison | shDIAPH1 BL vs shDIAPH1 H/R | Games-Howell Test | 0.987815387484973 | no | ns | 4 vs 4 |
| Pairwise comparison | shScr H/R vs shDIAPH1 H/R | Games-Howell Test | 0.0286517100509861 | yes | * | 4 vs 4 |

Normality test for GADD34 in group test

| Group | Method | p | Significant | p. | Whether.pass | Sample.size | SEM |
| --- | --- | --- | --- | --- | --- | --- | --- |
| shScr BL | Shapiro-Wilk test | 0.995064018830953 | no | ns | yes | 4 | 1 +/- 0.0208 |
| shDIAPH1 BL | Shapiro-Wilk test | 0.216482874981762 | no | ns | yes | 4 | 0.82 +/- 0.0585 |
| shScr H/R | Shapiro-Wilk test | 0.542352455786218 | no | ns | yes | 4 | 1.44 +/- 0.1072 |
| shDIAPH1 H/R | Shapiro-Wilk test | 0.677416346457884 | no | ns | yes | 4 | 0.84 +/- 0.0255 |

Statistical test for TOMM40

| Testing.Category | Group.tested | Method | p | Significant | p. | Sample.size |
| --- | --- | --- | --- | --- | --- | --- |
| Group comparison | Group test | ANOVA | 0.522142289800444 | no | ns | 4 vs 4 vs 4 vs 4 |
| Pairwise comparison | shScr vs shDIAPH1 | TukeyHSD | 0.97249758244316 | no | ns | 4 vs 4 |
| Pairwise comparison | shScr vs shScr H/R | TukeyHSD | 0.488464584106909 | no | ns | 4 vs 4 |
| Pairwise comparison | shScr vs shDIAPH1 H/R | TukeyHSD | 0.801601442760881 | no | ns | 4 vs 4 |
| Pairwise comparison | shDIAPH1 vs shScr H/R | TukeyHSD | 0.734196660369614 | no | ns | 4 vs 4 |
| Pairwise comparison | shDIAPH1 vs shDIAPH1 H/R | TukeyHSD | 0.962389189795654 | no | ns | 4 vs 4 |
| Pairwise comparison | shScr H/R vs shDIAPH1 H/R | TukeyHSD | 0.94361613817342 | no | ns | 4 vs 4 |

Normality test for TOMM40

| Group | Method | p | Significant | p. | Whether.pass | Sample.size | SEM |
| --- | --- | --- | --- | --- | --- | --- | --- |
| shScr | Shapiro-Wilk test | 0.682961531589327 | no | ns | yes | 4 | 1 +/- 0.0327 |
| shDIAPH1 | Shapiro-Wilk test | 0.244635345383794 | no | ns | yes | 4 | 0.9575 +/- 0.0545 |
| shScr H/R | Shapiro-Wilk test | 0.314416001107091 | no | ns | yes | 4 | 0.855 +/- 0.047 |
| shDIAPH1 H/R | Shapiro-Wilk test | 0.731768388746701 | no | ns | yes | 4 | 0.91 +/- 0.116 |

Statistical test for BCL2

| Testing.Category | Group.tested | Method | p | Significant | p. | Sample.size |
| --- | --- | --- | --- | --- | --- | --- |
| Group comparison | Group test | ANOVA | 0.232444303459296 | no | ns | 4 vs 4 vs 4 vs 4 |
| Pairwise comparison | shScr vs shDIAPH1 | TukeyHSD | 0.998162761228018 | no | ns | 4 vs 4 |
| Pairwise comparison | shScr vs shScr H/R | TukeyHSD | 0.351801383887032 | no | ns | 4 vs 4 |
| Pairwise comparison | shScr vs shDIAPH1 H/R | TukeyHSD | 0.435688474307597 | no | ns | 4 vs 4 |
| Pairwise comparison | shDIAPH1 vs shScr H/R | TukeyHSD | 0.435688474307597 | no | ns | 4 vs 4 |
| Pairwise comparison | shDIAPH1 vs shDIAPH1 H/R | TukeyHSD | 0.528669185729195 | no | ns | 4 vs 4 |
| Pairwise comparison | shScr H/R vs shDIAPH1 H/R | TukeyHSD | 0.998162761228018 | no | ns | 4 vs 4 |

Normality test for BCL2

| Group | Method | p | Significant | p. | Whether.pass | Sample.size | SEM |
| --- | --- | --- | --- | --- | --- | --- | --- |
| shScr | Shapiro-Wilk test | 0.867795446298398 | no | ns | yes | 4 | 1 +/- 0.0842 |
| shDIAPH1 | Shapiro-Wilk test | 0.327167540927158 | no | ns | yes | 4 | 1.04 +/- 0.184 |
| shScr H/R | Shapiro-Wilk test | 0.146302746675912 | no | ns | yes | 4 | 1.4075 +/- 0.2158 |
| shDIAPH1 H/R | Shapiro-Wilk test | 0.292477291661911 | no | ns | yes | 4 | 1.3675 +/- 0.1536 |

Statistical test for NEFL

| Testing.Category | Group.tested | Method | p | Significant | p. | Sample.size |
| --- | --- | --- | --- | --- | --- | --- |
| Group comparison | Group test | ANOVA | 0.312655536124494 | no | ns | 4 vs 4 vs 4 vs 4 |
| Pairwise comparison | shScr vs shDIAPH1 | TukeyHSD | 0.412963805774649 | no | ns | 4 vs 4 |
| Pairwise comparison | shScr vs shScr H/R | TukeyHSD | 0.360170404252992 | no | ns | 4 vs 4 |
| Pairwise comparison | shScr vs shDIAPH1 H/R | TukeyHSD | 0.909972448219484 | no | ns | 4 vs 4 |
| Pairwise comparison | shDIAPH1 vs shScr H/R | TukeyHSD | 0.999525635562041 | no | ns | 4 vs 4 |
| Pairwise comparison | shDIAPH1 vs shDIAPH1 H/R | TukeyHSD | 0.782924725481693 | no | ns | 4 vs 4 |
| Pairwise comparison | shScr H/R vs shDIAPH1 H/R | TukeyHSD | 0.7241928410503 | no | ns | 4 vs 4 |

Normality test for NEFL

| Group | Method | p | Significant | p. | Whether.pass | Sample.size | SEM |
| --- | --- | --- | --- | --- | --- | --- | --- |
| shScr | Shapiro-Wilk test | 0.79670553781676 | no | ns | yes | 4 | 1 +/- 0.094 |
| shDIAPH1 | Shapiro-Wilk test | 0.637790860597065 | no | ns | yes | 4 | 1.2975 +/- 0.1335 |
| shScr H/R | Shapiro-Wilk test | 0.421344806101302 | no | ns | yes | 4 | 1.3175 +/- 0.1324 |
| shDIAPH1 H/R | Shapiro-Wilk test | 0.499907030090132 | no | ns | yes | 4 | 1.1225 +/- 0.1572 |

Statistical test for PERK

| Testing.Category | Group.tested | Method | p | Significant | p. | Sample.size |
| --- | --- | --- | --- | --- | --- | --- |
| Group comparison | Group test | Kruskal-Wallis test | 0.0670410668365629 | no | ns | 4 vs 4 vs 4 vs 4 |
| Pairwise comparison | shScr vs shDIAPH1 | Dunn’s Test | 0.271499626208064 | no | ns | 4 vs 4 |
| Pairwise comparison | shScr vs shScr H/R | Dunn’s Test | 0.0773475703558007 | no | ns | 4 vs 4 |
| Pairwise comparison | shScr vs shDIAPH1 H/R | Dunn’s Test | 1 | no | ns | 4 vs 4 |
| Pairwise comparison | shDIAPH1 vs shScr H/R | Dunn’s Test | 0.447007402894507 | no | ns | 4 vs 4 |
| Pairwise comparison | shDIAPH1 vs shDIAPH1 H/R | Dunn’s Test | 0.271499626208064 | no | ns | 4 vs 4 |
| Pairwise comparison | shScr H/R vs shDIAPH1 H/R | Dunn’s Test | 0.0773475703558007 | no | ns | 4 vs 4 |

Normality test for PERK

| Group | Method | p | Significant | p. | Whether.pass | Sample.size | SEM |
| --- | --- | --- | --- | --- | --- | --- | --- |
| shScr | Shapiro-Wilk test | 0.792387304427384 | no | ns | yes | 4 | 1 +/- 0.0981 |
| shDIAPH1 | Shapiro-Wilk test | 0.00254701817577942 | yes | ** | no | 4 | 1.1975 +/- 0.0892 |
| shScr H/R | Shapiro-Wilk test | 0.802969701159486 | no | ns | yes | 4 | 1.435 +/- 0.1518 |
| shDIAPH1 H/R | Shapiro-Wilk test | 0.347049951701163 | no | ns | yes | 4 | 1.0025 +/- 0.1251 |

Statistical test for EDEM1

| Testing.Category | Group.tested | Method | p | Significant | p. | Sample.size |
| --- | --- | --- | --- | --- | --- | --- |
| Group comparison | Group test | Kruskal-Wallis test | 0.0323168574068213 | yes | * | 4 vs 4 vs 4 vs 4 |
| Pairwise comparison | shScr vs shDIAPH1 | Dunn’s Test | 0.0620510889557538 | no | ns | 4 vs 4 |
| Pairwise comparison | shScr vs shScr H/R | Dunn’s Test | 0.0620510889557538 | no | ns | 4 vs 4 |
| Pairwise comparison | shScr vs shDIAPH1 H/R | Dunn’s Test | 0.044525767727199 | yes | * | 4 vs 4 |
| Pairwise comparison | shDIAPH1 vs shScr H/R | Dunn’s Test | 0.881757984608166 | no | ns | 4 vs 4 |
| Pairwise comparison | shDIAPH1 vs shDIAPH1 H/R | Dunn’s Test | 0.852003141610138 | no | ns | 4 vs 4 |
| Pairwise comparison | shScr H/R vs shDIAPH1 H/R | Dunn’s Test | 0.852003141610138 | no | ns | 4 vs 4 |

Normality test for EDEM1

| Group | Method | p | Significant | p. | Whether.pass | Sample.size | SEM |
| --- | --- | --- | --- | --- | --- | --- | --- |
| shScr | Shapiro-Wilk test | 0.0238567944022221 | yes | * | no | 4 | 1 +/- 0.0231 |
| shDIAPH1 | Shapiro-Wilk test | 0.519119427547098 | no | ns | yes | 4 | 1.3925 +/- 0.1517 |
| shScr H/R | Shapiro-Wilk test | 0.0574372157728044 | no | ns | yes | 4 | 1.31 +/- 0.125 |
| shDIAPH1 H/R | Shapiro-Wilk test | 0.211686065375044 | no | ns | yes | 4 | 1.365 +/- 0.1121 |

Statistical test for PINK1

| Testing.Category | Group.tested | Method | p | Significant | p. | Sample.size |
| --- | --- | --- | --- | --- | --- | --- |
| Group comparison | Group test | ANOVA | 3.27758250935506e-05 | yes | **** | 4 vs 4 vs 4 vs 4 |
| Pairwise comparison | shScr vs shDIAPH1 | TukeyHSD | 0.000320240174470743 | yes | *** | 4 vs 4 |
| Pairwise comparison | shScr vs shScr H/R | TukeyHSD | 0.986496508661187 | no | ns | 4 vs 4 |
| Pairwise comparison | shScr vs shDIAPH1 H/R | TukeyHSD | 0.000342159205036996 | yes | *** | 4 vs 4 |
| Pairwise comparison | shDIAPH1 vs shScr H/R | TukeyHSD | 0.000535344770741131 | yes | *** | 4 vs 4 |
| Pairwise comparison | shDIAPH1 vs shDIAPH1 H/R | TukeyHSD | 0.999968467975964 | no | ns | 4 vs 4 |
| Pairwise comparison | shScr H/R vs shDIAPH1 H/R | TukeyHSD | 0.000573062097057431 | yes | *** | 4 vs 4 |

Normality test for PINK1

| Group | Method | p | Significant | p. | Whether.pass | Sample.size | SEM |
| --- | --- | --- | --- | --- | --- | --- | --- |
| shScr | Shapiro-Wilk test | 0.463049046890851 | no | ns | yes | 4 | 1 +/- 0.0861 |
| shDIAPH1 | Shapiro-Wilk test | 0.669156423957709 | no | ns | yes | 4 | 2.03 +/- 0.1185 |
| shScr H/R | Shapiro-Wilk test | 0.708718650563067 | no | ns | yes | 4 | 1.0575 +/- 0.0595 |
| shDIAPH1 H/R | Shapiro-Wilk test | 0.188163193071939 | no | ns | yes | 4 | 2.0225 +/- 0.1849 |

Statistical test for MFN2

| Testing.Category | Group.tested | Method | p | Significant | p. | Sample.size |
| --- | --- | --- | --- | --- | --- | --- |
| Group comparison | Group test | Kruskal-Wallis test | 0.0128829530056516 | yes | * | 4 vs 4 vs 4 vs 4 |
| Pairwise comparison | shScr vs shDIAPH1 | Dunn’s Test | 0.0571730445477902 | no | ns | 4 vs 4 |
| Pairwise comparison | shScr vs shScr H/R | Dunn’s Test | 0.54806723770097 | no | ns | 4 vs 4 |
| Pairwise comparison | shScr vs shDIAPH1 H/R | Dunn’s Test | 0.0249906481527193 | yes | * | 4 vs 4 |
| Pairwise comparison | shDIAPH1 vs shScr H/R | Dunn’s Test | 0.164349114541414 | no | ns | 4 vs 4 |
| Pairwise comparison | shDIAPH1 vs shDIAPH1 H/R | Dunn’s Test | 0.60238213445829 | no | ns | 4 vs 4 |
| Pairwise comparison | shScr H/R vs shDIAPH1 H/R | Dunn’s Test | 0.0678226540088648 | no | ns | 4 vs 4 |

Normality test for MFN2

| Group | Method | p | Significant | p. | Whether.pass | Sample.size | SEM |
| --- | --- | --- | --- | --- | --- | --- | --- |
| shScr | Shapiro-Wilk test | 0.0238567944022221 | yes | * | no | 4 | 1 +/- 0.0058 |
| shDIAPH1 | Shapiro-Wilk test | 0.796641805691313 | no | ns | yes | 4 | 1.31 +/- 0.0455 |
| shScr H/R | Shapiro-Wilk test | 0.425109868823983 | no | ns | yes | 4 | 1.08 +/- 0.057 |
| shDIAPH1 H/R | Shapiro-Wilk test | 0.358769657623702 | no | ns | yes | 4 | 1.5125 +/- 0.137 |

Statistical test for DRP1

| Testing.Category | Group.tested | Method | p | Significant | p. | Sample.size |
| --- | --- | --- | --- | --- | --- | --- |
| Group comparison | Group test | ANOVA | 0.38239971084081 | no | ns | 4 vs 4 vs 4 vs 4 |
| Pairwise comparison | shScr vs shDIAPH1 | TukeyHSD | 0.360371156524638 | no | ns | 4 vs 4 |
| Pairwise comparison | shScr vs H/R shScr | TukeyHSD | 0.881990060825324 | no | ns | 4 vs 4 |
| Pairwise comparison | shScr vs H/R shDIAPH1 | TukeyHSD | 0.565261110031505 | no | ns | 4 vs 4 |
| Pairwise comparison | shDIAPH1 vs H/R shScr | TukeyHSD | 0.764614931513685 | no | ns | 4 vs 4 |
| Pairwise comparison | shDIAPH1 vs H/R shDIAPH1 | TukeyHSD | 0.979719545838416 | no | ns | 4 vs 4 |
| Pairwise comparison | H/R shScr vs H/R shDIAPH1 | TukeyHSD | 0.932421987928102 | no | ns | 4 vs 4 |

Normality test for DRP1

| Group | Method | p | Significant | p. | Whether.pass | Sample.size | SEM |
| --- | --- | --- | --- | --- | --- | --- | --- |
| shScr | Shapiro-Wilk test | 0.765565361012498 | no | ns | yes | 4 | 1 +/- 0.2655 |
| shDIAPH1 | Shapiro-Wilk test | 0.84383166992611 | no | ns | yes | 4 | 1.7 +/- 0.2462 |
| H/R shScr | Shapiro-Wilk test | 0.912902614850545 | no | ns | yes | 4 | 1.3 +/- 0.2801 |
| H/R shDIAPH1 | Shapiro-Wilk test | 0.376384679153256 | no | ns | yes | 4 | 1.5425 +/- 0.3542 |

## Figure 4

Statistical test for Mito velocity 4a

| Groups | Statistic.Test | p | Significant | p. | Sample.Size |
| --- | --- | --- | --- | --- | --- |
| shScr vs shDIAPH1 | wilcoxon rank-sum test | 6.46537935627394e-05 | yes | **** | 35 vs 39 |

Normality test for Mito velocity

| Group | Normality.Test | p | Significant | p. | Pass.normality.test. | Sample.Size | SEM |
| --- | --- | --- | --- | --- | --- | --- | --- |
| shScr | Shapiro-Wilk test | 0.012500147729823 | yes | * | no | 35 | 0.1412 +/- 0.0157 |
| shDIAPH1 | Shapiro-Wilk test | 1.83920179941751e-05 | yes | **** | no | 39 | 0.2668 +/- 0.0268 |

Statistical test for fig4_b Citrate Synthase

| Testing.Category | Group.tested | Method | p | Significant | p. | Sample.size |
| --- | --- | --- | --- | --- | --- | --- |
| Group comparison | Group test | ANOVA | 0.000433245258293293 | yes | *** | 4 vs 4 vs 4 vs 4 |
| Pairwise comparison | shScr vs shDIAPH1 | TukeyHSD | 0.000980648416943786 | yes | *** | 4 vs 4 |
| Pairwise comparison | shScr vs shScr H/R | TukeyHSD | 0.417655082626529 | no | ns | 4 vs 4 |
| Pairwise comparison | shScr vs shDIAPH1 H/R | TukeyHSD | 0.00187926395798532 | yes | ** | 4 vs 4 |
| Pairwise comparison | shDIAPH1 vs shScr H/R | TukeyHSD | 0.0145444591515639 | yes | * | 4 vs 4 |
| Pairwise comparison | shDIAPH1 vs shDIAPH1 H/R | TukeyHSD | 0.977578090106807 | no | ns | 4 vs 4 |
| Pairwise comparison | shScr H/R vs shDIAPH1 H/R | TukeyHSD | 0.0292421807065396 | yes | * | 4 vs 4 |

Normality test for fig4_b Citrate Synthase

| Group | Method | p | Significant | p. | Whether.pass | Sample.size | SEM |
| --- | --- | --- | --- | --- | --- | --- | --- |
| shScr | Shapiro-Wilk test | 0.336292553256181 | no | ns | yes | 4 | 1e-04 +/- 0 |
| shDIAPH1 | Shapiro-Wilk test | 0.997104870302609 | no | ns | yes | 4 | 2e-04 +/- 0 |
| shScr H/R | Shapiro-Wilk test | 0.299745061590981 | no | ns | yes | 4 | 1e-04 +/- 0 |
| shDIAPH1 H/R | Shapiro-Wilk test | 0.243343848517121 | no | ns | yes | 4 | 1e-04 +/- 0 |
|  |  |  |  |  |  |  |  |

Statistical test for Fig 4c MitoSOX

shScr H/R vs shDIAPH1 H/R unpaired t-test 0.000126125105048741 yes *** 4 vs 4

Normality test for Fig 4c MitoSOX

ShScr H/R Shapiro-Wilk test 0.209785105312845 no ns yes 4 9.728 +/- 0.3764

shDIAPH1 H/R Shapiro-Wilk test 0.333691683877995 no ns yes 4 6.2177 +/- 0.1432

Statistical test for fig4_d MPTP

| Groups | Statistic.Test | p | Significant | p. | Sample.Size |
| --- | --- | --- | --- | --- | --- |
| shScr H/R 1 vs shScr H/R 2 | one-tailed wilcoxon rank-sum test | 0.0285714285714286 | yes | * | 4 vs 4 |

Normality test for fig4_d MPTP

| Group | Normality.Test | p | Significant | p. | Pass.normality.test. | Sample.Size | SEM |
| --- | --- | --- | --- | --- | --- | --- | --- |
| shScr H/R 1 | Shapiro-Wilk test | 0.448333191748053 | no | ns | yes | 4 | 32.318 +/- 6.7474 |
| shScr H/R 2 | Shapiro-Wilk test | 0.00418178763040334 | yes | ** | no | 4 | 12.604 +/- 3.2428 |

Statistical test for fig4_e MitoPT

| Groups | Statistic.Test | p | Significant | p. | Sample.Size |
| --- | --- | --- | --- | --- | --- |
| shScr H/R vs shDIAPH1 H/R | unpaired t-test | 0.0121206346659854 | yes | * | 4 vs 4 |

Normality test for fig4_e MitoPT

| Group | Normality.Test | p | Significant | p. | Pass.normality.test. | Sample.Size | SEM |
| --- | --- | --- | --- | --- | --- | --- | --- |
| shScr H/R | Shapiro-Wilk test | 0.354500099394962 | no | ns | yes | 4 | 1.0629 +/- 0.0332 |
| shDIAPH1 H/R | Shapiro-Wilk test | 0.161116399639464 | no | ns | yes | 4 | 1.5366 +/- 0.1294 |

Statistical test for fig4_f MitoT in HipSC-CMs

| Testing.Category | Group.tested | Method | p | Significant | p. | Sample.size |
| --- | --- | --- | --- | --- | --- | --- |
| Group comparison | Group test | Kruskal-Wallis test | 0.0130282881015742 | yes | * | 5 vs 5 vs 5 vs 5 |
| Pairwise comparison | shScr vs shDIAPH1 | Dunn’s Test | 0.957371576490613 | no | ns | 5 vs 5 |
| Pairwise comparison | shScr vs shScr H/R | Dunn’s Test | 0.0203228657657551 | no | * | 5 vs 5 |
| Pairwise comparison | shScr vs shDIAPH1 H/R | Dunn’s Test | 0.436217667283733 | no | ns | 5 vs 5 |
| Pairwise comparison | shDIAPH1 vs shScr H/R | Dunn’s Test | 0.0568251611992847 | no | ns | 5 vs 5 |
| Pairwise comparison | shDIAPH1 vs shDIAPH1 H/R | Dunn’s Test | 0.436217667283733 | no | ns | 5 vs 5 |
| Pairwise comparison | shScr H/R vs shDIAPH1 H/R | Dunn’s Test | 0.00967345803343743 | yes | ** | 5 vs 5 |

Normality test for fig4_f MitoT in HipSC-CMs

| Group | Method | p | Significant | p. | Whether.pass | Sample.size | SEM |
| --- | --- | --- | --- | --- | --- | --- | --- |
| shScr | Shapiro-Wilk test | 0.0263531425809151 | yes | * | no | 5 | 8.3394 +/- 1.9551 |
| shDIAPH1 | Shapiro-Wilk test | 0.109187326107628 | no | ns | yes | 5 | 9.3056 +/- 3.1585 |
| shScr H/R | Shapiro-Wilk test | 0.451870285646936 | no | ns | yes | 5 | 1.9367 +/- 0.4482 |
| shDIAPH1 H/R | Shapiro-Wilk test | 0.391502300577632 | no | ns | yes | 5 | 22.7489 +/- 7.5664 |

Statistical test for fig4_g Basal mito respiration

| Groups | Statistic.Test | p | Significant | p. | Sample.Size |
| --- | --- | --- | --- | --- | --- |
| shScr H/R vs shDIAPH1 H/R | Welch’s unpaired t test | 0.0046 | yes | ** | 6 vs 6 |

Statistical test for fig4_g ATP production

| Groups | Statistic.Test | p | Significant | p. | Sample.Size |
| --- | --- | --- | --- | --- | --- |
| shScr H/R vs shDIAPH1 H/R | Welch’s unpaired t test | 0.0010 | yes | ** | 6 vs 6 |

Statistical test for fig4_h Annexin_V

| Groups | Statistic.Test | p | Significant | p. | Sample.Size |
| --- | --- | --- | --- | --- | --- |
| shScr H/R vs shDiaph1 H/R | unpaired t-test | 0.0181264569445671 | yes | * | 4 vs 4 |

Normality test for Annexin_V

| Group | Normality.Test | p | Significant | p. | Pass.normality.test. | Sample.Size | SEM |
| --- | --- | --- | --- | --- | --- | --- | --- |
| shScr H/R | Shapiro-Wilk test | 0.161190614218768 | no | ns | yes | 4 | 0.007 +/- 7e-04 |
| shDiaph1 H/R | Shapiro-Wilk test | 0.272453161730903 | no | ns | yes | 4 | 0.0043 +/- 5e-04 |

Statistical test for fig4_hi_ER_ID

| Testing.Category | Group.tested | Method | p | Significant | p. | Sample.size |
| --- | --- | --- | --- | --- | --- | --- |
| Pairwise comparison | shScr H/R vs shDIAPH1 H/R | TukeyHSD | 0.00732648045203799 | yes | ** | 4 vs 4 |

Normality test for Rev_Figure_ER_ID

| Group | Method | p | Significant | p. | Whether.pass | Sample.size | SEM |
| --- | --- | --- | --- | --- | --- | --- | --- |
| shScr H/R | Shapiro-Wilk test | 0.273593363191759 | no | ns | yes | 4 | 0.4119 +/- 0.0271 |
| shDIAPH1 H/R | Shapiro-Wilk test | 0.14351215702039 | no | ns | yes | 4 | 0.2987 +/- 0.0161 |

Statistical test for fig4_j fluo4

| Groups | Statistic.Test | p | Significant | p. | Sample.Size |
| --- | --- | --- | --- | --- | --- |
| shScr H/R vs shDiaph1 H/R | unpaired t-test | 0.0314164959242783 | yes | * | 6 vs 6 |

Normality test for fluo4

| Group | Normality.Test | p | Significant | p. | Pass.normality.test. | Sample.Size | SEM |
| --- | --- | --- | --- | --- | --- | --- | --- |
| shScr H/R | Shapiro-Wilk test | 0.06825018467896 | no | ns | yes | 6 | 530.3333 +/- 61.7693 |
| shDiaph1 H/R | Shapiro-Wilk test | 0.554784400601621 | no | ns | yes | 6 | 810 +/- 93.2366 |

Statistical test for Mag_fluo4

| Groups | Statistic.Test | p | Significant | p. | Sample.Size |
| --- | --- | --- | --- | --- | --- |
| shScr H/R vs shDiaph1 H/R | unpaired t-test | 5.47013395916542e-05 | yes | **** | 8 vs 8 |

Normality test for Mag_fluo4

| Group | Normality.Test | p | Significant | p. | Pass.normality.test. | Sample.Size | SEM |
| --- | --- | --- | --- | --- | --- | --- | --- |
| shScr H/R | Shapiro-Wilk test | 0.421704562881857 | no | ns | yes | 8 | 157.875 +/- 5.674 |
| shDiaph1 H/R | Shapiro-Wilk test | 0.62581309969019 | no | ns | yes | 8 | 270.5 +/- 14.7648 |

Statistical test for fig4_k_Phosphatidylcholine

| Testing.Category | Group.tested | Method | p | Significant | p. | Sample.size |
| --- | --- | --- | --- | --- | --- | --- |
| Group comparison | Group test | Welch’s ANOVA | 1.99883132375072e-06 | yes | **** | 4 vs 4 vs 4 vs 4 |
| Pairwise comparison | shScr BL vs shDIAPH1 BL | Games-Howell Test | 8.10011936290822e-05 | yes | **** | 4 vs 4 |
| Pairwise comparison | shScr BL vs shScr H/R | Games-Howell Test | 0.0494163539241861 | yes | * | 4 vs 4 |
| Pairwise comparison | shScr BL vs shDIAPH1 H/R | Games-Howell Test | 0.000416168922846349 | yes | *** | 4 vs 4 |
| Pairwise comparison | shDIAPH1 BL vs shScr H/R | Games-Howell Test | 0.000297955759991808 | yes | *** | 4 vs 4 |
| Pairwise comparison | shDIAPH1 BL vs shDIAPH1 H/R | Games-Howell Test | 0.00995595888712808 | yes | ** | 4 vs 4 |
| Pairwise comparison | shScr H/R vs shDIAPH1 H/R | Games-Howell Test | 0.000515170094709028 | yes | *** | 4 vs 4 |

Normality test for fig4_k Phosphatidylcholine

| Group | Method | p | Significant | p. | Whether.pass | Sample.size | SEM |
| --- | --- | --- | --- | --- | --- | --- | --- |
| shScr BL | Shapiro-Wilk test | 0.415637907517265 | no | ns | yes | 4 | 65.4731 +/- 1.1977 |
| shDIAPH1 BL | Shapiro-Wilk test | 0.676677366862564 | no | ns | yes | 4 | 95.5884 +/- 0.5218 |
| shScr H/R | Shapiro-Wilk test | 0.961116863934544 | no | ns | yes | 4 | 71.6769 +/- 1.319 |
| shDIAPH1 H/R | Shapiro-Wilk test | 0.72035594162687 | no | ns | yes | 4 | 122.768 +/- 3.292 |

Statistical test for _PhosphatidylSerine

| Testing.Category | Group.tested | Method | p | Significant | p. | Sample.size |
| --- | --- | --- | --- | --- | --- | --- |
| Group comparison | Group test | ANOVA | 2.51748368347763e-05 | yes | **** | 4 vs 4 vs 4 vs 4 |
| Pairwise comparison | shScr BL vs shDIAPH1 BL | TukeyHSD | 0.000155590363927871 | yes | *** | 4 vs 4 |
| Pairwise comparison | shScr BL vs shScr H/R | TukeyHSD | 0.982270180712332 | no | ns | 4 vs 4 |
| Pairwise comparison | shScr BL vs shDIAPH1 H/R | TukeyHSD | 0.000458865881856618 | yes | *** | 4 vs 4 |
| Pairwise comparison | shDIAPH1 BL vs shScr H/R | TukeyHSD | 0.000267643568741316 | yes | *** | 4 vs 4 |
| Pairwise comparison | shDIAPH1 BL vs shDIAPH1 H/R | TukeyHSD | 0.886823590372871 | no | ns | 4 vs 4 |
| Pairwise comparison | shScr H/R vs shDIAPH1 H/R | TukeyHSD | 0.000816705485775504 | yes | *** | 4 vs 4 |

Normality test for

PhosphatidylSerine

| Group | Method | p | Significant | p. | Whether.pass | Sample.size | SEM |
| --- | --- | --- | --- | --- | --- | --- | --- |
| shScr BL | Shapiro-Wilk test | 0.286556297860547 | no | ns | yes | 4 | 18.3549 +/- 1.5557 |
| shDIAPH1 BL | Shapiro-Wilk test | 0.647973496911151 | no | ns | yes | 4 | 41.5098 +/- 3.9316 |
| shScr H/R | Shapiro-Wilk test | 0.0796685414034618 | no | ns | yes | 4 | 19.6687 +/- 1.238 |
| shDIAPH1 H/R | Shapiro-Wilk test | 0.732171974218566 | no | ns | yes | 4 | 38.9301 +/- 2.4875 |

## Supplement figure 9 supporting Figure 4

Statistical test for MitoT in Mice hearts

| Testing.Category | Group.tested | Method | p | Significant | p. | Sample.size |
| --- | --- | --- | --- | --- | --- | --- |
| Group comparison | Group test | Welch’s ANOVA | 2.01827905831281e-07 | yes | **** | 6 vs 6 vs 5 vs 6 |
| Pairwise comparison | WT-BL vs DKO-BL | t-test with pooled SD | 0.000285404645522659 | yes | *** | 6 vs 6 |
| Pairwise comparison | WT-BL vs WT-IR | t-test with pooled SD | 0.00396200405574355 | yes | ** | 6 vs 5 |
| Pairwise comparison | WT-BL vs DKO-IR | t-test with pooled SD | 3.69106858958039e-06 | yes | **** | 6 vs 6 |
| Pairwise comparison | DKO-BL vs WT-IR | t-test with pooled SD | 0.232295507872584 | no | ns | 6 vs 5 |
| Pairwise comparison | DKO-BL vs DKO-IR | t-test with pooled SD | 0.0323897300672641 | yes | * | 6 vs 6 |
| Pairwise comparison | WT-IR vs DKO-IR | t-test with pooled SD | 0.00396200405574355 | yes | ** | 5 vs 6 |

Normality test for fig3_h MitoT in Mice hearts

| Group | Method | p | Significant | p. | Whether.pass | Sample.size | SEM |
| --- | --- | --- | --- | --- | --- | --- | --- |
| WT-BL | Shapiro-Wilk test | 0.2743287003069 | no | ns | yes | 6 | 0.4911 +/- 0.0551 |
| DKO-BL | Shapiro-Wilk test | 0.525439395129429 | no | ns | yes | 6 | 2.1859 +/- 0.1883 |
| WT-IR | Shapiro-Wilk test | 0.724351316573911 | no | ns | yes | 5 | 1.7401 +/- 0.0574 |
| DKO-IR | Shapiro-Wilk test | 0.130754005057525 | no | ns | yes | 6 | 3.0116 +/- 0.4299 |

## Figure 5

*Statistical test for figure5*

| Testing.Category | Group.tested | Method | p | Significant | p. | Sample.size |
| --- | --- | --- | --- | --- | --- | --- |
|  |  |  |  |  |  |  |
| Fig 5b Infacrt size-cmDKO | WT vs CM-DKO | unpaired t-test | 0.00558282812980728 | yes | ** | 6 vs 6 |
| Fig 5c preecho-cmDKO | WT vs CM-DKO | unpaired t-test | 0.979122824181054 | no | ns | 6 vs 6 |
| Fig 5d postecho-cmDKO | WT vs CM-DKO | unpaired t-test | 0.00722707571343848 | yes | ** | 6 vs 6 |
| Fig 5e preecho-FS-cmDKO | WT vs CM-DKO | wilcoxon rank-sum test | 0.588744588744589 | no | ns | 6 vs 6 |
| Fig 5f postecho-FS-cmDKO | WT vs CM-DKO | unpaired t-test | 0.00308473929254622 | yes | ** | 6 vs 6 |
|  |  |  |  |  |  |  |
|  |  |  |  |  |  |  |
|  |  |  |  |  |  |  |

## *Normality test for figure 5*

| Fig | Group | Method | p | Significant | p. | Whether.pass | Sample.size | SEM |
| --- | --- | --- | --- | --- | --- | --- | --- | --- |
|  |  |  |  |  |  |  |  |  |
|  |  |  |  |  |  |  |  |  |
| Fig 5b Infacrt size-cmDKO | WT | Shapiro-Wilk test | 0.745972100958196 | no | ns | yes | 6 | 42.1687 +/- 2.2998 |
| Fig 5b Infacrt size-cmDKO | CM-DKO | Shapiro-Wilk test | 0.189303383886847 | no | ns | yes | 6 | 25.426 +/- 4.1709 |
|  |  |  |  |  |  |  |  |  |
|  |  |  |  |  |  |  |  |  |
|  |  |  |  |  |  |  |  |  |
| Fig 5c preecho-cmDKO | WT | Shapiro-Wilk test | 0.259461140342492 | no | ns | yes | 6 | 64.3198 +/- 0.901 |
| Fig 5c preecho-cmDKO | CM-DKO | Shapiro-Wilk test | 0.591445081234778 | no | ns | yes | 6 | 64.2816 +/- 1.1012 |
| Fig 5d postecho-cmDKO | WT | Shapiro-Wilk test | 0.0581932047737757 | no | ns | yes | 6 | 49.6016 +/- 3.085 |
| Fig 5d postecho-cmDKO | CM-DKO | Shapiro-Wilk test | 0.477846707168505 | no | ns | yes | 6 | 62.5005 +/- 0.9899 |
| Fig 5e preecho-FS-cmDKO | WT | Shapiro-Wilk test | 0.0347388217746834 | yes | * | no | 6 | 34.3493 +/- 1.6953 |
| Fig 5e preecho-FS-cmDKO | CM-DKO | Shapiro-Wilk test | 0.858028298344843 | no | ns | yes | 6 | 36.3908 +/- 1.6387 |
| Fig 5f postecho-FS-cmDKO | WT | Shapiro-Wilk test | 0.648662271056347 | no | ns | yes | 6 | 24.0826 +/- 1.5163 |
| Fig 5f postecho-FS-cmDKO | CM-DKO | Shapiro-Wilk test | 0.967909756148621 | no | ns | yes | 6 | 32.947 +/- 1.713 |
|  |  |  |  |  |  |  |  |  |
|  |  |  |  |  |  |  |  |  |

## Supplementary figure 10 supporting figure 5

Statistical test for figS1 b Cardiomyocytes

| Groups | Statistic.Test | p | Significant | p. | Sample.Size |
| --- | --- | --- | --- | --- | --- |
| WT vs CM-DKO | unpaired t-test | 1.08196295219463e-08 | yes | **** | 7 vs 7 |

Normality test for figS1 b Cardiomyocytes

| Group | Normality.Test | p | Significant | p. | Pass.normality.test. | Sample.Size | SEM |
| --- | --- | --- | --- | --- | --- | --- | --- |
| WT | Shapiro-Wilk test | 0.308869444341708 | no | ns | yes | 7 | 1.0481 +/- 0.0414 |
| CM-DKO | Shapiro-Wilk test | 0.0762347954148945 | no | ns | yes | 7 | 0.3427 +/- 0.0306 |

Statistical test for figS1 b kidney

| Groups | Statistic.Test | p | Significant | p. | Sample.Size |
| --- | --- | --- | --- | --- | --- |
| WT vs CM-DKO | unpaired t-test | 0.641486077832861 | no | ns | 5 vs 5 |

Normality test for figS1 b kidney

| Group | Normality.Test | p | Significant | p. | Pass.normality.test. | Sample.Size | SEM |
| --- | --- | --- | --- | --- | --- | --- | --- |
| WT | Shapiro-Wilk test | 0.419497214557121 | no | ns | yes | 5 | 1.022 +/- 0.0307 |
| CM-DKO | Shapiro-Wilk test | 0.815408226547254 | no | ns | yes | 5 | 1.048 +/- 0.0441 |

## figure 6

Statistical test for fig6_a

| Testing.Category | Group.tested | Method | p | Significant | p. | Sample.size |
| --- | --- | --- | --- | --- | --- | --- |
| Group comparison | Group test | ANOVA | 3.18526488446979e-05 | yes | **** | 4 vs 4 vs 4 vs 4 |
| Pairwise comparison | shScr vs shAGER | TukeyHSD | 0.00430406365387692 | yes | ** | 4 vs 4 |
| Pairwise comparison | shScr vs shScr H/R | TukeyHSD | 0.0313119264393446 | yes | * | 4 vs 4 |
| Pairwise comparison | shScr vs shAGER H/R | TukeyHSD | 0.0597287933039194 | no | ns | 4 vs 4 |
| Pairwise comparison | shAGER vs shScr H/R | TukeyHSD | 3.19705247234792e-05 | yes | **** | 4 vs 4 |
| Pairwise comparison | shAGER vs shAGER H/R | TukeyHSD | 0.460174522502916 | no | ns | 4 vs 4 |
| Pairwise comparison | shScr H/R vs shAGER H/R | TukeyHSD | 0.00026819120810706 | yes | *** | 4 vs 4 |

Normality test for fig6_a

| Group | Method | p | Significant | p. | Whether.pass | Sample.size | SEM |
| --- | --- | --- | --- | --- | --- | --- | --- |
| shScr | Shapiro-Wilk test | 0.536134447613663 | no | ns | yes | 4 | 22.3775 +/- 3.8054 |
| shAGER | Shapiro-Wilk test | 0.261714807775604 | no | ns | yes | 4 | 3.4 +/- 1.0155 |
| shScr H/R | Shapiro-Wilk test | 0.979812915069345 | no | ns | yes | 4 | 36.4125 +/- 3.8546 |
| shAGER H/R | Shapiro-Wilk test | 0.297010825796836 | no | ns | yes | 4 | 9.9575 +/- 2.6835 |

Statistical test for fig6_b Distance between Mt-ER

| Testing.Category | Group.tested | Method | p | Significant | p. | Sample.size |
| --- | --- | --- | --- | --- | --- | --- |
| Group comparison | Group test | Kruskal-Wallis test | 1.63285285494219e-33 | yes | **** | 155 vs 155 vs 153 vs 155 |
| Pairwise comparison | shScr vs shAGER | Dunn’s Test | 1.94250634058098e-11 | yes | **** | 155 vs 155 |
| Pairwise comparison | shScr vs shScr HR | Dunn’s Test | 0.00103959915752482 | yes | ** | 155 vs 153 |
| Pairwise comparison | shScr vs shAGER H/R | Dunn’s Test | 8.0013847247929e-12 | yes | **** | 155 vs 155 |
| Pairwise comparison | shAGER vs shScr HR | Dunn’s Test | 2.07641787994071e-23 | yes | **** | 155 vs 153 |
| Pairwise comparison | shAGER vs shAGER H/R | Dunn’s Test | 0.866613622547747 | no | ns | 155 vs 155 |
| Pairwise comparison | shScr HR vs shAGER H/R | Dunn’s Test | 7.455885732839e-24 | yes | **** | 153 vs 155 |

Normality test for fig6_b Distance between Mt-ER

| Group | Method | p | Significant | p. | Whether.pass | Sample.size | SEM |
| --- | --- | --- | --- | --- | --- | --- | --- |
| shScr | Shapiro-Wilk test | 5.63265375230616e-13 | yes | **** | no | 155 | 16.2695 +/- 0.7311 |
| shAGER | Shapiro-Wilk test | 3.59429861164886e-05 | yes | **** | no | 155 | 24.7172 +/- 0.8997 |
| shScr HR | Shapiro-Wilk test | 1.83474478776864e-10 | yes | **** | no | 153 | 12.8528 +/- 0.5806 |
| shAGER H/R | Shapiro-Wilk test | 3.65072112215843e-06 | yes | **** | no | 155 | 28.1193 +/- 1.3309 |

Statistical test for fig6_c

| Groups | Statistic.Test | p | Significant | p. | Sample.Size |
| --- | --- | --- | --- | --- | --- |
| Veh H/R vs RAGE229 H/R | unpaired t-test | 0.0159153298329013 | yes | * | 4 vs 4 |

Normality test for fig6_c

| Group | Normality.Test | p | Significant | p. | Pass.normality.test. | Sample.Size | SEM |
| --- | --- | --- | --- | --- | --- | --- | --- |
| Veh H/R | Shapiro-Wilk test | 0.284344076340454 | no | ns | yes | 4 | 18.0638 +/- 3.4662 |
| RAGE229 H/R | Shapiro-Wilk test | 0.056955626646703 | no | ns | yes | 4 | 1.0119 +/- 0.2002 |

Statistical test for fig6_d

| Testing.Category | Group.tested | Method | p | Significant | p. | Sample.size |
| --- | --- | --- | --- | --- | --- | --- |
| Group comparison | Group test | Welch’s ANOVA | 0.000108575873482293 | yes | *** | 6 vs 6 vs 6 vs 6 |
| Pairwise comparison | Veh vs CML | Games-Howell Test | 0.015 | yes | * | 6 vs 6 |
| Pairwise comparison | Veh vs CML + RAGE229 | Games-Howell Test | 0.02 | yes | * | 6 vs 6 |
| Pairwise comparison | Veh vs RAGE229 | Games-Howell Test | 0.023 | yes | * | 6 vs 6 |
| Pairwise comparison | CML vs CML + RAGE229 | Games-Howell Test | 0.002 | yes | ** | 6 vs 6 |
| Pairwise comparison | CML vs RAGE229 | Games-Howell Test | 0.002 | yes | ** | 6 vs 6 |
| Pairwise comparison | CML + RAGE229 vs RAGE229 | Games-Howell Test | 0.955 | no | ns | 6 vs 6 |

Normality test for fig6_d

| Group | Method | p | Significant | p. | Whether.pass | Sample.size | SEM |
| --- | --- | --- | --- | --- | --- | --- | --- |
| Veh | Shapiro-Wilk test | 0.737758123053672 | no | ns | yes | 6 | 12.4278 +/- 2.1565 |
| CML | Shapiro-Wilk test | 0.251908055779583 | no | ns | yes | 6 | 29.4094 +/- 3.5864 |
| CML + RAGE229 | Shapiro-Wilk test | 0.0719673766034389 | no | ns | yes | 6 | 2.6023 +/- 0.5364 |
| RAGE229 | Shapiro-Wilk test | 0.471680506940504 | no | ns | yes | 6 | 3.0417 +/- 0.6768 |

## Fig7

Statistical test for fig7_c

| Testing.Category | Group.tested | Method | p | Significant | p. | Sample.size |
| --- | --- | --- | --- | --- | --- | --- |
| Group comparison | Group test | Kruskal-Wallis test | 2.40802632965721e-54 | yes | **** | 151 vs 151 vs 151 |
| Pairwise comparison | shScr H/R vs shDIAPH1 H/R | Dunn’s Test | 1.98226977069826e-22 | yes | **** | 151 vs 151 |
| Pairwise comparison | shScr H/R vs shDIAPH1 H/R + Linker | Dunn’s Test | 8.56216782318685e-09 | yes | **** | 151 vs 151 |
| Pairwise comparison | shDIAPH1 H/R vs shDIAPH1 H/R + Linker | Dunn’s Test | 5.51009760699674e-54 | yes | **** | 151 vs 151 |

Normality test for fig7_c

| Group | Method | p | Significant | p. | Whether.pass | Sample.size | SEM |
| --- | --- | --- | --- | --- | --- | --- | --- |
| shScr H/R | Shapiro-Wilk test | 6.81403967243737e-10 | yes | **** | no | 151 | 12.7935 +/- 0.5536 |
| shDIAPH1 H/R | Shapiro-Wilk test | 0.000693859539243282 | yes | *** | no | 151 | 27.1566 +/- 0.9355 |
| shDIAPH1 H/R + Linker | Shapiro-Wilk test | 1.14924033066326e-06 | yes | **** | no | 151 | 8.1162 +/- 0.3133 |

Statistical test for fig7_d

| Testing.Category | Group.tested | Method | p | Significant | p. | Sample.size |
| --- | --- | --- | --- | --- | --- | --- |
| Group comparison | Group test | ANOVA | 0.000169111993219871 | yes | *** | 6 vs 4 vs 6 |
| Pairwise comparison | shScr H/R vs shDIAPH1 H/R | TukeyHSD | 0.000307802779155875 | yes | *** | 6 vs 4 |
| Pairwise comparison | shScr H/R vs shDIAPH1 H/R-Linker | TukeyHSD | 0.99440029438194 | no | ns | 6 vs 6 |
| Pairwise comparison | shDIAPH1 H/R vs shDIAPH1 H/R-Linker | TukeyHSD | 0.000358789935481996 | yes | *** | 4 vs 6 |

Normality test for fig7_d

| Group | Method | p | Significant | p. | Whether.pass | Sample.size | SEM |
| --- | --- | --- | --- | --- | --- | --- | --- |
| shScr H/R | Shapiro-Wilk test | 0.552561593185714 | no | ns | yes | 6 | 0.0019 +/- 1e-04 |
| shDIAPH1 H/R | Shapiro-Wilk test | 0.132834480692908 | no | ns | yes | 4 | 0.001 +/- 1e-04 |
| shDIAPH1 H/R-Linker | Shapiro-Wilk test | 0.69910972009128 | no | ns | yes | 6 | 0.0019 +/- 1e-04 |

Statistical test for fig7_e

| Testing.Category | Group.tested | Method | p | Significant | p. | Sample.size |
| --- | --- | --- | --- | --- | --- | --- |
| Group comparison | Group test | ANOVA | 6.30944235104593e-05 | yes | **** | 6 vs 6 vs 6 |
| Pairwise comparison | shScr vs shDIAPH1 | TukeyHSD | 4.34361695861307e-05 | yes | **** | 6 vs 6 |
| Pairwise comparison | shScr vs shDIAPH1+Linker | TukeyHSD | 0.0481990862723729 | yes | * | 6 vs 6 |
| Pairwise comparison | shDIAPH1 vs shDIAPH1+Linker | TukeyHSD | 0.00646499976921056 | yes | ** | 6 vs 6 |

Normality test for fig7_e

| Group | Method | p | Significant | p. | Whether.pass | Sample.size | SEM |
| --- | --- | --- | --- | --- | --- | --- | --- |
| shScr | Shapiro-Wilk test | 0.885855865792731 | no | ns | yes | 6 | 4.1147 +/- 0.1247 |
| shDIAPH1 | Shapiro-Wilk test | 0.101734292175311 | no | ns | yes | 6 | 2.1425 +/- 0.1403 |
| shDIAPH1+Linker | Shapiro-Wilk test | 0.140207408483529 | no | ns | yes | 6 | 3.2895 +/- 0.3375 |

Statistical test for fig7_d

| Testing.Category | Group.tested | Method | p | Significant | p. | Sample.size |
| --- | --- | --- | --- | --- | --- | --- |
| Group comparison | Group test | ANOVA | 0.000169111993219871 | yes | *** | 6 vs 4 vs 6 |
| Pairwise comparison | shScr H/R vs shDIAPH1 H/R | TukeyHSD | 0.000307802779155875 | yes | *** | 6 vs 4 |
| Pairwise comparison | shScr H/R vs shDIAPH1 H/R-Linker | TukeyHSD | 0.99440029438194 | no | ns | 6 vs 6 |
| Pairwise comparison | shDIAPH1 H/R vs shDIAPH1 H/R-Linker | TukeyHSD | 0.000358789935481996 | yes | *** | 4 vs 6 |

Normality test for fig7_d

| Group | Method | p | Significant | p. | Whether.pass | Sample.size | SEM |
| --- | --- | --- | --- | --- | --- | --- | --- |
| shScr H/R | Shapiro-Wilk test | 0.552561593185714 | no | ns | yes | 6 | 0.0019 +/- 1e-04 |
| shDIAPH1 H/R | Shapiro-Wilk test | 0.132834480692908 | no | ns | yes | 4 | 0.001 +/- 1e-04 |
| shDIAPH1 H/R-Linker | Shapiro-Wilk test | 0.69910972009128 | no | ns | yes | 6 | 0.0019 +/- 1e-04 |

Statistical test for fig7_f_DIAPH1

| Testing.Category | Group.tested | Method | p | Significant | p. | Sample.size |
| --- | --- | --- | --- | --- | --- | --- |
| Group comparison | Group test | Welch’s ANOVA | 0.0021413911568074 | yes | ** | 4 vs 4 vs 4 |
| Pairwise comparison | shScr H/R vs shDIAPH1 H/R | Games-Howell Test | 0.007 | yes | *** | 4 vs 4 |
| Pairwise comparison | shScr H/R vs shDIAPH1 H/R + Linker | Games-Howell Test | 0.006 | yes | *** | 4 vs 4 |
| Pairwise comparison | shDIAPH1 H/R vs shDIAPH1 H/R + Linker | Games-Howell Test | 0.95 | no | ns | 4 vs 4 |

Normality test for fig7_f_DIAPH1

| Group | Method | p | Significant | p. | Whether.pass | Sample.size | SEM |
| --- | --- | --- | --- | --- | --- | --- | --- |
| shScr H/R | Shapiro-Wilk test | 0.149806481430881 | no | ns | yes | 4 | 1.0166 +/- 0.1056 |
| shDIAPH1 H/R | Shapiro-Wilk test | 0.800237286315775 | no | ns | yes | 4 | 0.1654 +/- 0.0156 |
| shDIAPH1 H/R + Linker | Shapiro-Wilk test | 0.617049271953607 | no | ns | yes | 4 | 0.1758 +/- 0.03 |

Statistical test for fig7_f_BCL2

| Testing.Category | Group.tested | Method | p | Significant | p. | Sample.size |
| --- | --- | --- | --- | --- | --- | --- |
| Group comparison | Group test | ANOVA | 8.45112266878164e-05 | yes | **** | 4 vs 4 vs 4 |
| Pairwise comparison | shScr H/R vs shDIAPH1 H/R | TukeyHSD | 0.000103660454229826 | yes | *** | 4 vs 4 |
| Pairwise comparison | shScr H/R vs shDIAPH1 H/R + Linker | TukeyHSD | 0.436331521805354 | no | ns | 4 vs 4 |
| Pairwise comparison | shDIAPH1 H/R vs shDIAPH1 H/R + Linker | TukeyHSD | 0.000438805817435495 | yes | *** | 4 vs 4 |

Normality test for fig7_f_BCL2

| Group | Method | p | Significant | p. | Whether.pass | Sample.size | SEM |
| --- | --- | --- | --- | --- | --- | --- | --- |
| shScr H/R | Shapiro-Wilk test | 0.0590992828363695 | no | ns | yes | 4 | 1.0092 +/- 0.0783 |
| shDIAPH1 H/R | Shapiro-Wilk test | 0.662869263837824 | no | ns | yes | 4 | 1.7964 +/- 0.0815 |
| shDIAPH1 H/R + Linker | Shapiro-Wilk test | 0.37586164189862 | no | ns | yes | 4 | 1.1453 +/- 0.0631 |

Statistical test for fig7_f_PARKIN

| Testing.Category | Group.tested | Method | p | Significant | p. | Sample.size |
| --- | --- | --- | --- | --- | --- | --- |
| Group comparison | Group test | ANOVA | 0.00012633943832722 | yes | *** | 4 vs 4 vs 4 |
| Pairwise comparison | shScr H/R vs shDIAPH1 H/R | TukeyHSD | 0.000179862055751046 | yes | *** | 4 vs 4 |
| Pairwise comparison | shScr H/R vs shDIAPH1 H/R + Linker | TukeyHSD | 0.679129557931956 | no | ns | 4 vs 4 |
| Pairwise comparison | shDIAPH1 H/R vs shDIAPH1 H/R + Linker | TukeyHSD | 0.000483739247015946 | yes | *** | 4 vs 4 |

Normality test for fig7_f_PARKIN

| Group | Method | p | Significant | p. | Whether.pass | Sample.size | SEM |
| --- | --- | --- | --- | --- | --- | --- | --- |
| shScr H/R | Shapiro-Wilk test | 0.195568008468133 | no | ns | yes | 4 | 1.0036 +/- 0.0491 |
| shDIAPH1 H/R | Shapiro-Wilk test | 0.716084822124027 | no | ns | yes | 4 | 2.0327 +/- 0.1285 |
| shDIAPH1 H/R + Linker | Shapiro-Wilk test | 0.391843283724592 | no | ns | yes | 4 | 1.1308 +/- 0.1188 |

Statistical test for fig7_f_PERK

| Testing.Category | Group.tested | Method | p | Significant | p. | Sample.size |
| --- | --- | --- | --- | --- | --- | --- |
| Group comparison | Group test | Welch’s ANOVA | 0.00402536972336425 | yes | ** | 4 vs 4 vs 4 |
| Pairwise comparison | shScr H/R vs shDIAPH1 H/R | Games-Howell Test | 0.008 | yes | ** | 4 vs 4 |
| Pairwise comparison | shScr H/R vs shDIAPH1 H/R + Linker | Games-Howell Test | 0.207 | no | ns | 4 vs 4 |
| Pairwise comparison | shDIAPH1 H/R vs shDIAPH1 H/R + Linker | Games-Howell Test | 0.012 | yes | * | 4 vs 4 |

Normality test for fig7_f_PERK

| Group | Method | p | Significant | p. | Whether.pass | Sample.size | SEM |
| --- | --- | --- | --- | --- | --- | --- | --- |
| shScr H/R | Shapiro-Wilk test | 0.36928723982664 | no | ns | yes | 4 | 1 +/- 0.0047 |
| shDIAPH1 H/R | Shapiro-Wilk test | 0.546080381765419 | no | ns | yes | 4 | 0.5469 +/- 0.0573 |
| shDIAPH1 H/R + Linker | Shapiro-Wilk test | 0.288621899761831 | no | ns | yes | 4 | 0.8813 +/- 0.0527 |

Statistical test for fig7_f_GADD34

| Testing.Category | Group.tested | Method | p | Significant | p. | Sample.size |
| --- | --- | --- | --- | --- | --- | --- |
| Group comparison | Group test | Kruskal-Wallis test | 0.00727670649933249 | yes | ** | 4 vs 4 vs 4 |
| Pairwise comparison | shScr H/R vs shDIAPH1 H/R | Dunn’s Test | 0.00510561627801905 | yes | ** | 4 vs 4 |
| Pairwise comparison | shScr H/R vs shDIAPH1H/R + Linker | Dunn’s Test | 0.116664464781023 | no | ns | 4 vs 4 |
| Pairwise comparison | shDIAPH1 H/R vs shDIAPH1H/R + Linker | Dunn’s Test | 0.116664464781023 | no | ns | 4 vs 4 |

Normality test for fig7_f_GADD34

| Group | Method | p | Significant | p. | Whether.pass | Sample.size | SEM |
| --- | --- | --- | --- | --- | --- | --- | --- |
| shScr H/R | Shapiro-Wilk test | 0.711196405088101 | no | ns | yes | 4 | 1.0173 +/- 0.1087 |
| shDIAPH1 H/R | Shapiro-Wilk test | 0.00486038174338182 | yes | ** | no | 4 | 0.1421 +/- 0.0162 |
| shDIAPH1H/R + Linker | Shapiro-Wilk test | 0.0208330286664289 | yes | * | no | 4 | 0.5043 +/- 0.0261 |

Statistical test for fig7_f_EDEM1

| Testing.Category | Group.tested | Method | p | Significant | p. | Sample.size |
| --- | --- | --- | --- | --- | --- | --- |
| Group comparison | Group test | Welch’s ANOVA | 9.2551698653432e-05 | yes | **** | 4 vs 4 vs 4 |
| Pairwise comparison | shScr H/R vs shDIAPH1 H/R | Games-Howell Test | 0.000444 | yes | *** | 4 vs 4 |
| Pairwise comparison | shScr H/R vs shDIAPH1H/R + Linker | Games-Howell Test | 0.000696 | yes | *** | 4 vs 4 |
| Pairwise comparison | shDIAPH1 H/R vs shDIAPH1H/R + Linker | Games-Howell Test | 0.027 | yes | * | 4 vs 4 |

Normality test for fig7_f_EDEM1

| Group | Method | p | Significant | p. | Whether.pass | Sample.size | SEM |
| --- | --- | --- | --- | --- | --- | --- | --- |
| shScr H/R | Shapiro-Wilk test | 0.993823145094351 | no | ns | yes | 4 | 1.0024 +/- 0.0402 |
| shDIAPH1 H/R | Shapiro-Wilk test | 0.661253419700051 | no | ns | yes | 4 | 0.2228 +/- 0.0083 |
| shDIAPH1H/R + Linker | Shapiro-Wilk test | 0.439856975566333 | no | ns | yes | 4 | 0.4868 +/- 0.0514 |
