# Supplementary material for: DIAPH1-MFN2 interaction regulates mitochondria-SR/ER contact and modulates ischemic/hypoxic stress
Source: Nat Commun. 2023 Oct 30;14:6900. doi: 10.1038/s41467-023-42521-x (PMC10616211; doi:10.1038/s41467-023-42521-x)
Supplement: Supplementary file 6 — Source data [file 41467_2023_42521_MOESM6_ESM.zip › Source Data/Western Blotting_uncropped blots.pptx]

## Slide 1
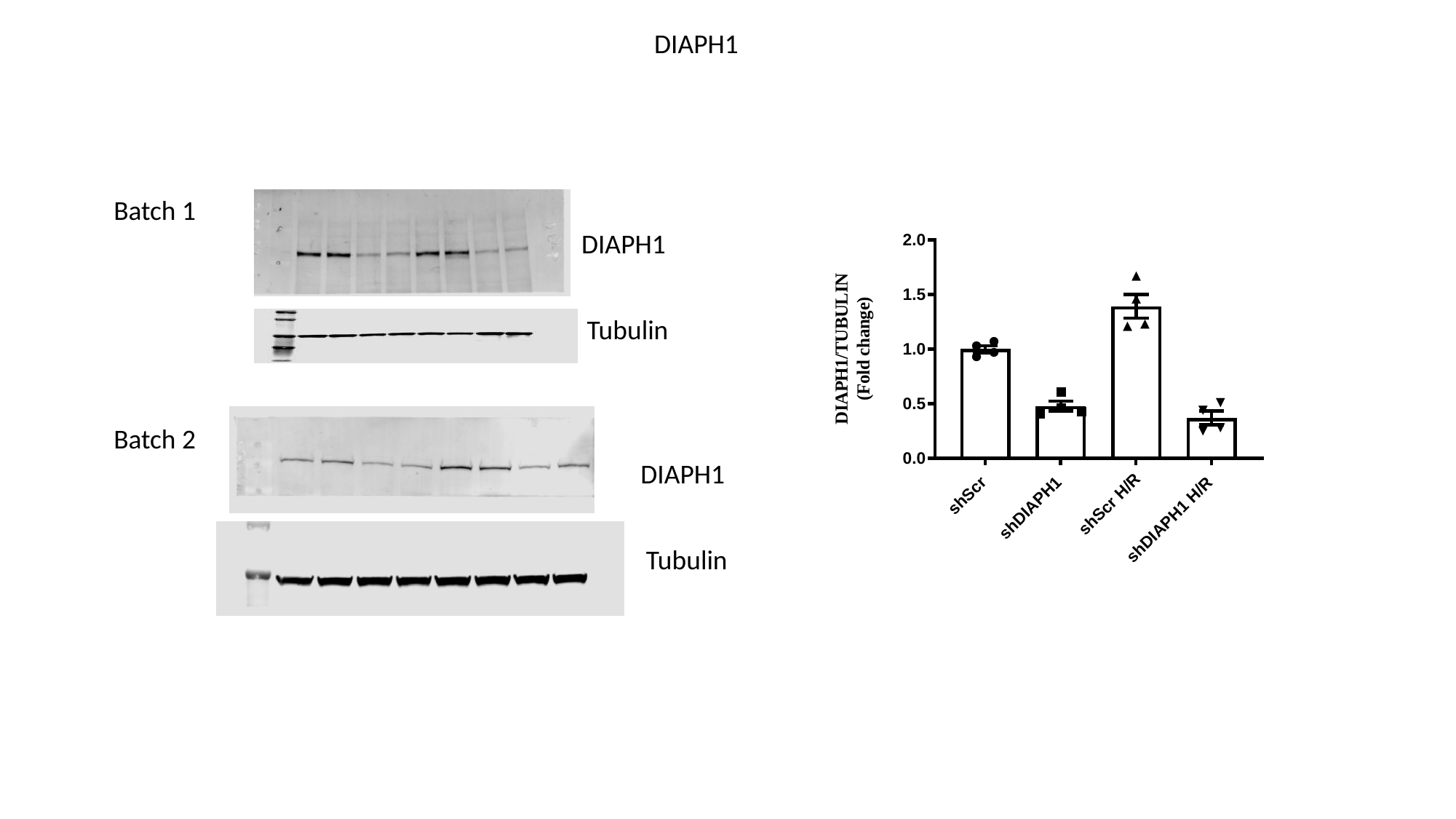

DIAPH1
Batch 1
DIAPH1
Tubulin
Batch 2
DIAPH1
Tubulin

## Slide 2
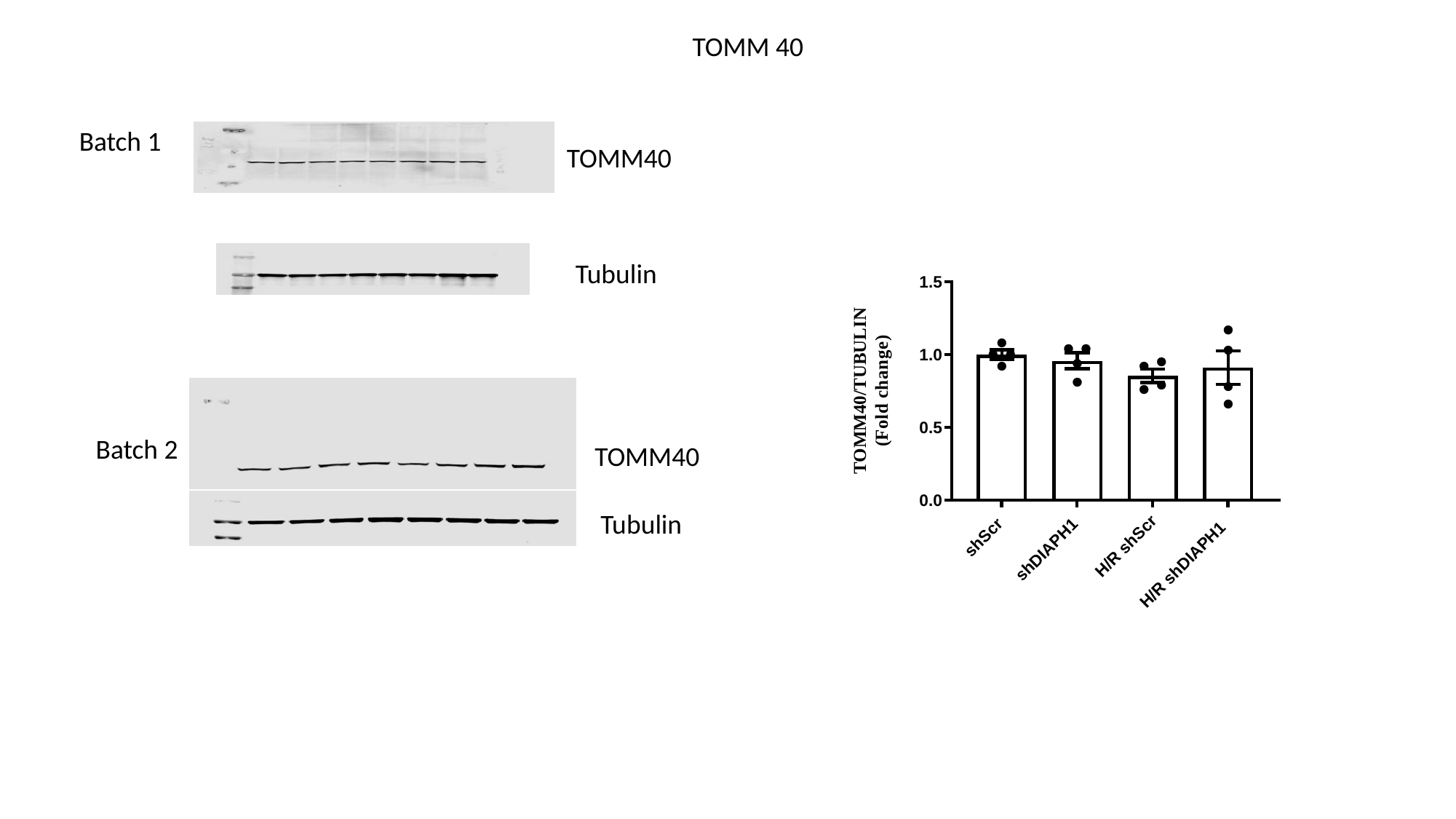

TOMM 40
Batch 1
TOMM40
Tubulin
Batch 2
TOMM40
Tubulin

## Slide 3
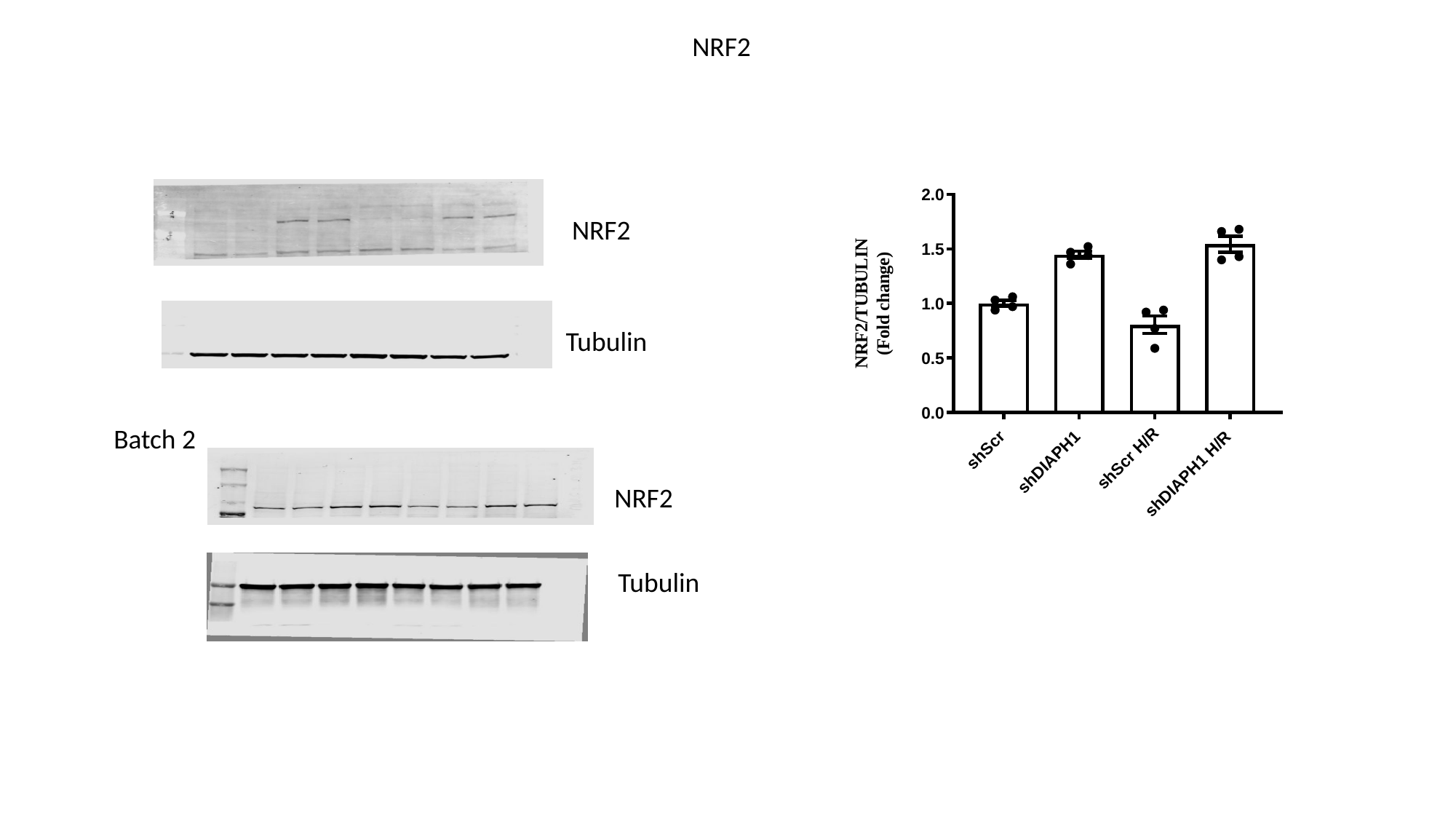

NRF2
NRF2
Tubulin
Batch 2
NRF2
Tubulin

## Slide 4
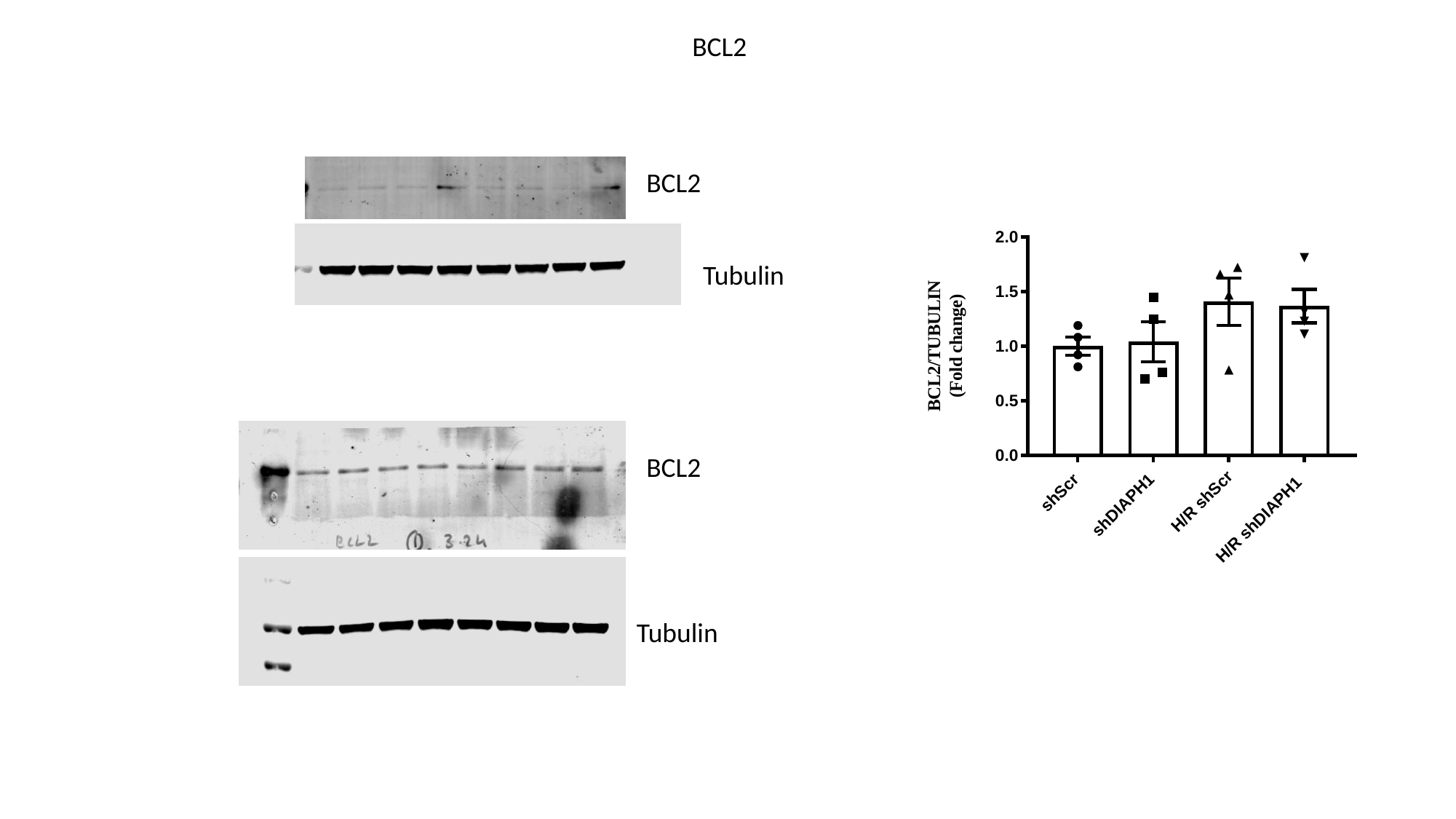

BCL2
BCL2
Tubulin
BCL2
Tubulin

## Slide 5
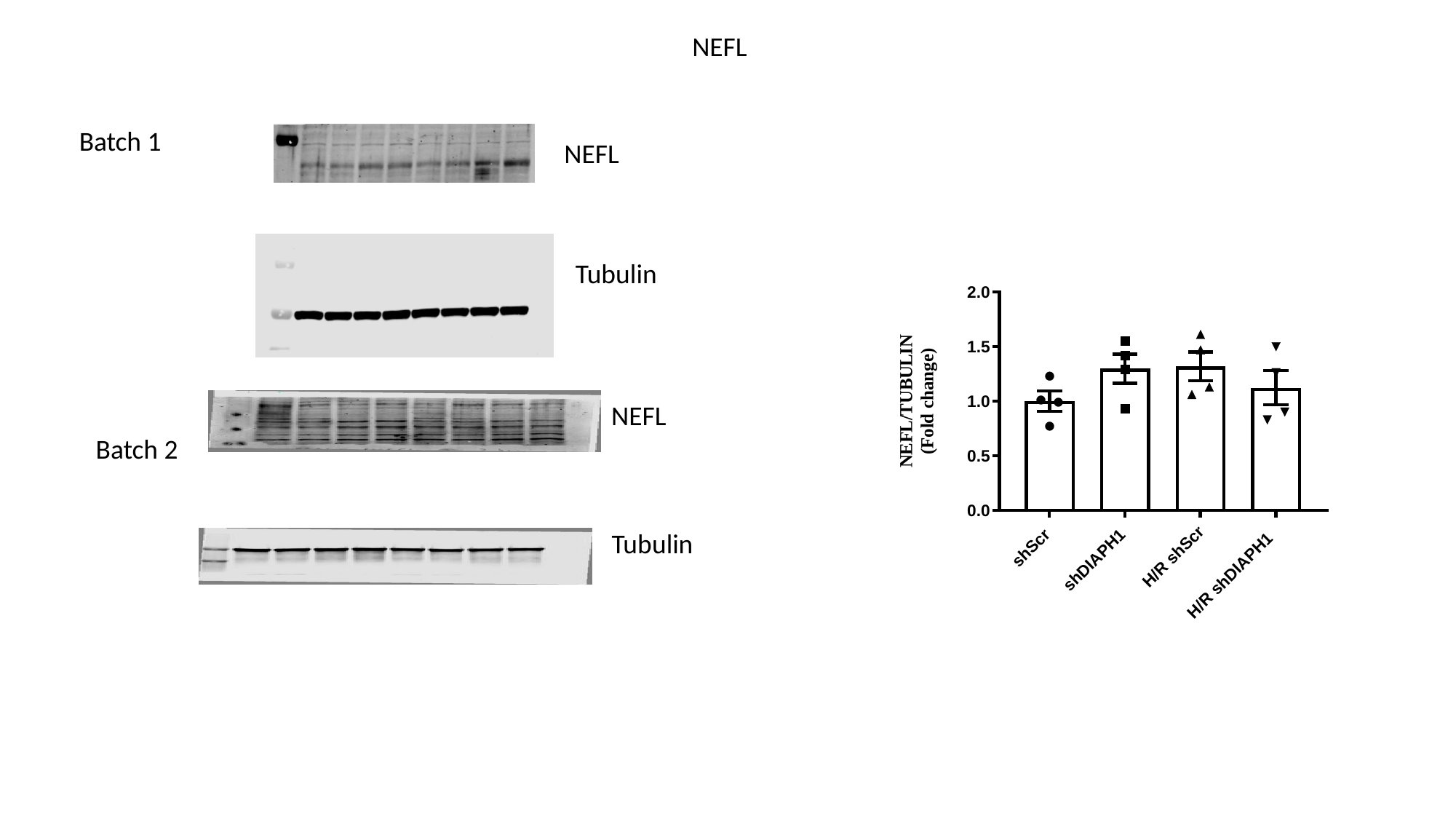

NEFL
Batch 1
NEFL
Tubulin
NEFL
Batch 2
Tubulin

## Slide 6
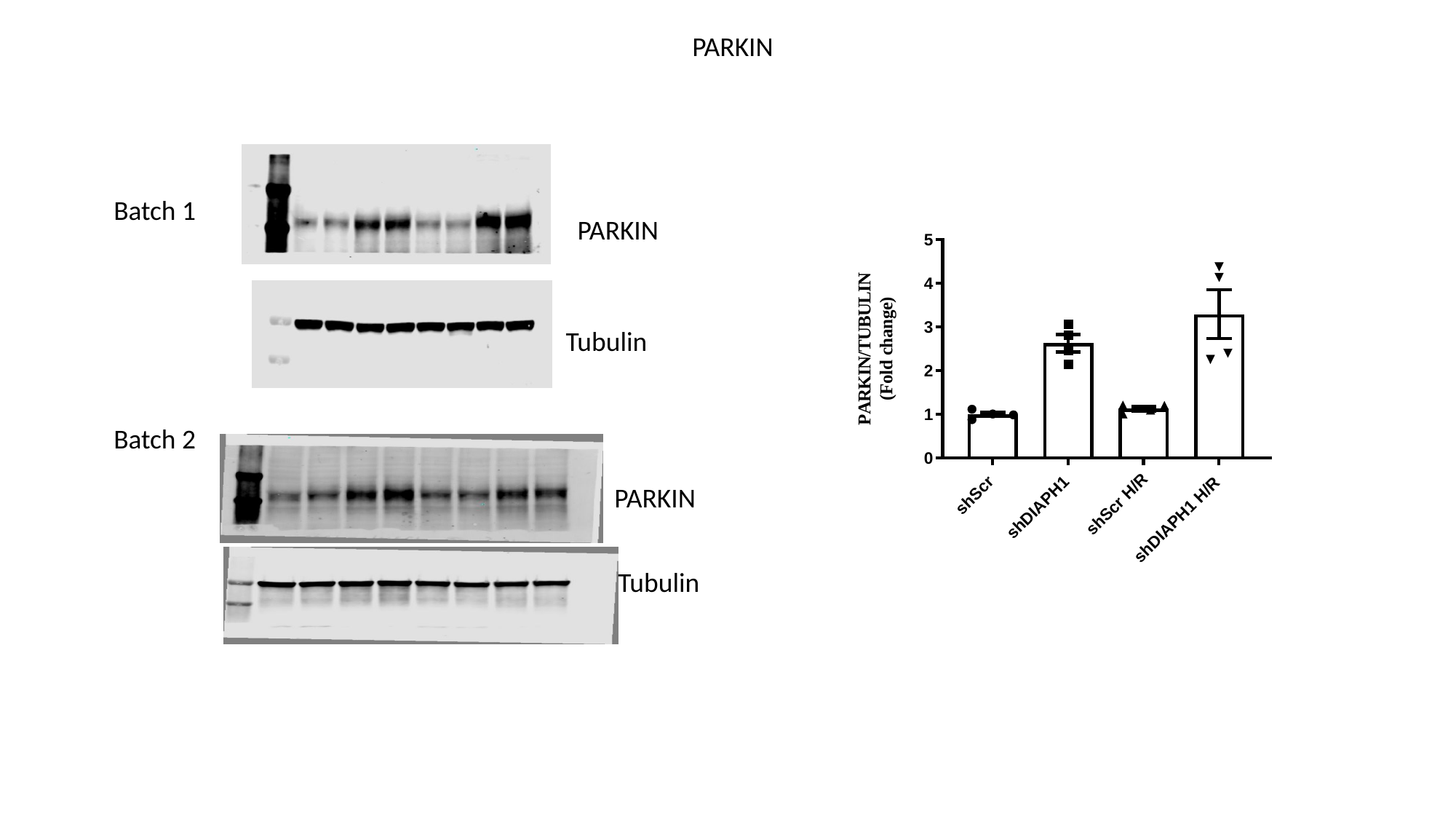

PARKIN
Batch 1
PARKIN
Tubulin
Batch 2
PARKIN
Tubulin

## Slide 7
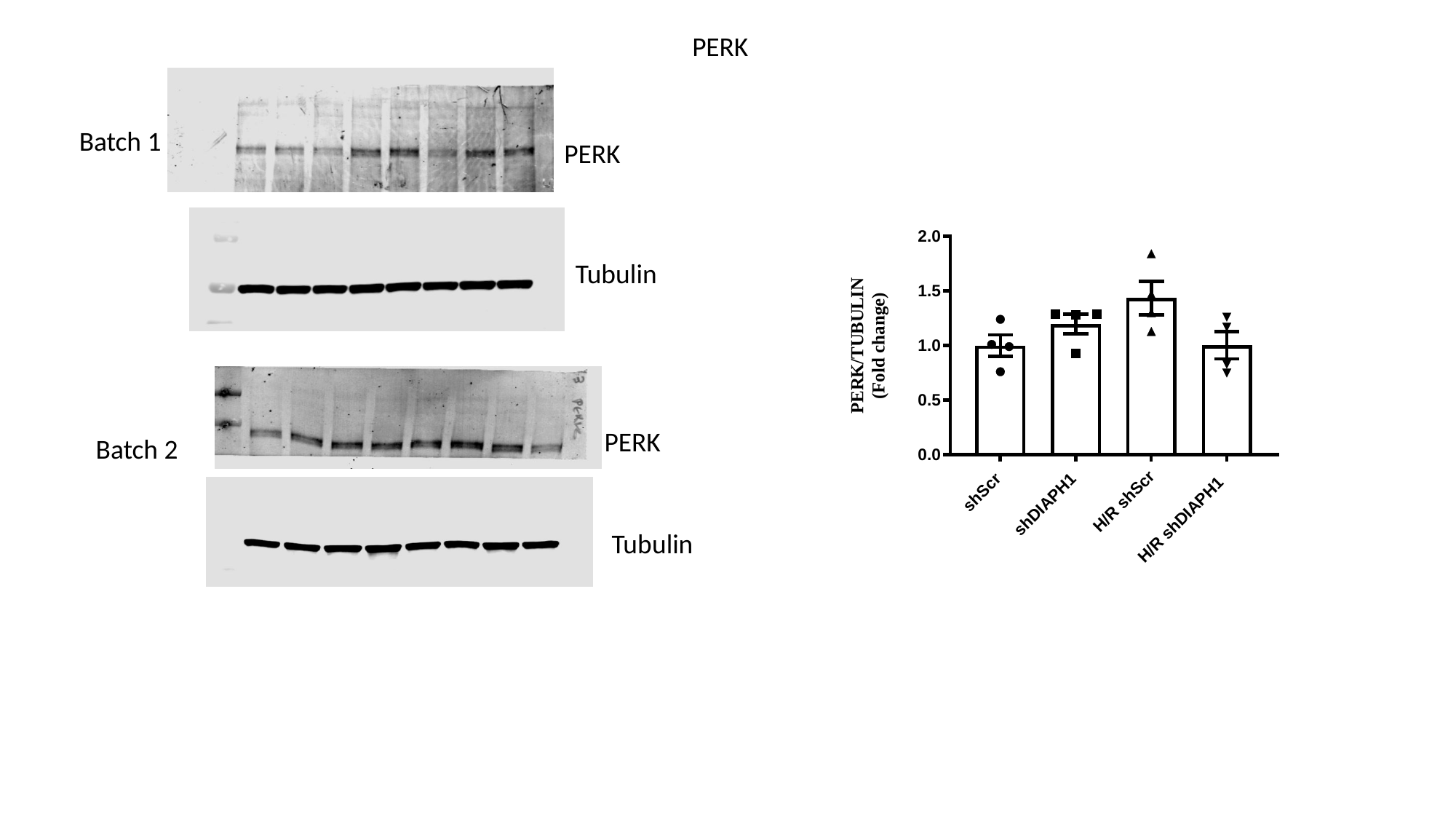

PERK
Batch 1
PERK
Tubulin
PERK
Batch 2
Tubulin

## Slide 8
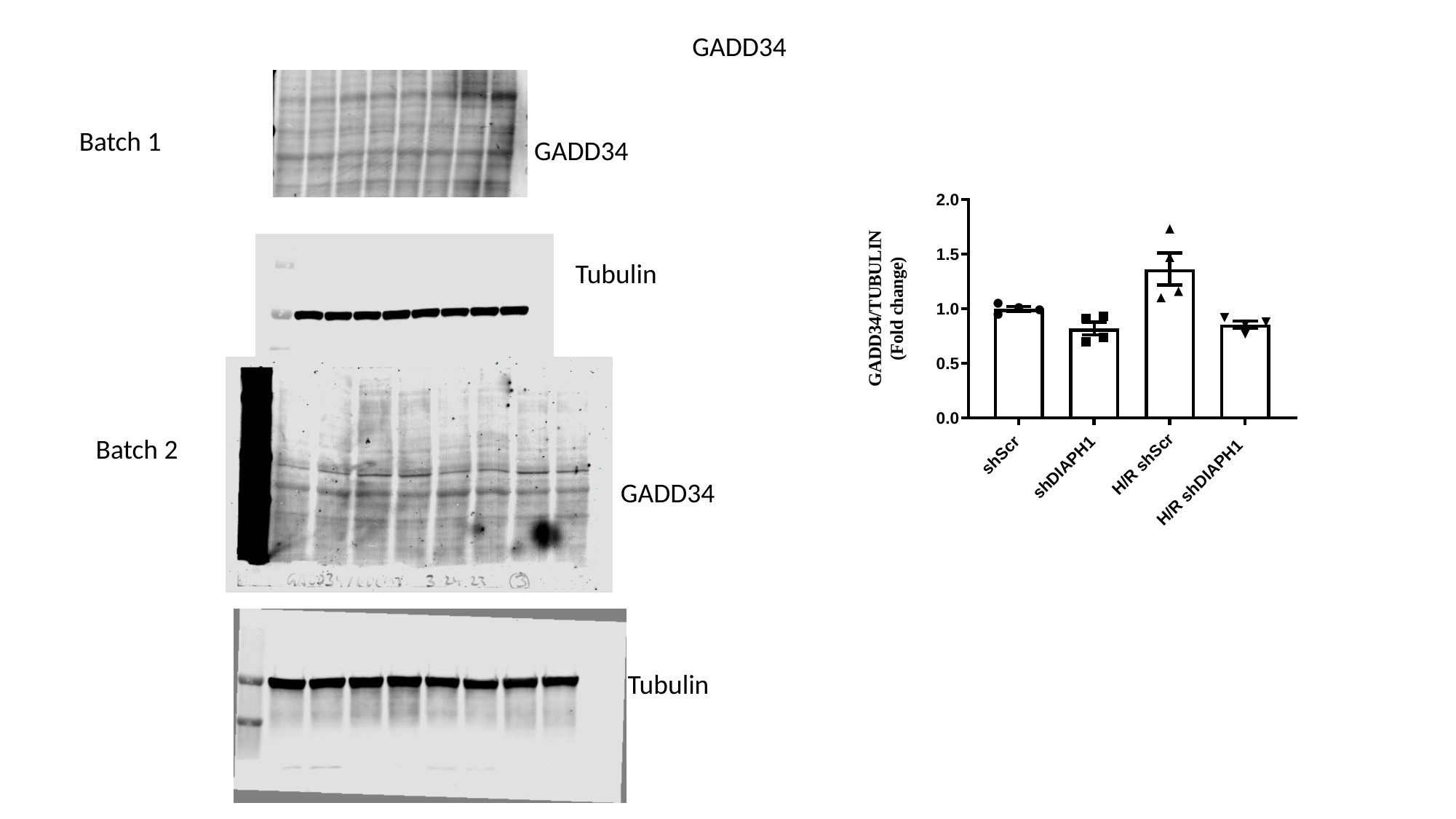

GADD34
Batch 1
GADD34
Tubulin
Batch 2
GADD34
Tubulin

## Slide 9
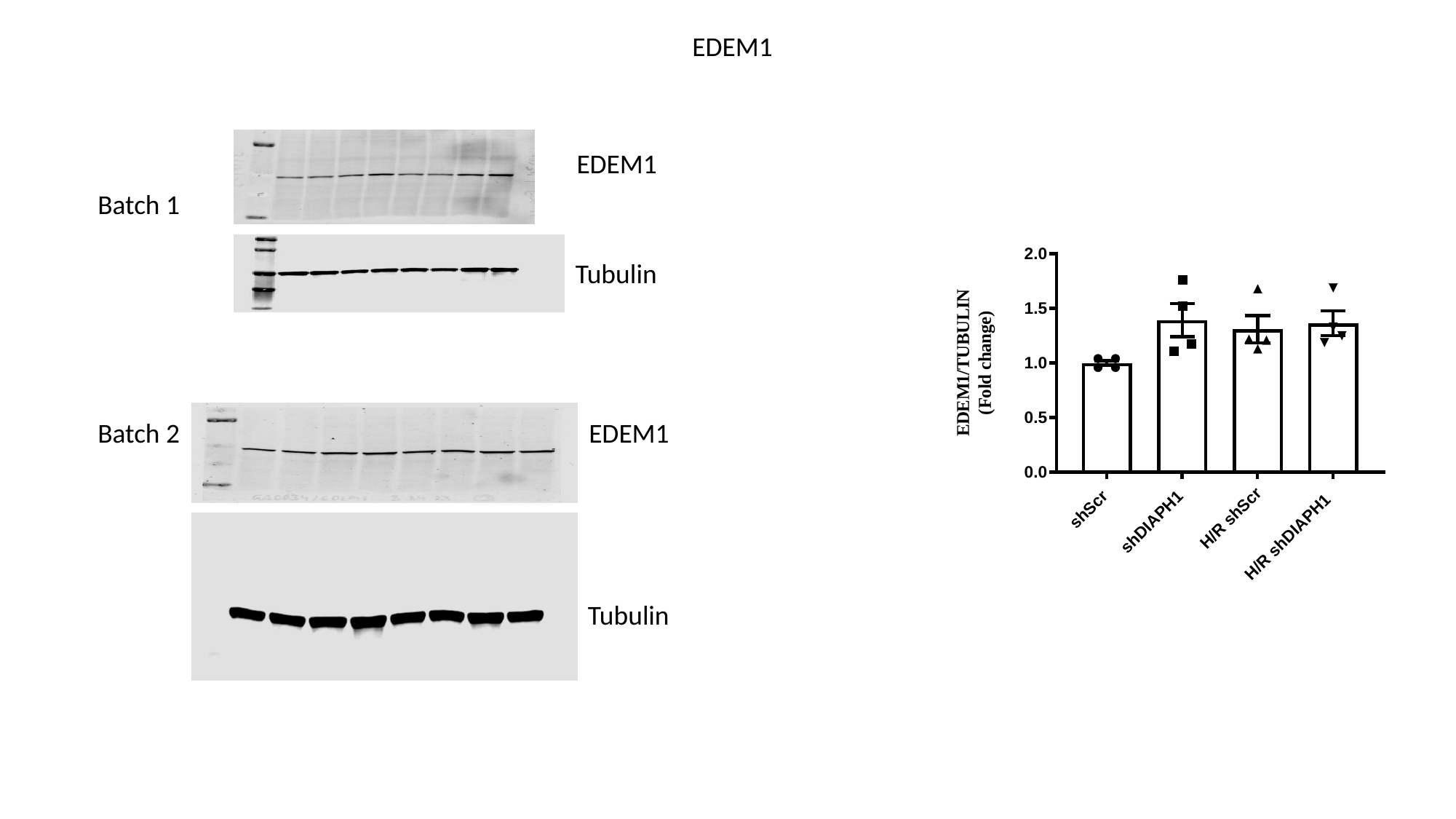

EDEM1
EDEM1
Batch 1
Tubulin
Batch 2
EDEM1
Tubulin

## Slide 10
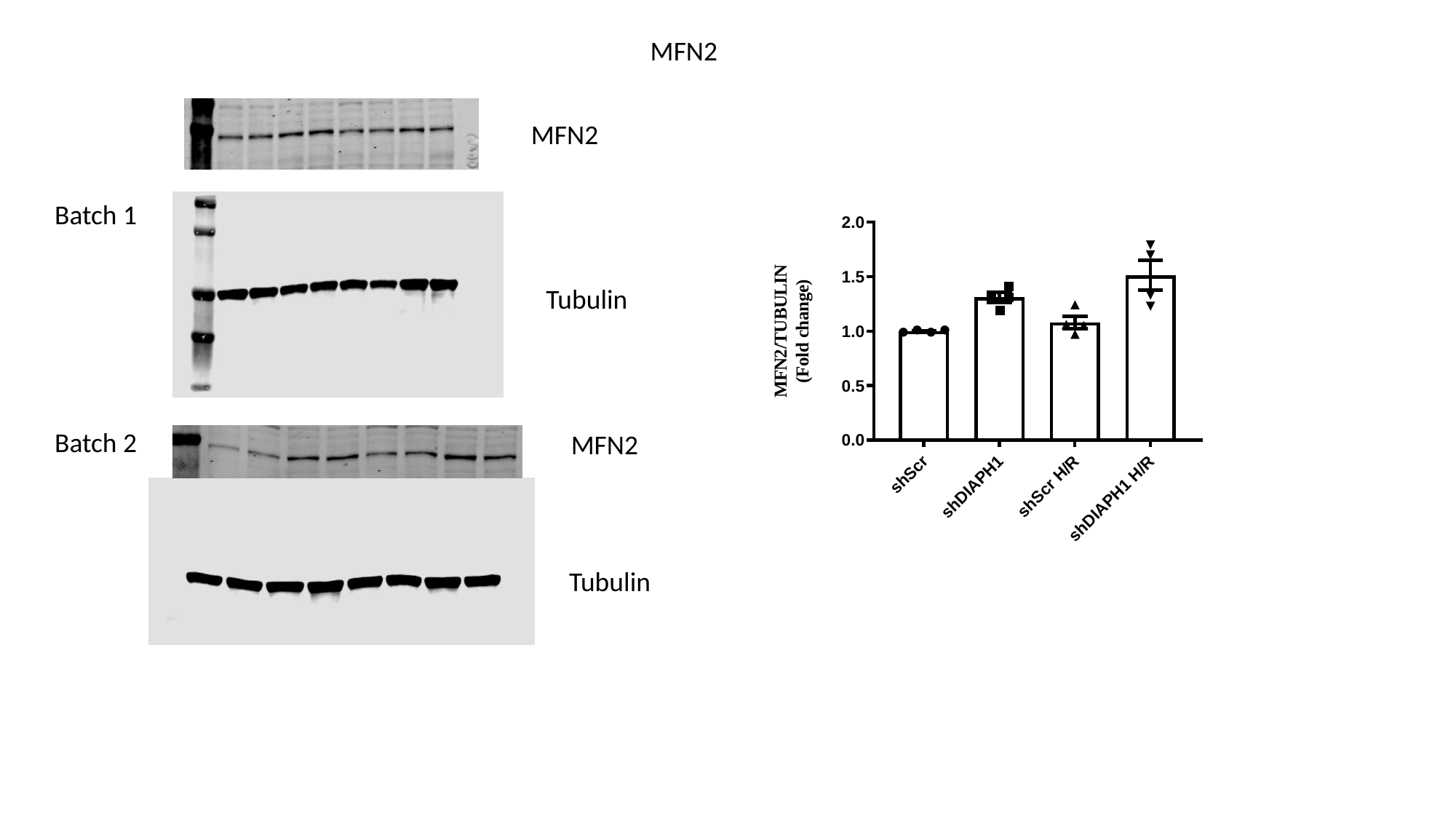

MFN2
MFN2
Batch 1
Tubulin
Batch 2
MFN2
Tubulin

## Slide 11
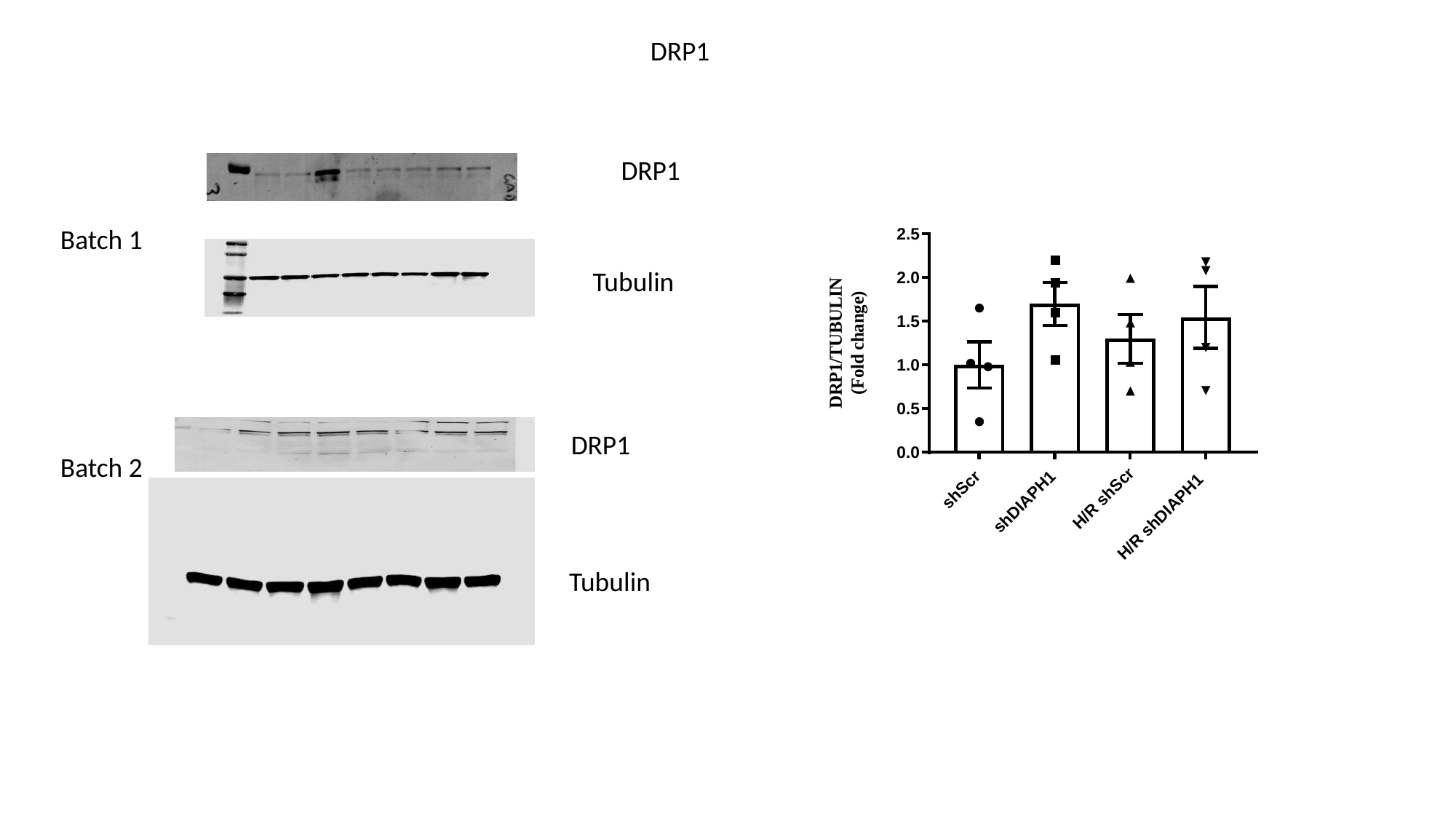

DRP1
DRP1
Batch 1
Tubulin
DRP1
Batch 2
Tubulin

## Slide 12
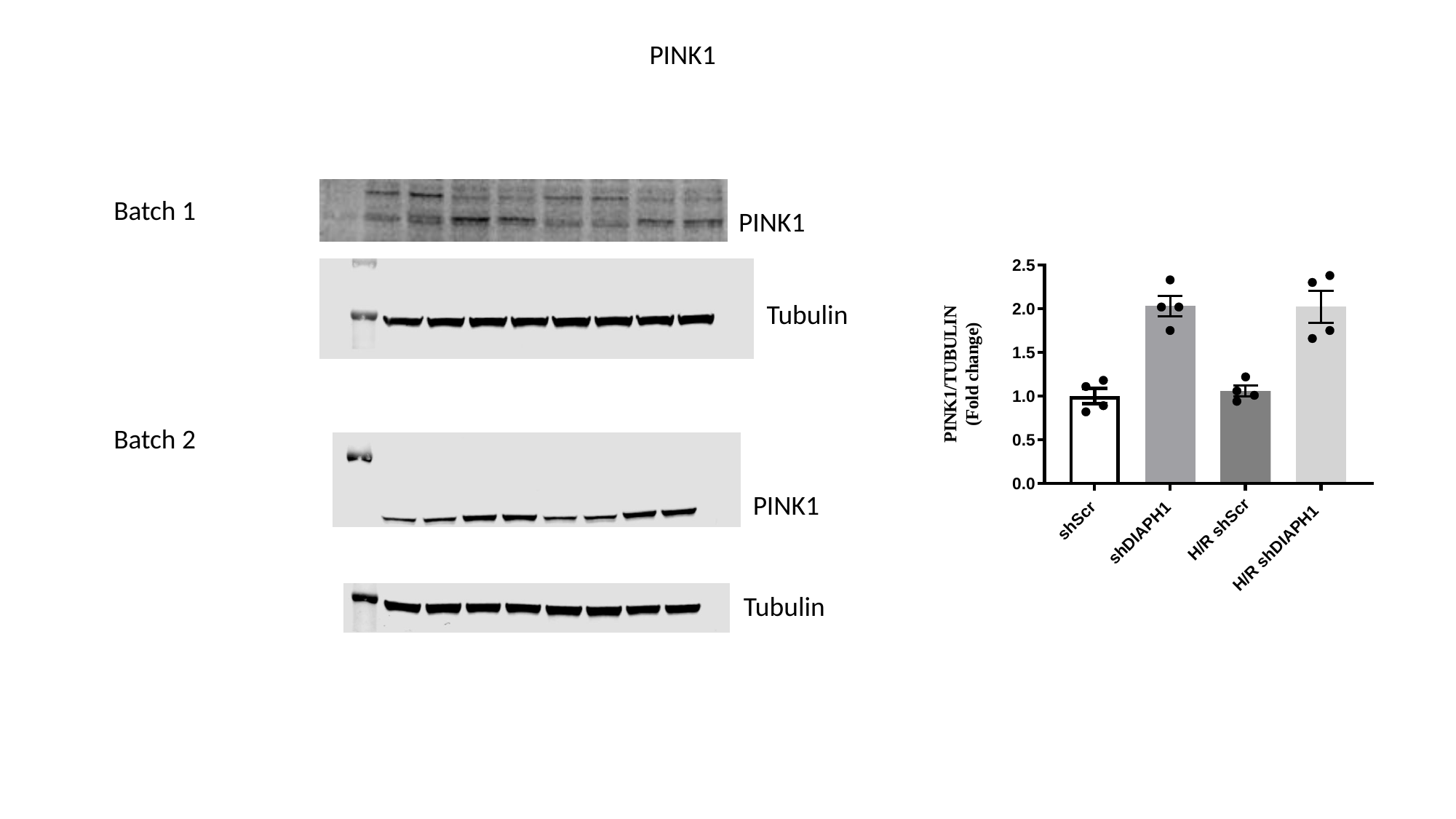

PINK1
Batch 1
PINK1
Tubulin
Batch 2
PINK1
Tubulin

## Slide 13
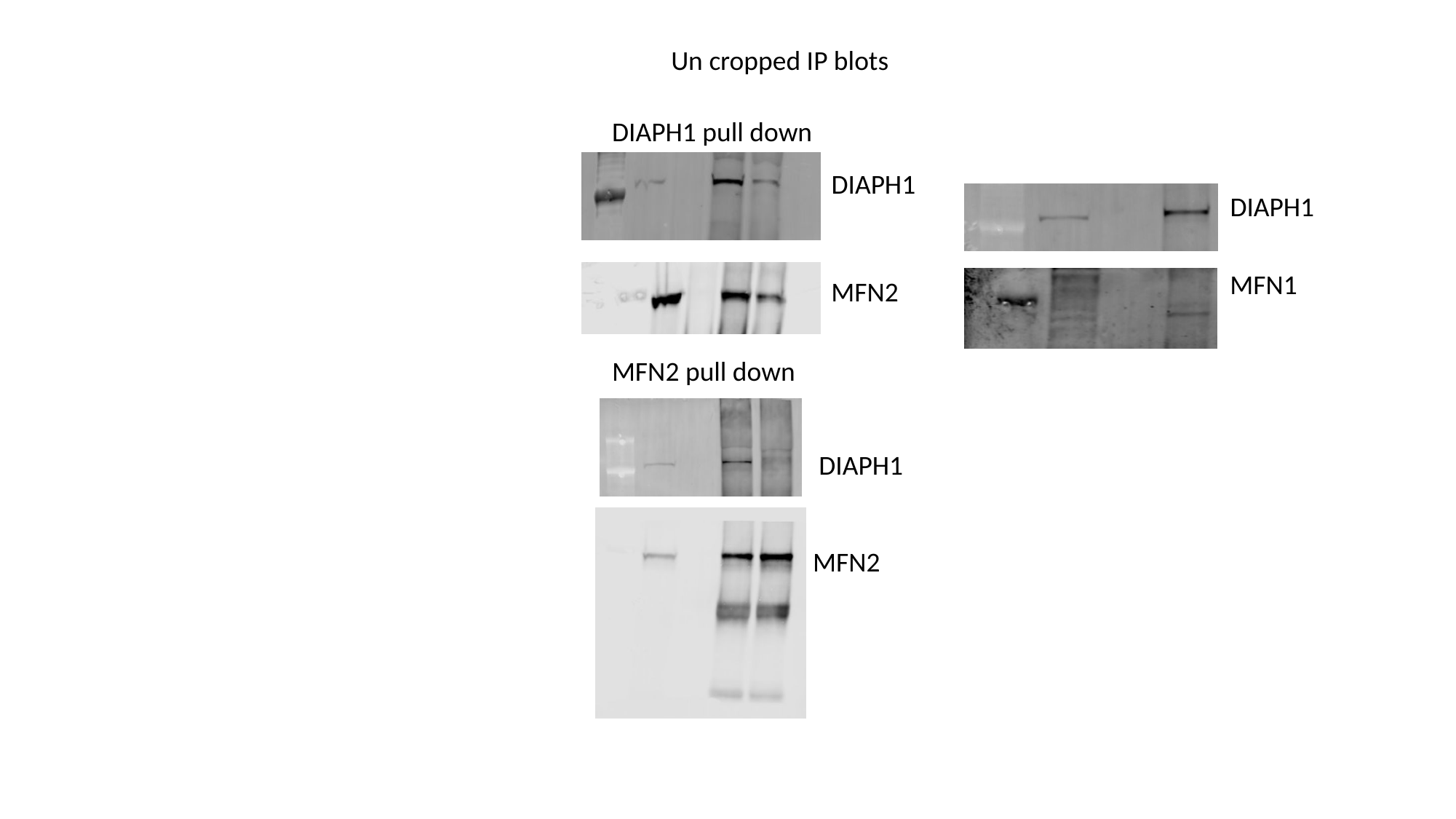

Un cropped IP blots
DIAPH1 pull down
DIAPH1
DIAPH1
MFN1
MFN2
MFN2 pull down
DIAPH1
MFN2

## Slide 14
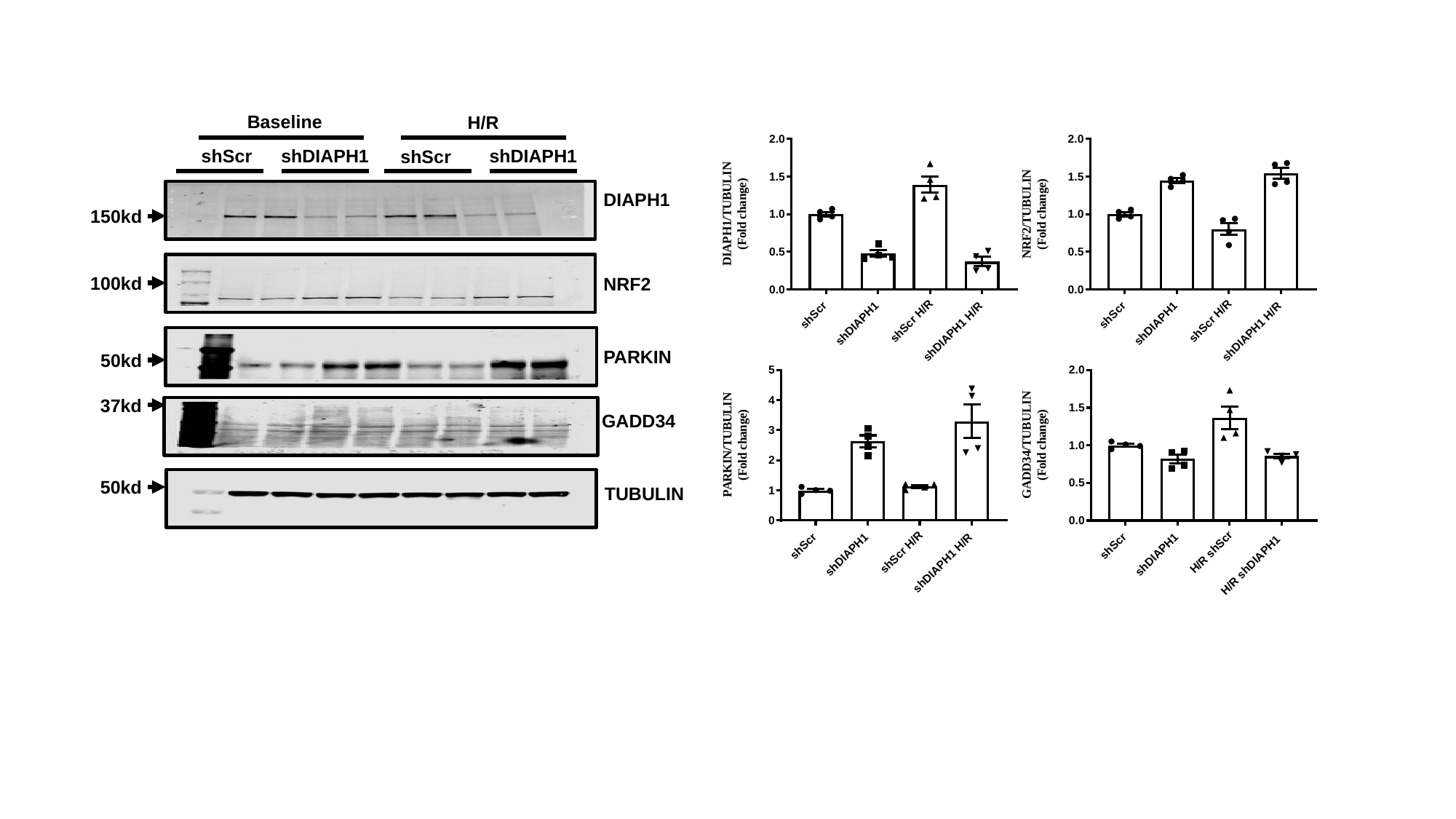

Baseline
H/R
shScr
shDIAPH1
shDIAPH1
shScr
DIAPH1
150kd
100kd
NRF2
PARKIN
50kd
37kd
GADD34
50kd
TUBULIN

## Slide 15
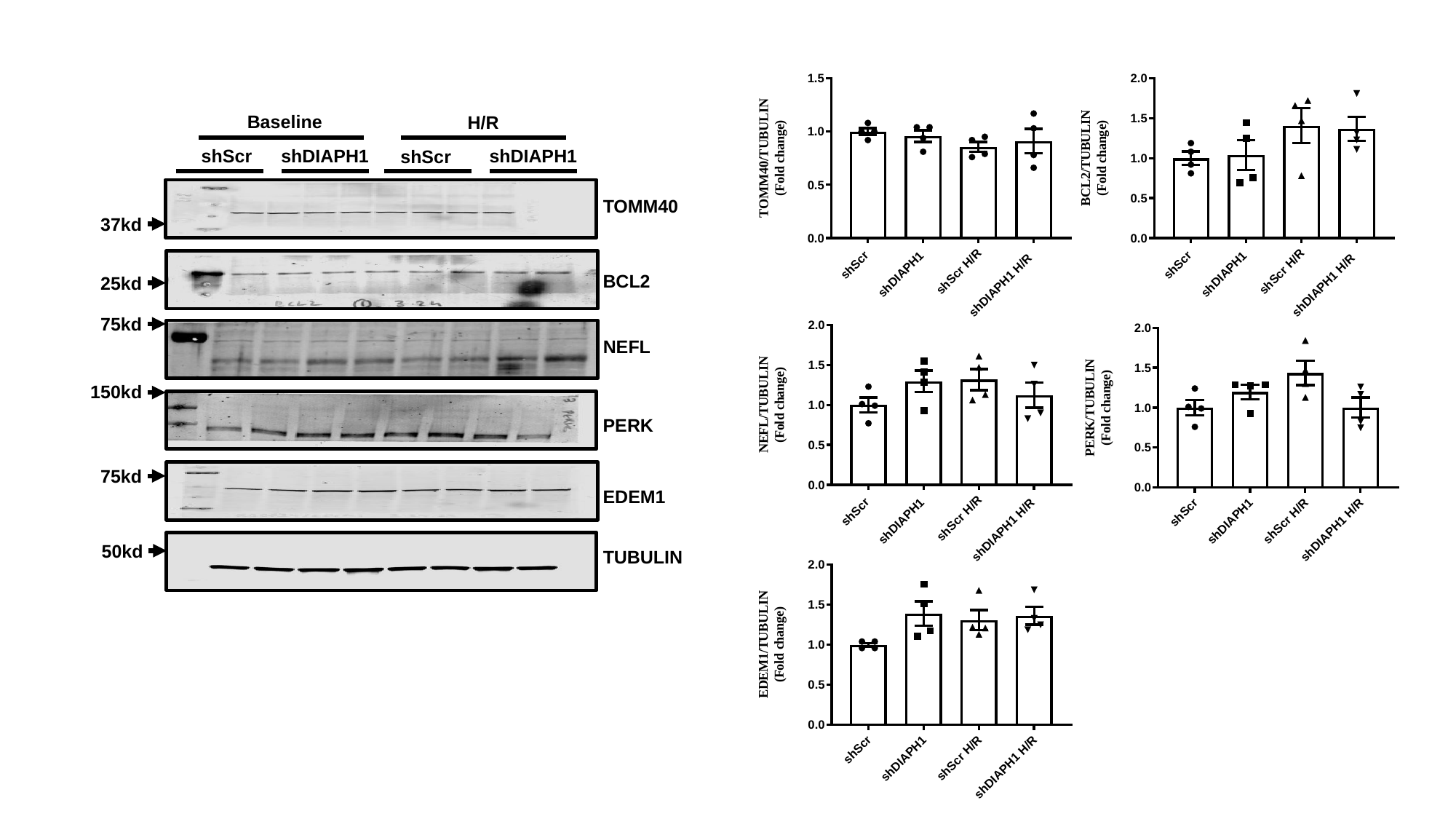

Baseline
H/R
shScr
shDIAPH1
shDIAPH1
shScr
TOMM40
37kd
BCL2
25kd
75kd
NEFL
150kd
PERK
75kd
EDEM1
50kd
TUBULIN

## Slide 16
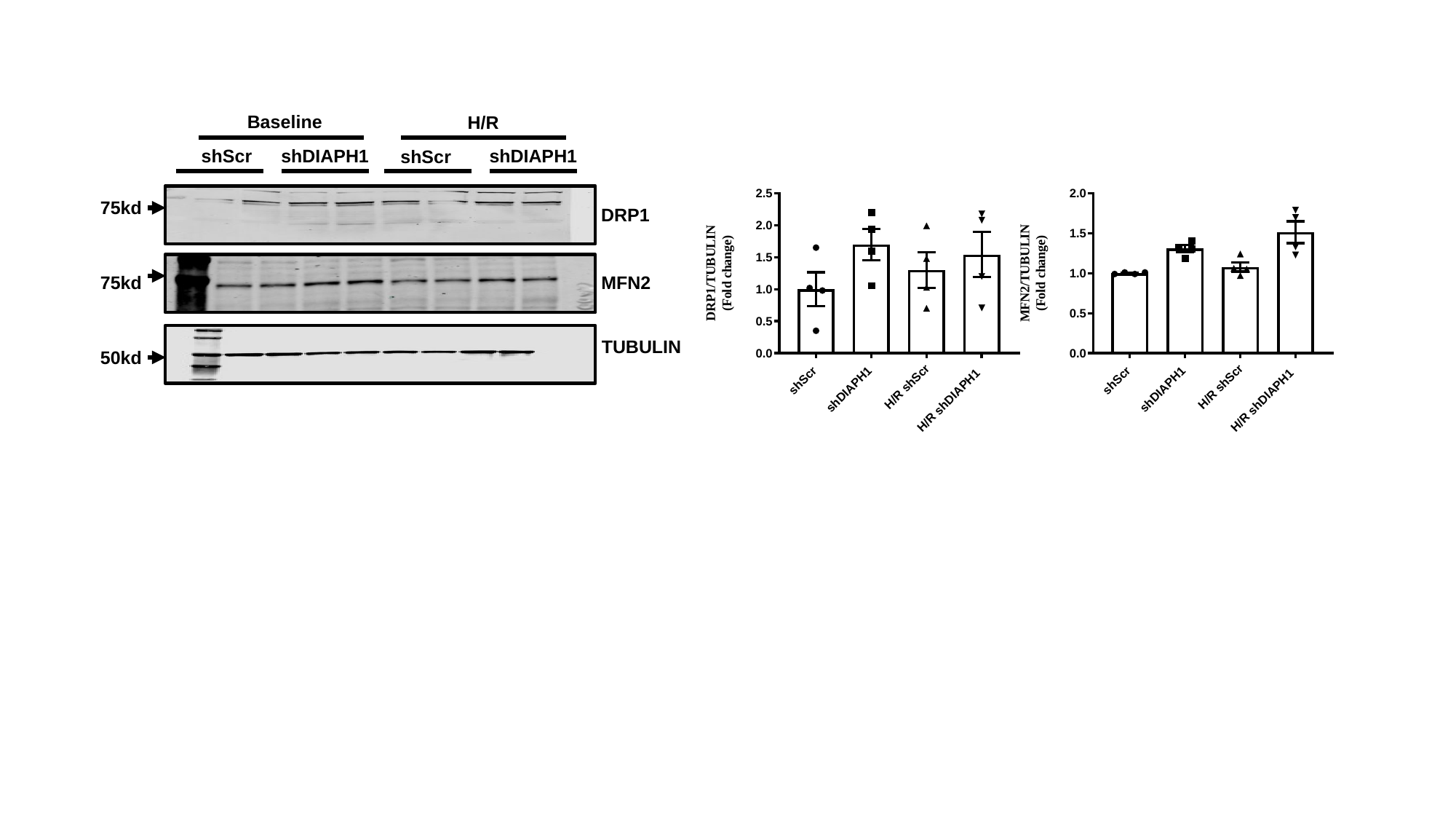

Baseline
H/R
shScr
shDIAPH1
shDIAPH1
shScr
75kd
DRP1
75kd
MFN2
TUBULIN
50kd
